# Marker genes reveal dynamic features of cell evolving processes

**DOI:** 10.1093/bioadv/vbaf185

**Published:** 2025-08-05

**Authors:** Wenjie Cao, Bengong Zhang, Tianshou Zhou

**Affiliations:** School of Mathematics, Sun Yat-sen University, Guangzhou 510275, China; School of Mathematics & Statistics, Wuhan Textile University, Wuhan 430200, China; School of Mathematics, Sun Yat-sen University, Guangzhou 510275, China

## Abstract

**Motivation:**

Embryonic cells finally evolve into various types of mature cells, where cell fate determinations play pivotal roles, but dynamic features of this process remain elusive.

**Results:**

We analyze four single-cell RNA sequencing datasets on mouse embryo cells, mouse embryonic fibroblasts, human bone marrow, and intestine organoid. We show that key (high expression) genes of each organism exhibit different statistical features and expression patterns before and after branch, e.g. for mouse embryo cells, the mRNA distribution of gene Gata3 is bimodal before branch, unimodal at branching point and trimodal for one branch but bimodal for the other branch. Moreover, there is a distribution mode such that it is the same before and after branch, and this fact would account for maintenance of the genetic information in a complex cell evolving process. Machine learning reveal that along the cell pseudo-time trajectory, the strength that one key gene regulates another is fundamentally increasing before branch but is always monotonically increasing after branch; burst size and frequency of key genes are always monotonically decreasing before branch but monotonically increasing for one branch and monotonically decreasing for another branch. Our results unveil the essential features of dynamic cell processes and can be taken as a supplement for accurately screening marker genes of cell fate determination on basis of the existed methods.

**Availability and implementation:**

The implementation of CFD is available at https://github.com/cellwj/CFD and the preprocessed data is available at https://zenodo.org/records/14367638.

Cell fate determination, single-cell RNA sequencing data, marker gene, cell process, developmental branch.

## 1 Introduction

Cellular processes such as proliferation, differentiation, and reprogramming are governed by complex gene regulatory programs, each cell makes its own fate decisions by integrating intracellular and extracellular signals and executing a complex choreography of gene regulatory changes (Macaulay *et al.* 2016, Cao *et al.* 2020, Qin *et al.* 2021, Qu *et al.* 2025). A more complex factor is cell communication through which cells respond to changing environments for better survival. Since the structure of a multicell tissue is tightly linked with its function, revealing the integrative mechanism of cell fate decisions from gene (microscopic) to gene network (mesoscopic) and to cell (macroscopic) levels is important yet challenging.

Single-cell RNA sequencing (scRNA-seq) technologies (Chu *et al.* 2016, Cao *et al.* 2019) can generate a “map” of cell states in a tissue with thousands or even tens of thousands of cells at relatively low cost, and interrogate the transcribed genes that define these states, provide an unprecedented opportunity to elucidate developmental pathways and to reveal cell fate decisions (Senapati *et al.* 2023). Interestingly, several efficient algorithms have been developed to order single cells of scRNA-seq data based on transcriptomic divergence (Peccoud and Ycart 1995, Paulsson and Ehrenberg 2000, Dar *et al.* 2012, Corrigan *et al.* 2016, Tantale *et al.* 2016, Nicolas *et al.* 2017, Lammers *et al.* 2020, Tunnacliffe and Chubb 2020, Pimmett *et al.* 2021, Chen *et al.* 2024). For example, Wishbone can reconstruct branching developmental trajectories from scRNA-seq data, where bifurcation points were identified by labeling each cell as prebifurcation or as one of two postbifurcation cell fates (Setty *et al.* 2016); Topographer can robustly identify any cell fate decision points and constructed a “quantitative” Waddington’s landscape of scRNA-seq data (Zhang *et al.* 2019). These approaches provide a global view for cell developmental processes. However, many important issues are still unsolved, e.g. what molecular mechanisms govern cell fate determinations, whether there are any changes in gene expression patterns before and after branches, and whether there are significant differences in molecular mechanisms underlying different expression patterns of key genes before and after branches. To address these issues, it needs to devise an integrative analysis approach to reveal the essential mechanisms of cellular programs governing cell fate decisions from scRNA-seq data lacking temporal information (Paulsson 2005).

Here we address the above issues by analyzing dynamic features of cell processes from the viewpoints of gene expression, gene-gene interaction, and cell process. For this, we analyze four sets of scRNA-seq data on developmental processes of mouse embryo (ME) cells, mouse embryonic fibroblasts (MEF), human bone marrow (HB), and intestine organoid (IO). Interestingly, we find that key (i.e. high expression) genes in each model organism exhibit qualitatively different expression patterns and statistical features before and after developmental bifurcations. Moreover, the emerging unimodal mRNA distribution and multimodal (bimodal or trimodal) mRNA distribution can well fit a Gamma distribution and a linear combination of several (two or three) Gamma distributions, respectively. For each marker gene, there is always an mRNA distribution mode such that it is the same before and after branch. Along the reconstructed cell pseudo-trajectory, the strength that one key gene regulates another is either monotonically increasing or kept constant before branch, but is always monotonically increasing after branch; burst size and frequency of each key gene are always monotonically decreasing but monotonically increasing for one branch and monotonically decreasing for the other branch. These results unveil the essential features of dynamic cell processes and can be in turn taken as a supplement of some screening methods such as Seurat (Butler *et al.* 2018) for accurately screening marker genes of cell fate determination. Different from previous studies which used single models to infer molecular mechanisms (Kim and Marioni 2013, Jiang *et al.* 2017, Larsson *et al.* 2019), our integrative analysis approach uses cell developmental stage-dependent models to infer molecular mechanisms, thus providing a paradigm for studying the global and dynamic features of cell fate determinations based on scRNA-seq data.

## 2 Methods

**Figure 1.**
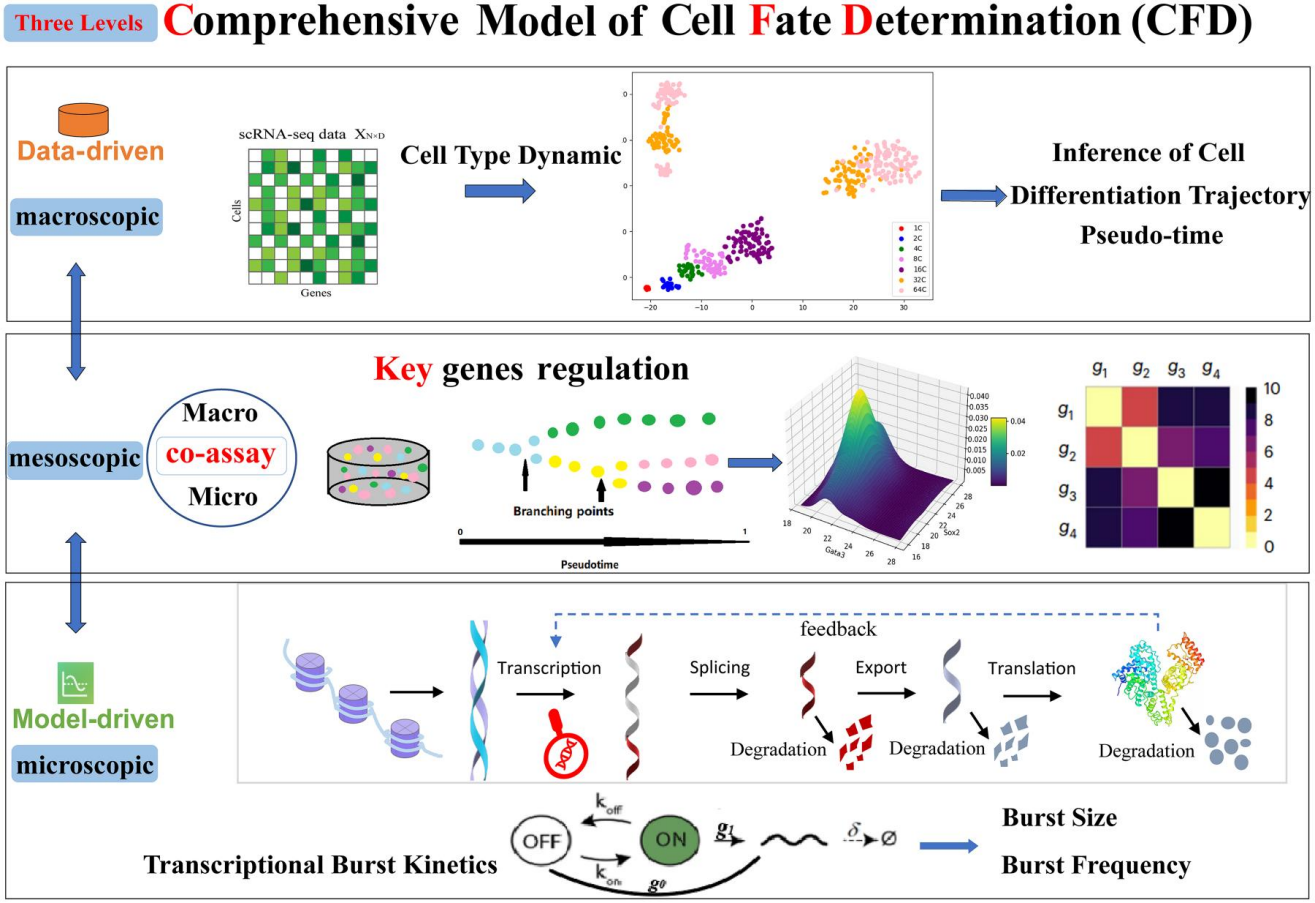
The framework of three-level model and a brief description of the major steps, which considers the balance and coordination of data driven and model driven, is proposed to reveal the global characteristics and the mechanisms of cell-type dynamics and transcriptional burst dynamics of four different types of datasets.

The summary information of the four scRNA-seq datasets to be analyzed in this article is shown in Table 1. The ME dataset, which consists of 438 cells and 48 genes, corresponds to the work of Wei *et al.* (2021). The MEF dataset, which includes 413 cells and 2162 genes, is from Luo *et al.* (2023). The HB dataset, which includes 5780 cells and 14 319 genes, is from Setty *et al.* (2019), and the IO dataset, which comprises 3831 cells and 9157 genes, are collected from Battich *et al.* (2020). We use these datasets to analyze dynamic characteristics of cell evolutionary processes from the viewpoints of gene expression (microscopic), gene-gene interaction (mesoscopic), and cell-type dynamics (macroscopic). Figure 1 depicts our integrative analysis framework, which is actually a combined method of data driven and model driven. For better understanding, we introduce the corresponding analysis approaches separately.

**Table 1.** Summary information of the four datasets used in this study.

Dataset	Cell count	Gene count	Web link
ME	438	48	http://github.com/gcyuan/SCUBA/tree/master/sample_data/guo2010
MEF	413	2162	https://github.com/cellfate/BurstFeedback/tree/main/code_Inference/Hierarchical_model/data
HB	5780	14319	https://ndownloader.figshare.com/files/27686835
IO	3831	9157	https://github.com/StatBiomed/UniTVelo

Note that a given set of scRNA-seq data correspond to a M×N matrix, where M stands for the number of cells and N for the number of genes.

### 2.1 Ordering cells in a given scRNA-seq dataset

We use the MLBKFD method to order single cells in the scRNA-seq datasets of ME cells and MEF, but apply the scVelo method (which considers the RNA velocity) to construct cell pseudo-time trajectories in the scRNA-seq datasets of HB and IO (Bergen *et al.* 2020, Han *et al.* 2024). Since these ordering methods, which can treat not only single cell trajectories but also branching cell trajectories, are extensively used, here we do not tend to introduce their details.

### 2.2 Calculating mRNA distributions

After having ordered all the single cells in a scRNA-seq dataset, we can easily calculate the mRNA distribution denoted by $P_{i}\left(m\right)$ along a single pseudo trajectory (possibly a branched trajectory) (see Fig. S1 for the calculation method, available as supplementary data at *Bioinformatics Advances* online), where every pseudo-time point in calculation is actually a pseudo-time window. Note that distribution $P_{i}\left(m\right)$ possibly has a different mode (e.g. unimodal, bimodal, or trimodal mode) but the distribution of the same mode would have different skewness and kurtosis, depending on a single pseudo-time trajectory).

### 2.3 Model selection

In general, a fundamental principle for model selection is to choose a simplest model for a specific “phenomenon” or “observation.” On the other hand, the common two-state model of gene expression has the potential to produce unimodal or bimodal distribution but cannot produce trimodal distribution. Therefore, to infer the most possible yet simplest molecular mechanisms governing the patterns of gene expression from scRNA-seq data, one may select two kinds of models: two-state models of gene expression without and with promoter leakage. Note that for each of the above four scRNA-seq datasets, the mRNA distributions of marker genes are only the one of undimodal, bimodal, and trimodal distributions, so it is no need to consider models of gene expression with more complex promoter structure. Selecting the simplest models may bring conveniences for the machine learning of molecular mechanisms.

**Figure 2.**
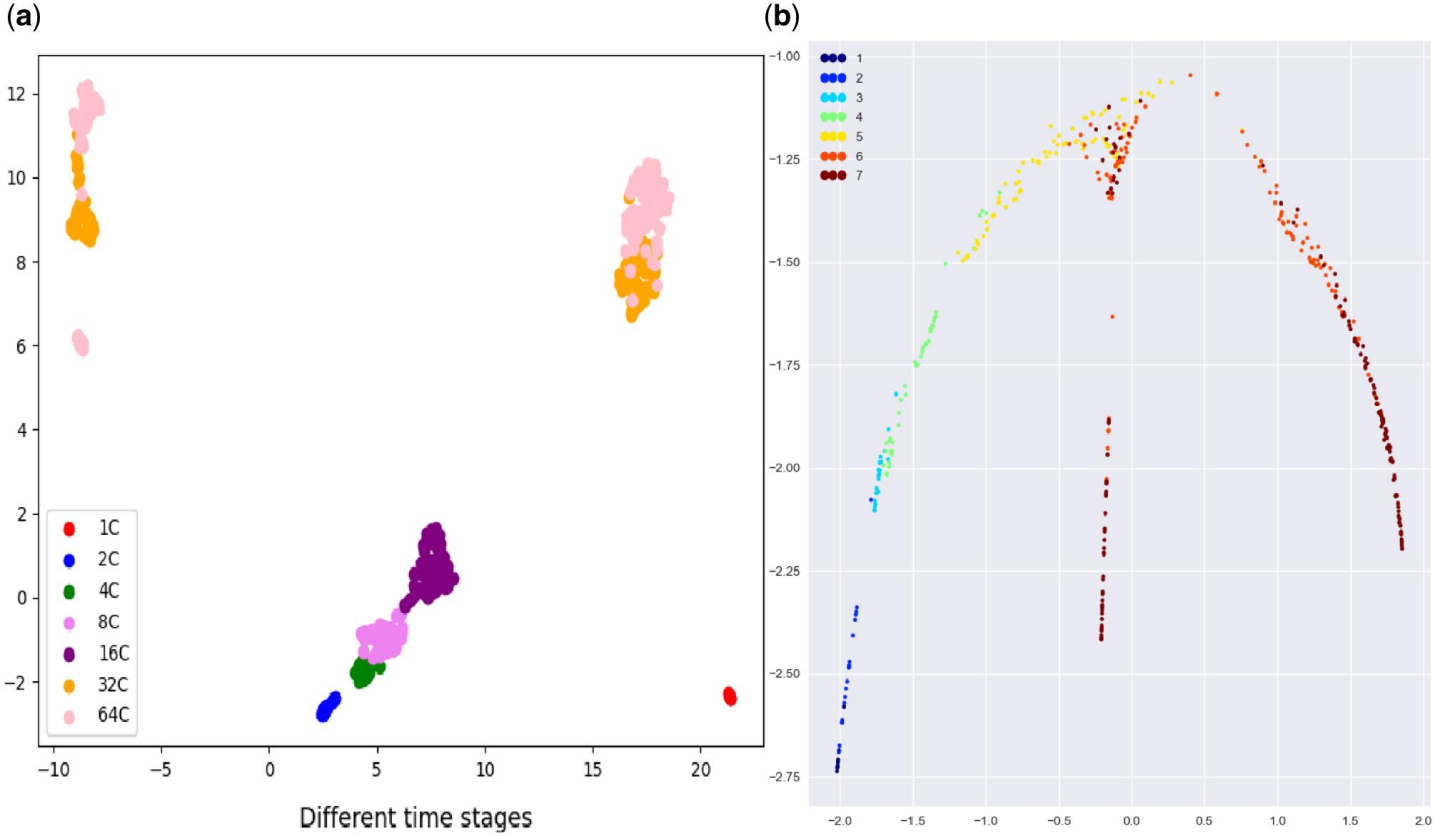
Visualization of clustering and branching trajectories for mouse embryo cells: (a) the clustering result obtained by the UMAP method; and (b) the clustering result for cell evolving stages.

**Figure 3.**
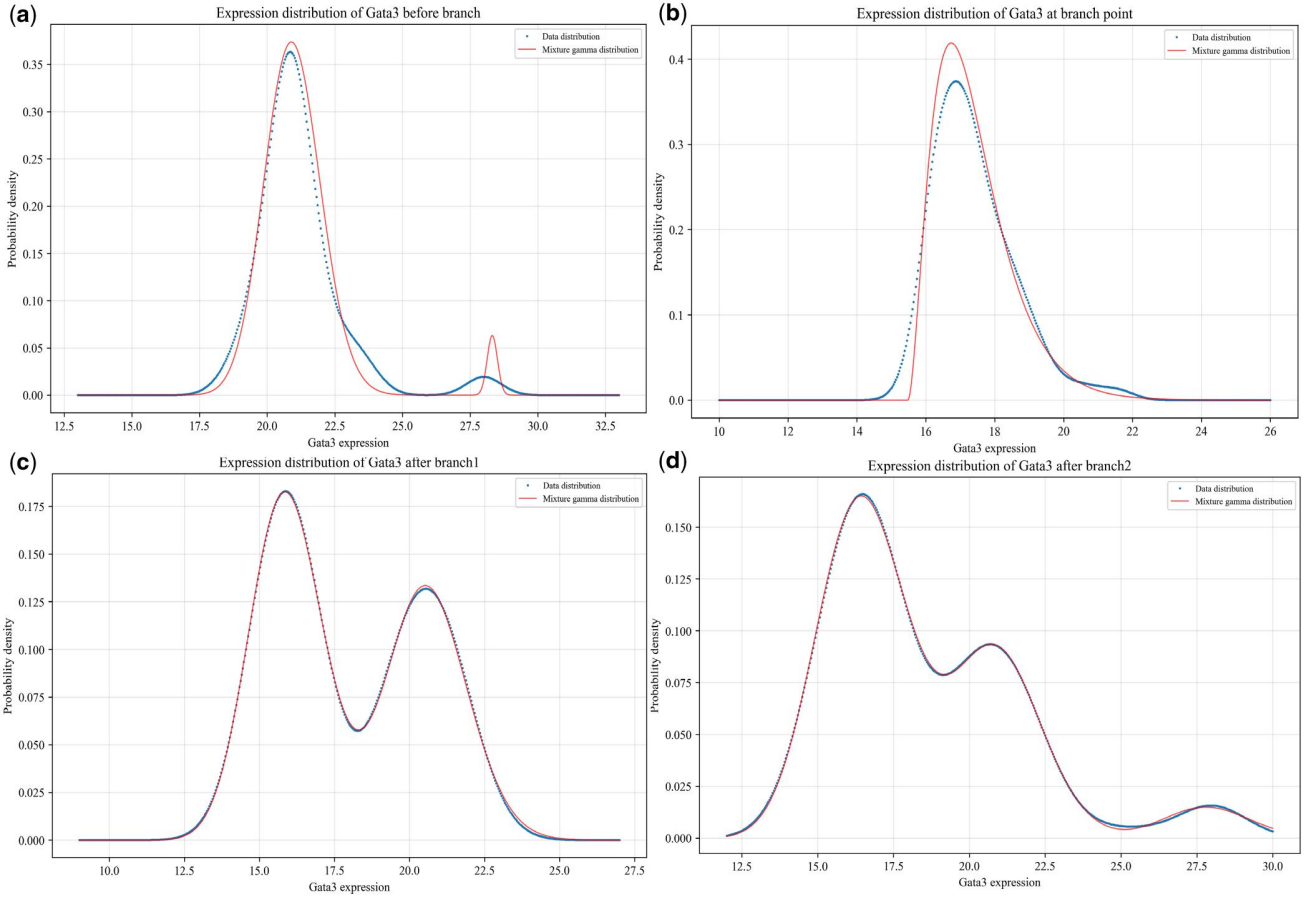
Characteristics of the mRNA distributions for key gene Gata3 before and after branch as well as at branching point: (a) before branch; (b) at branching point; (c) for one branch; (d) for the other branch. The blue dashed curves represent the distributions of data points while the red curves represent the fitting results obtained using mechanic models of gene expression.

We select an appropriate gene-expression model according to the mRNA distribution mode for a single branch of the cell pseudo-trajectory. Specifically, for each branch before and after the branch point, we can in principle use the following differential equation:


(1) $$\frac{dy}{dx}=f(x)+\varepsilon$$

to infer the molecular mechanism from the scRNA-seq data, where the function f(x) may be learned from the corresponding data, and ε is a random term (representing errors). If the corresponding mRNAs follow a unimodal or bimodal distribution as shown in Fig. 2b or 3b, then we select the following two-state model of gene transcription:


(2) $$\begin{array}{c} \text{OFF}\overset{\alpha }{\to }\text{ON},\;\;\;\text{ON}\overset{\beta }{\to }\text{OFF}\\ \text{ON}\overset{\mu }{\to }\text{ON}+\text{mRNA}\\ \text{mRNA}\overset{\delta }{\to }\emptyset \end{array}$$

where α and β are switching rates between two states of the promoter μ and δ are the transcription and degradation rates of the mRNA. Without loss of generality, we assume δ=1. Under some assumed conditions of model parameters, we can derive the analytical expression of the stationary mRNA distribution (Supporting Material, available as supplementary data at *Bioinformatics Advances* online)


(3) $$P\left(x|\alpha ,\beta \right)=\frac{{\tilde{\beta }}^{\alpha -1}}{\Gamma \left(\alpha \right)}x^{\alpha -1}e^{-\tilde{\beta }x}$$

where Γ(α) is the common Gamma function, $\tilde{\beta }=\mu /\beta$ is a new parameter, and x represents the mRNA concentration. This is a Gamma distribution and is unimodal (Fig. S2a, available as supplementary data at *Bioinformatics Advances* online). We find that the linear combination of two Gamma distributions can be used to well fit a bimodal distribution (Fig. S2b, available as supplementary data at *Bioinformatics Advances* online).

### 2.4 Parameter identification

For the mRNAs of a marker gene, we have an ‘experimental’ (or ‘data’) distribution $P^{\text{data}}\left(Y|X\right)$ (where X represents “mechanism” and Y represents “data”) and a theoretical (or mechanism) distribution $P^{\text{mech}}\left(m|\vartheta \right)$ (which is practically a conditional distribution, where ϑ is a parameter vector of the selected model). Based on these two distributions, we use the Kullback-Leibler divergence to determine the parameter vector ϑ. This needs to solve the following optimization question


(4) $$\hat{\vartheta }=\underset{\vartheta }{\arg}\min\left(KL\left(p_{Y|X}^{\text{data}}\left(m\right)|p_{X|\vartheta }^{\text{mech}}\left(m\right)\right)\right)=\underset{\vartheta }{\arg}\min\int p_{Y|X}^{\text{data}}\left(m\right)\;\text{log}\;\frac{p_{Y|X}^{\text{data}}\left(m\right)}{p_{X|\vartheta }^{\text{mech}}\left(m\right)}dm$$

where $P\left(m|\vartheta \right)=\sum_{n=0}^{\infty }p_{Y|X}^{\text{data}}\left(m\right)\cdot p_{X|\vartheta }^{\text{mech}}\left(n\right)$. Note that for the ON-OFF model, the parameter vector is ϑ=(α,β/μ).

### 2.5 Characterization of transcriptional bursts

Bursting kinetics are usually quantified by burst size and frequency. Note that the distribution for the number of transcripts ($X_{ij}$) of gene i in cell j is as follows


(5) $$\begin{array}{c} X_{ij}:\text{Poisson}\left(s_{j}{k^{\prime }}_{\text{syn},i}p_{i}\right)\\ p_{i}:\text{Beta}\left({k^{\prime }}_{\text{on},i},{k^{\prime }}_{\text{off},i}\right) \end{array}$$

where $s_{j}$ is the cell size, ${k^{\prime }}_{x,i}={k^{\prime }}_{x,i}/\left(\Phi_{i}+\alpha \right)$ represents the synthesis of gene i and the promoter switching rate measured by the actual degradation rate. The latter includes the gene-specific degradation rate $\Phi_{i}$ of the population and the exponential growth rate α. We use the neural network-based direct likelihood free inference (an extensive deep neural network with default parameter settings) (Tang *et al.* 2023) to infer the burst parameters BS and BF. Burst size or transcriptional efficiency ($k_{\text{syn},\;i}^{\text{eff}}/k_{\text{off},\;i}$) and the frequency at which bursts occur per unit time (burst frequency, $k_{\text{on},i}$).

### 2.6 Selection of marker genes

Selecting marker genes from scRNA-seq data needs criteria (Supporting Material, available as supplementary data at *Bioinformatics Advances* online), e.g. a marker gene should first be a highly variable gene (HVG). We use the Seurat method to select HVGs with a threshold of 30 in the scRNA-seq dataset of ME cells. Additionally, we impose the constraint that the expression distribution before and after branch must show a significant difference. Only those genes that meet these criteria can be considered as marker genes.

By analyzing the ME scRNA-seq dataset, we find that the identified marker genes before branch are expressed in a bursty manner, i.e. the produced mRNAs follow a gamma distribution that corresponds to a two-state model. After branch, the mRNAs are produced also in a bursty fashion, but the number of mRNA molecules follows a mixed Gamma distribution, which corresponds to a two-state model (note that for each of the two differentiated paths, the mRNAs follow the same bimodal distribution but with different skewness and kurtosis). Table S1 (available as supplementary data at *Bioinformatics Advances* online) lists the identified marker genes in the ME dataset, all of which exhibit the above expression distribution characteristics and also meet the HVG criteria. After taking the intersection, we obtained 11 marker genes for further analysis. In addition, we found that for a small size of this dataset, the Seurat method could not identify marker genes but the findMarker functions showed that all the genes were marker ones (Butler *et al.* 2018). Therefore, our method can identify marker genes in smaller datasets, and the identified marker genes along a differentiation trajectory are more specific than those identified by Seurat based on clusters.

## 3 Results

Here we mainly show the results obtained by analyzing the scRNA-seq data on ME cells. For the other three model organisms, the main results are demonstrated in Supporting Information, available as supplementary data at *Bioinformatics Advances* online.

As is known, Gata3 and Sox2 are two key genes that play an important role in the developmental process of ME cells, including cell fate decisions. First, the Gata3 protein is a critical transcription factor with various important functions in the development of ME cells. Second, the Sox2 gene provides instruction for making a protein that plays a critical role in the formation of many different tissues and organs during embryonic development. Therefore, we will use results obtained by analyzing the expressions of genes Gate3 and Sox2 to demonstrate dynamic characteristics of the evolutionary process of the ME cells (Sarkar and Hochedlinger 2013), functional information about key genes is shown in the Supporting Information, available as supplementary data at *Bioinformatics Advances* online.

### 3.1 Identifying cell types and reconstructing cell trajectories

Identifying cell types and reconstructing cell trajectories are the first yet important step toward to understanding cell evolutionary processes including cell fate decisions. Figure 2 demonstrates the cell pseudo-time trajectory for the development of ME cells (see Section 2 for the reconstruction method), where Fig. 2a shows the clustering results obtained by the UMAP clustering method (Becht *et al.* 2018).

From Fig. 2, we observe that cells in the scRNA-seq dataset of the ME cells are approximately categorized into three large clusters, in agreement with a previous result (Zhang *et al.* 2019). However, from the viewpoint of cell evolutionary stages, these cells can be divided into seven clusters (each cluster is called a stage cluster) as indicated in Fig. 2b. The first four stage clusters (indicated by numbers 1–4 in the figure) are prebranch clusters, cluster five is a cluster at the branching point, and the yellow and pink clusters represent two different clusters after branch.

For the other three model organisms, we also perform cluster analysis, and the results are simply summarized as follows. The cells in the scRNA-seq dataset of MEF can be classified into 10-stage clusters (referring to Fig. S1 in Supporting Information, available as supplementary data at *Bioinformatics Advances* online) (Pearson 1901, Van der Maaten and Hinton 2008, Becht *et al.* 2018). The cells in the scRNA-seq dataset of the HB can be divided into 10 clusters and undergoes two bifurcations that occur at clusters one and four. See Fig. S8 in Supporting Information, available as supplementary data at *Bioinformatics Advances* online, for details. Finally, the cells in the scRNA-seq dataset of the IO undergo a common bifurcation process as seen in most cells; The branching process looks like a reverse bifurcation process, which we refer to as an aggregation process; Based on cell development’s pseudo-time and the cluster information, the cells aggregate from multiple clusters into a single cluster. See Fig. S17 in Supporting Information, available as supplementary data at *Bioinformatics Advances* online, for details.

### 3.2 Key genes exhibit different expression patterns governed by different molecular mechanisms before and after branch

Cell pseudo-time trajectories only reflect the macroscopic process of cell evolution. A natural issue is how the expression levels of key genes (e.g. the Gate3 and Sox2 genes analyzed above) change along the cell pseudo-time trajectories (including branched trajectories). More specifically, does whether the same gene exhibit different expression patterns before and after branch, and what different molecular mechanisms shape different expression patterns?

In order to address the above questions, we only illustrate the results obtained by analyzing the scRNA-seq data of ME cells. Figure 3 demonstrates the mRNA distributions of key gene Gata3 before branch, at the branching point and after branch. We observe that the mRNA number follows a bimodal distribution before bifurcation (Fig. 3a), a unimodal distribution at the branching point (Fig. 3b), and a bimodal distribution for one branch (Fig. 3c) but a trimodal distribution for the other branch (Fig. 3d). Note that the mRNA distribution before bifurcation and the one for one branch after bifurcation are all bimodal. The maintenance of the shape of this distribution would imply that of the genetic information after cell division or differentiation. In other words, although many factors or processes (including division and differentiation as well as epigenetic modification) would affect the gene expression levels, the genetic information is still kept in the process of cell progression. However, the mRNA distributions of the same mode before and after bifurcation are essentially different since their skewness and kurtosis are distinct (see Tables 2 and 3). This difference would imply that the molecular mechanisms governing the mRNA distributions are different, or that some regulatory factors or processes are different. From the above observations, we conclude that key genes exhibit different expression patterns before branch, at the branching point and after branch, but there is also a branch such that the mode of the mRNA distribution for this branch is the same as that before branch.

**Table 2.** The inferred parameter values for the Gata3 gene before branch, at branching point, and after branch 1 and branch 2.

	Distribution type	parameter								
		ω1	ω2	α1	β1	α2	β2	α3	β3	loc
Before branch	Bimodal mRNA distribution	0.97		408.27	19.49	22349	789.19			
Branch point	Unimodal mRNA distribution			2.898	1.5106					15.481
Branch 1	Bimodal mRNA distribution	0.4426		240.7904	11.6749	170.3634	10.685			
Branch 2	Trimodal mRNA distribution	0.6275	0.3193	117.9348	7.1182	213.9376	10.1856	384.8679	13.8132	

**Table 3.** Statistical quantities for the Gata3 gene before branch, at branching point, and after branch 1 and branch 2.

	Peak position	Mean	Variance	Kurtosis			Skewness		
Before branch	20.826	20.952	1.089	0.718	−1.5		0.491	0.705	
	30.189	30.185	0.046						
Branch point	16.859			1.66			1.195		
Branch 1	15.859	15.893	0.480	−0.614	−0.683		0.115	0.107	
	20.527	20.560	0.822						
Branch 2	16.481	20.657	29.307	−0.72	0.951	2.858	−0.19	0.491	−2.155

**Figure 4.**
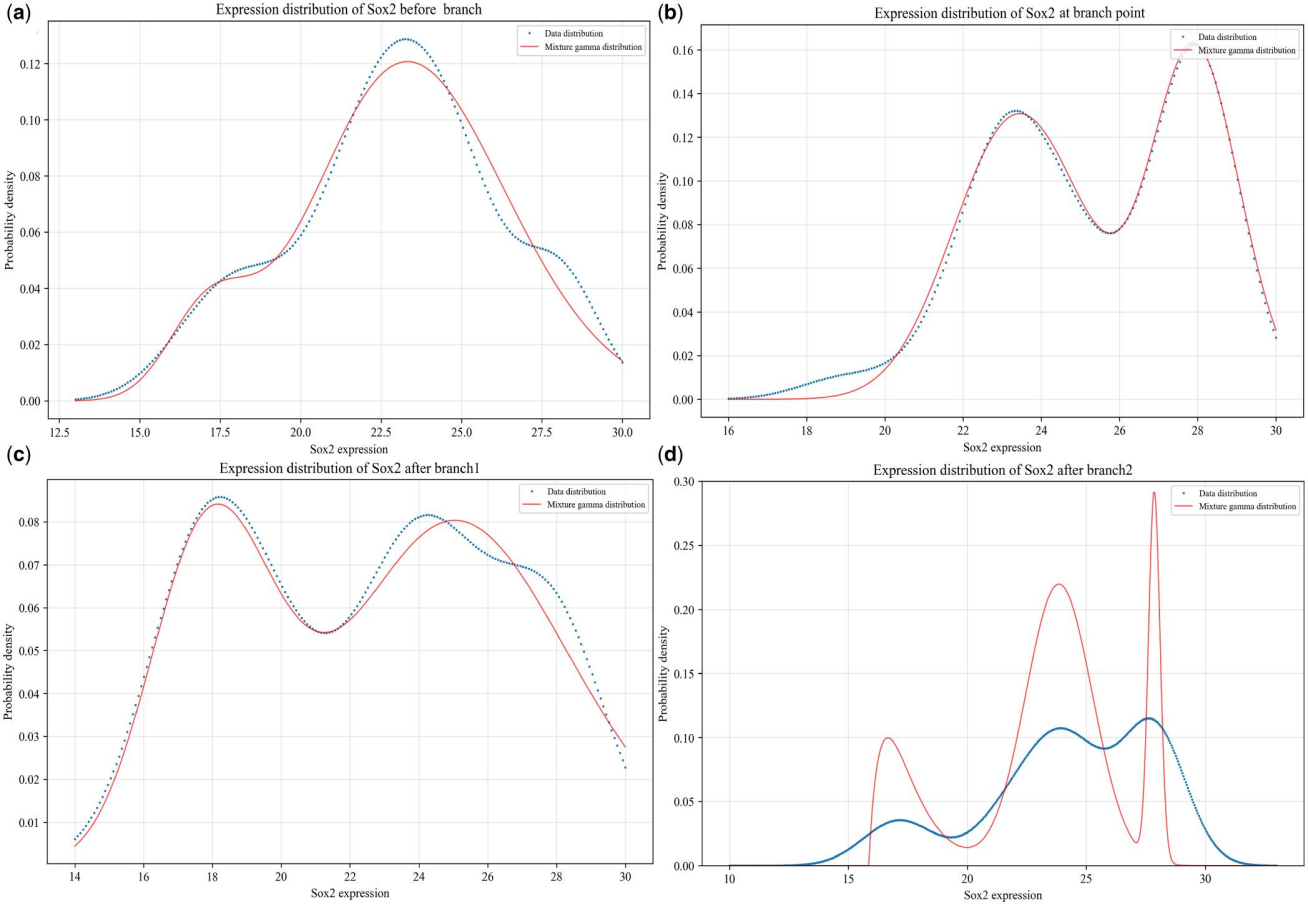
Different modes of the mRNA distributions for key gene Sox2 before and after branch as well as at branching point: (a) before branch; (b) at branching point; (c) for one branch; and (d) for the other branch.

For key gene Sox2, we also find the same laws as for gene Gata3, referring to Fig. 4 where Fig. 4a and b demonstrate that the mRNA distributions before and at bifurcation are all bimodal, whereas Fig. 4a and b demonstrate that the mRNA distributions for two branches after bifurcation are respectively bimodal and trimodal. Moreover, the mode of the mRNA distributions in Fig. 4a is the same as that in Fig. 4c. In order to verify whether there is definitely a branch such that the mode of the mRNA distribution for this branch is the same as that before branch, we also analyze the other three datasets. Some relevant analysis results of the MEF dataset are presented in the Supporting Material, available as supplementary data at *Bioinformatics Advances* online.

In order to reveal molecular mechanisms governing the expression patterns of key genes (e.g. genes Gata3 and Sox2 shown in Fig. 3), we choose the following Gamma distribution:


(6) $$P\left(x|\alpha ,\beta \right)=\frac{1}{\beta^{\alpha }\Gamma \left(\alpha \right)}x^{\alpha -1}e^{-x/\beta }$$

as a fundamental element of the mRNA distributions to be inferred from experimental data. Reasons for this choice are as follows. Most genes in eukaryotic cells are expressed in a bursty manner. A more important point is that the Gamma distribution has the following advantages: (1) it has the simplest form and can be derived from the common two-state model of gene transcription (implying that the corresponding molecular mechanism is clear) (Herbach *et al.* 2017). (2) In Equation (6), parameter β represents the average size of the transcription factor (TF) production bursts (i.e. the mean burst size), and parameter α represents the mean number of TF production bursts per cell (i.e. its inverse represents the mean burst frequency).

In our inference, we use the Gamma distribution given by Equation (6) to fit a unimodal distribution, and the linear combinations of Gamma distributions:


(7) $$P\left(x|\alpha_{i},\beta_{i},1\leq i\leq m\right)=\sum_{i=1}^{m}\frac{\omega_{i}}{\beta_{i}{}^{\alpha_{i}}\Gamma \left(\alpha_{i}\right)}x^{\alpha_{i}-1}e^{-x/\beta_{i}}$$

to fit multimodal (bimodal and trimodal) distributions, where $\omega_{i}$ are non-negative weighted factors, satisfying conservative condition: $\sum_{i=1}^{m}\omega_{i}=1$. For key gene Gata3, we find that the Gamma distribution can well fit the unimodal mode of the mRNA distribution (Fig. 3b), a linear combination of two Gamma distributions can well fit the bimodal mode (Fig. 3a and c), and another linear combination of three Gamma distributions can well fit the trimodal mode (Fig. 3d). The inferred parameter values are shown in Table 2, and statistical quantities are listed in Table 3. To that end, we can conclude that the Gata3 gene exhibits different expression patterns before and after bifurcation as well as at branching point, which would be governed by different molecular mechanisms.

Next, we show results for another key gene (i.e. the Sox2 gene) in ME cells. Figure 4 demonstrates the corresponding mRNA distributions before branch, at the branching point and after branch. Form this figure, we observe that the mRNA number follows a unimodal distribution before bifurcation (referring to Fig. 4a), a bimodal distribution at the branching point (Fig. 4b), and a bimodal distribution for one branched trajectory (Fig. 4c) but a trimodal distribution for the other branched trajectory (Fig. 4d). Similar to the case of the Gata3 gene, we find that for gene Sox2, the linear combination of two Gamma distributions can well fit the bimodal mode (Fig.4b and c), but a linear combination of three Gamma distributions can fundamentally match a trimodal mode, referring to Fig. 4d. From Fig. 4d, however, we observe a large difference between data and theoretical distributions, possibly because data points are too few. Table 4 shows the inferred parameter values, and Table 5 list statistical quantities. Then, we can conclude that the Sox2 gene also exhibits different expression patterns before and after bifurcation as well as at branching point, which would be governed by different molecular mechanisms. In addition, the same mode of the bimodal distributions at the branching point and after one branch would imply that the genetic information is kept constant in the complex cell evolutionary process. For the other three scRNA-seq datasets, we also find the similar observations, see Supporting Material, available as supplementary data at *Bioinformatics Advances* online for details.

**Table 4.** The inferred parameter values for the Sox2 gene before branch, at branching point, and after branch 1 and branch 2.

Distribution type		Before branch	Branch point	Branch 1	Branch 2
		Bimodal mRNA distribution	Unimodal mRNA distribution	Bimodal mRNA distribution	Trimodal mRNA distribution
parameter	ω1	0.8974	0.5602	0.3629	0.2269
	ω2				0.7731
	α1	62.9659	189.4045	100.726	1.9238
	loc1				15.8609
	β1	2.657	8.0356	5.5402	1.1556
	α2	178.2327	627.5364	63.7933	4434.7861
	loc2				−69.6437
	β2	10.3205	22.4168	2.5035	47.4206
	α3				2110.4189
	loc3				16.7186
	β3				189.3329

**Table 5.** Statistical quantities for the Sox2 gene before branch, at branching point, and after branch 1 and branch 2.

	Peak position	Mean	Variance	Kurtosis			Skewness		
Before branch	23.253	24.364	6.431	−0.496	7.35		−0.693	−2.809	
	28.002	19.616	5.478						
Branch point	23.33	27.959	0.094	1.839	4.633		−1.035	−2.057	
	27.893	23.439	2.639						
Branch 1	18.232	25.335	5.936	−0.779	−1.362		0.428	0.106	
	24.157	18.205	1.127						
Branch 2	17.154	23.791	2.555	2.42	−0.713	0.798	1.265	−0.453	−1.188
	23.943	27.949	0.129						
	27.634	17.134	0.527						

### 3.3 Key genes exhibit different correlations before and after branch

As a known fact of matter, gene regulatory networks govern cell evolutionary processes (or cell progression). Although the above analysis has shown that key genes exhibits different features before and after bifurcations as well as at the branching points, whether the interactions between these key genes also exhibit different characteristics before and after bifurcations.

**Figure 5.**
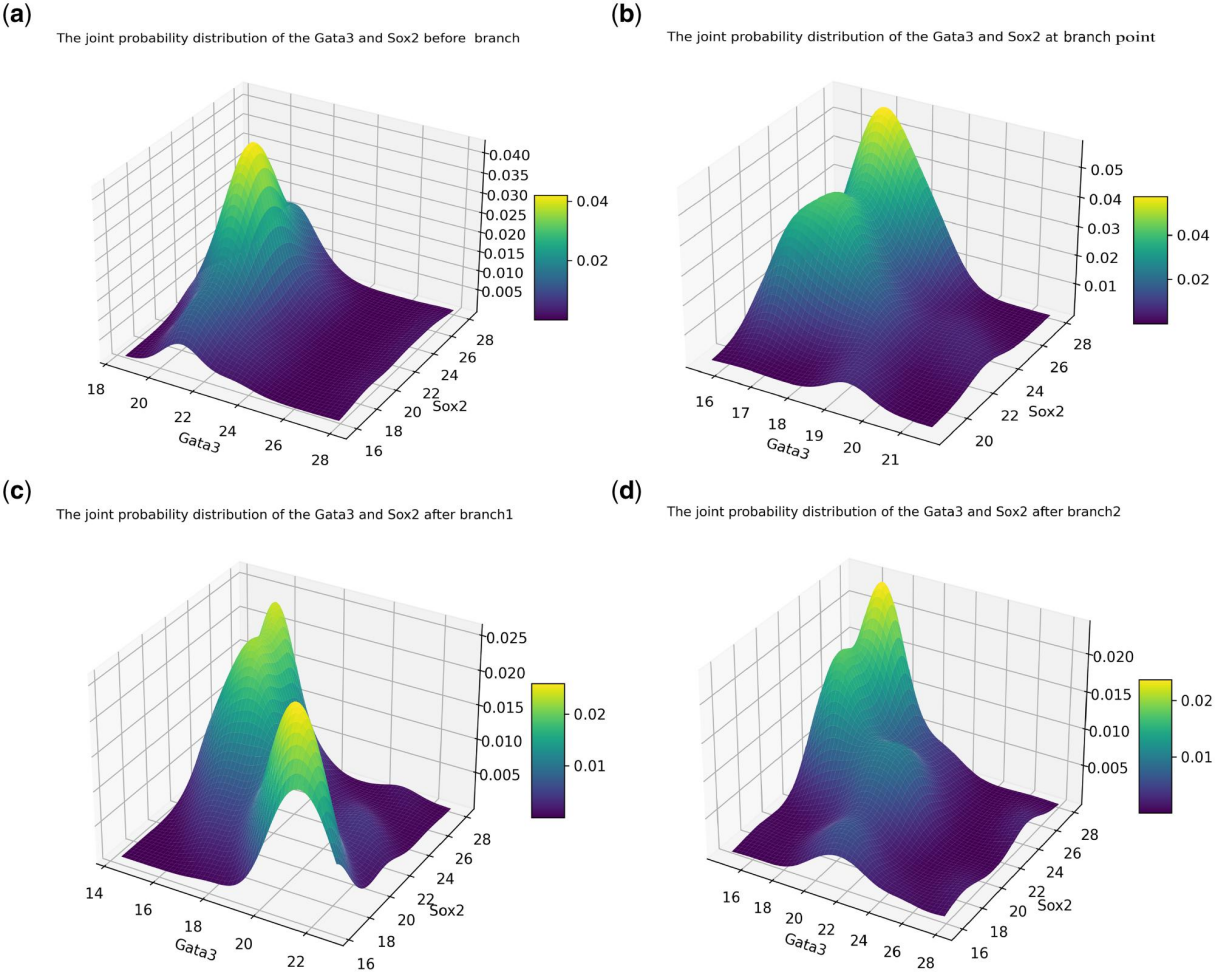
Changes in joint probability distribution of two key genes Gata3 and Sox2 before branch (a), at branching point (b) and after branch 1 (c) as well as after branch 2 (d) in the ME dataset.

In order to address this issue, we first calculate the joint probability density functions of two key genes Gata3 and Sox2. Figure 5 demonstrates the corresponding results before and after branch as well as at branching point. See Supporting Material, available as supplementary data at *Bioinformatics Advances* online for detailed analysis.

**Figure 6.**
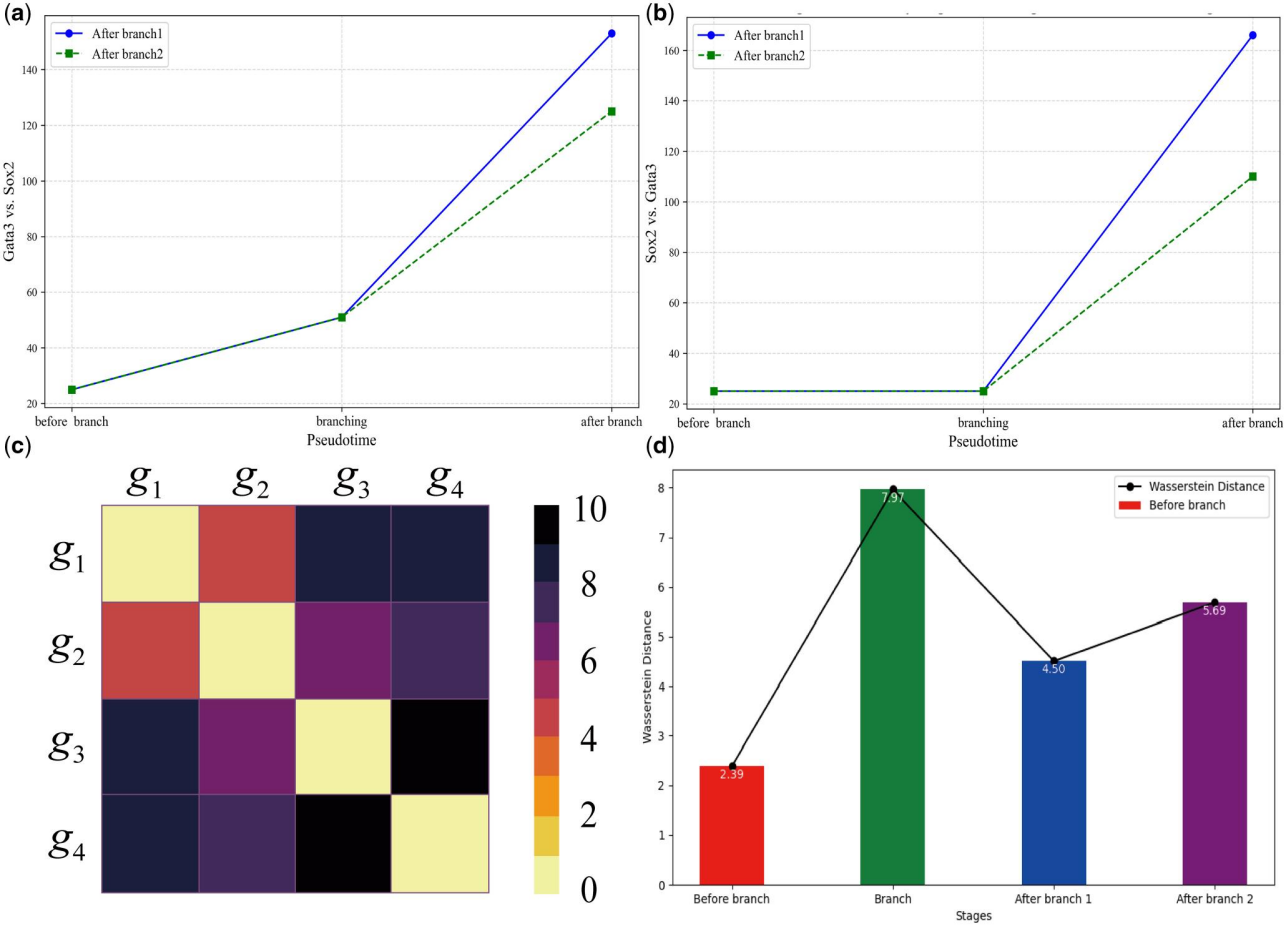
(a, b) Changes of the regulation strength along the pseudo-time trajectory of ME dataset, where (a) key Gene Gata3 regulates key gene Sox2; (b) Sox2 regulates Gata3. (c, d) Wasserstein distances between genes Gata3 and Sox2 at different cell stages, where (c) the Wasserstein distance matrix between Gata3 and Sox2; (d) the Wasserstein distance between Gata3 and Sox2 at different cell stages.

Next, we use GENIE3 to calculate, separately, the regulatory strengths of gene Gata3 to gene Sox2 and vice versa along the cell pseudo-time trajectory (Huynh-Thu *et al.* 2010, Bond *et al.* 2023). The corresponding results are demonstrated in Fig. 6a and b. Recall that the Wasserstein distance can characterize the similarity between two distributions, and in particular, this distance can capture the relationship between the genes along the pseudo-time trajectory. Specifically, if two genes are continuously activated in a biological process, the distribution shapes of their expressions should be highly similar, implying that there is a smaller Wasserstein distance between them. Here we calculate this distance between the mRNA distributions of key genes Gata3 and Sox2 in different cell branches of the ME dataset, with the results demonstrated in Fig. 6c and d.

### 3.4 Key genes exhibit different features of burst kinetics before and branch

In eukaryotic cells, most genes are expressed in a bursty fashion (Setty *et al.* 2019, Fawkner-Corbett *et al.* 2021, Fiorenzano *et al.* 2021), i.e. gene products are produced in episodes of high transcriptional activity followed by long periods of inactivity. This bursting kinetics is usually quantified by burst size and burst frequency. Therefore, we focus on the calculation of mean burst sizes and frequencies of key genes along the reconstructed pseudo-time trajectories.

**Figure 7.**
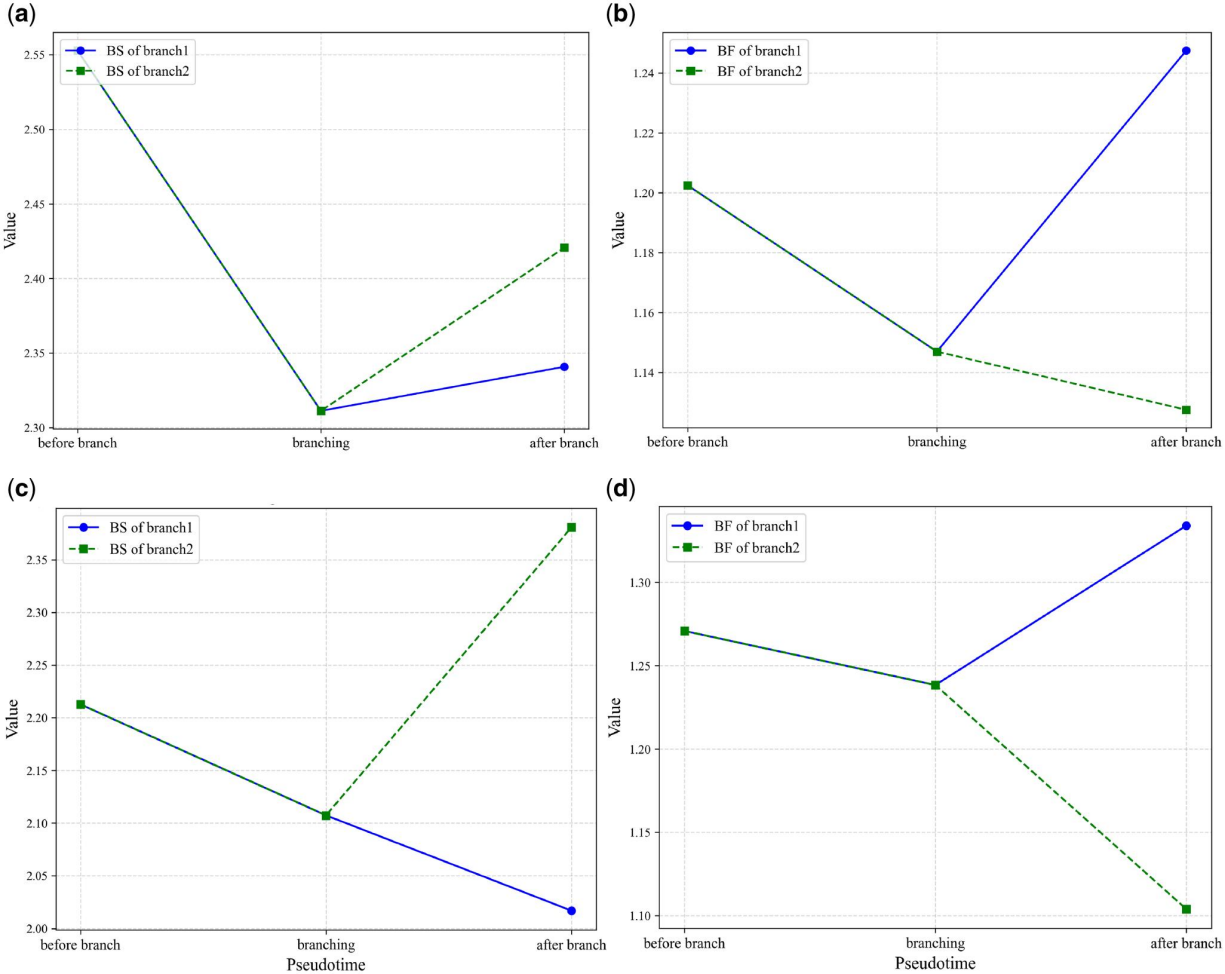
Changes in burst size and frequency of key genes Gata3 and Sox2 at different cell stages in the ME dataset: burst size (a) and burst frequency (b) of Gata3; burst size (c), and burst frequency (d) of Sox2.

Figure 7 depicts the changes in burst size and frequency of key genes Gata3 and Sox2 along the cell pseudo-time trajectory in the ME dataset. We observe that for gene Gata3, the burst size is monotonically decreasing before bifurcation, but is monotonically increasing after bifurcation refer-ring to Fig. 7a. Moreover, the burst size for one branch increases faster than the other branch, i.e. the burst size rapidly increases for one branch while slowly increases for the other branch (also see Fig. 7a). By contrast, the burst frequency is monotonically decreasing before bifurcation, but sharply increases for one branch and slowly decreases for the other branch, referring to Fig. 7b. These observations indicate that cell evolutionary processes can significantly affect bursting kinetics.

For key gene Sox2, the change tendency seems different from the case of key gene Gata3. Specifically, the burst size of gene Sox2 decreases more slowly before bifurcation in contrast to that of gene Gata3, but sharply increases for one branch and still slowly decreases for the other branch, referring to Fig. 7c. The burst frequency slowly decreases before bifurcation but sharply decreases for one branch and sharply increases for the other branch, referring to Fig. 7d.

By the combination of the analysis of the two key genes, we conclude that the increase or decrease of burst size and frequency after branch are cell type-specific, which is an interesting result.

## 4 Conclusion

Cell fate determinations play pivotal roles in cell evolutionary processes. In this article, we have used the data-driven and model-driven method to analyze dynamic characteristics of the entire cell evolving process based on four types of scRNA-seq datasets: ME cells, MEF, HB, and IO. We found that marker genes for each model organism exhibited different expression patterns, different statistical properties, and different correlations before and after branches as well as at branching points. Moreover, machine learning–based statistical inference revealed that different molecular mechanisms governed the expression patterns of the key genes at different stages of cell evolution. The revealed essential characteristics of dynamic cell processes can help us understand the complex processes of cell evolution.

Recall that previous inference approaches used single models to infer the genome-wide mechanisms of gene expression from scRNA-seq data (Luo *et al.* 2023). In contrast, our approach not only considered different stages of a cell process but also used different models of gene transcription for different expression patterns, and hence it can better reflect the biological reality of the cell evolving process behind the scRNA-seq data. It is worth pointing out that for each of the four scRNA-seq datasets analyzed here, the mRNA numbers of key genes follow the one of unimodal, bimodal and trimodal distributions at some cell stages, so we can successfully infer the corresponding molecular mechanisms from scRNA-seq data by the machine learning of simple rather than complex gene-expression models. For some scRNA-seq dataset, however, if the mRNA numbers of marker genes follow a multimodal distribution at some cell stages, our approach hints that one may use the combination of simple two-state gene-expression models to efficiently infer the underlying molecular mechanisms from scRNA-seq data. In the case of this distribution mode, if complex multistate gene-expression models (Zhang and Zhou 2014, Daigle *et al.* 2015, Perez-Carrasco *et al.* 2016, Jia and Li 2023, Zhou *et al.* 2024) are used in inference, then this would lead to difficulties and even failures of inference.

In a word, our approach proposed here can well reveal complex cell evolutionary processes including cell fate determinations. In particular, it would provide a paradigm for analyzing cell evolutionary processes based on scRNA-seq datasets in complex cases that more factors (e.g. gene regulation) are considered. We believe that our method would have a broad application perspective in many scientific fields involving the statistical inference of mechanisms from stationary data (or time-series data) to dynamic processes.

## Author contributions

Data analysis and drawings: Cao; Conceived and designed: Cao, Zhou; Writing: Cao, Zhang, Zhou.

## Supplementary Material

vbaf185_Supplementary_Data

## Data Availability

The implementation of CFD is available at https://github.com/cellwj/CFD and the preprocessed data is available at https://zenodo.org/records/14367638.
