# Supplementary material for: Marker genes reveal dynamic features of cell evolving processes
Source: Bioinform Adv. 2025 Aug 5;5(1):vbaf185. doi: 10.1093/bioadv/vbaf185 (PMC12396811; doi:10.1093/bioadv/vbaf185)
Supplement: vbaf185_Supplementary_Data [file vbaf185_supplementary_data.zip › Supplemental Material.docx]

**Supplementary Material**

Marker genes reveal dynamic features of cell evolving processes

Wenjie Cao, Bengong Zhang, Tianshou Zhou*

Corresponding authors: Tianshou Zhou, School of Mathematics, Sun Yat-sen University, Guangzhou 510275, China. Tel.:/Fax: +86-13710636599; E-mail: [mcszhtsh@mail.sysu.edu.cn](mailto:mcszhtsh@mail.sysu.edu.cn)

**Contents**

A. Derivation of mRNA distributions 1

B. Methods3

B1. Model selection 3

B2. Selection criteria for marker genes 3

C. Supplemental texts and figures4

C1. Figures and Tables related to the Method and Material in the main text 4

C2. Mouse embryo cells5

C3. Mouse embryonic fibroblasts6

C4. Human bone marrow12

C5. Intestine organoid18

D. Supplemental tables 26

References 28

A. Derivation of mRNA distributions

First, we consider the common two-state model of gene expression at the transcription level:

(A1)

where and are switching rates between two states of the promoter, and are the transcription and degradation rates of the mRNA. Without loss of generality, we assume . If the mRNA distribution at steady state is denoted by , then it can be analytically expressed as

, (A2)

where is a confluent hypergeometric function (Daalhuis,2010), and we have defined . Note that the expression of is very complex in form since it involves a confluent hypergeometric function of the form . This brings difficulties for the statistical inference of model parameters based on scRNA-seq data.

In the following, we simply derive an approximate distribution of mRNA. If the production rate is sufficiently large compared with the other reaction rates, then discrete variable can be taken as a continuous variable (re-denoted by ). Under this assumption, we can use a continuous equation to approximate :

, (A3)

where represents the random telegraph signal of gene activation (corresponding ) and inactivation (corresponding ). Note that the switching rates from inactivation and activation and vice versa are and respectively. For convenience, we introduce two functions, which are defined below

(A4)

where is an infinitesimal increment, and represent the probabilities that the value of mRNA is in active and inactive states respectively. Set , which is the total probability. Note that the probability flux can be expressed by synthesis and degradation rates combined with two factorial probabilities and , that is,

(A5)

Then, according to the conservative condition for probability flux, we have

. (A6)

This is a mathematical equation for a gene that has one active and one inactive states (this model can be easily extended to the case that a gene has multiple inactive and active states).

Next, we find the equilibrium solution to Eq. (A6). At steady state, the sum of two equations yields

, (A7)

from which we know that must be a constant independent of . Since both and tend to zero as goes to infinity, we have for any . Thus, we obtain the relationship between and : or . Making use of this relationship, it follows from Eq. (A6) that

. (A8)

By solving this equation, we arrive at

, (A9)

indicating that mRNA follows a Beta distribution. Apparently, may be unimodal or bimodal, depending on the choice of and values.

If the deactivation rate is much larger than the activation rate and is larger than the degradation rate , then distribution can be further approximated as the following Gamma distribution

, (A10)

which is determined by two parameters and (). Moreover, represents the average size of the transcription factor production bursts (i.e., the mean burst size) and represents the mean number of transcription factor (TF) production bursts per cell (i.e., the inverse of the mean burst frequency).

**B. Method**

**B1. Model selection**

We use the Kernel Density Estimation (KDE) method to perform estimations for each of the four datasets in the main text. As is well known, the KDE is a non-parametric statistical method and can be used to estimate an unknown probability density function. This method does not rely on any specific assumptions on probability distribution, so it can be applied to any type of dataset.

Given as  independent and identically distributed samples, if we let their probability density function be , then the KDE takes the form

(A11)

where  is the kernel function (non-negative, with the integral 1, satisfying the properties of a probability density and having the zero mean); is referred to as the bandwidth and is sometimes called a window in calculation.

**B2. Selection criteria for marker genes**

1. The gene should rank high in differential expression within the corresponding cell cluster.

2. The gene should exhibit strong expression specificity, meaning that it has a high expression rate in the corresponding cell cluster and a low expression rate in other clusters.

C. Supplemental texts and figures

We assume that for each dataset, the information on the entire development process is adequate, or a snapshot of primary tissue represents a complete developmental process.

**C1. Figures and Tables related to the Method and Material in the main text**


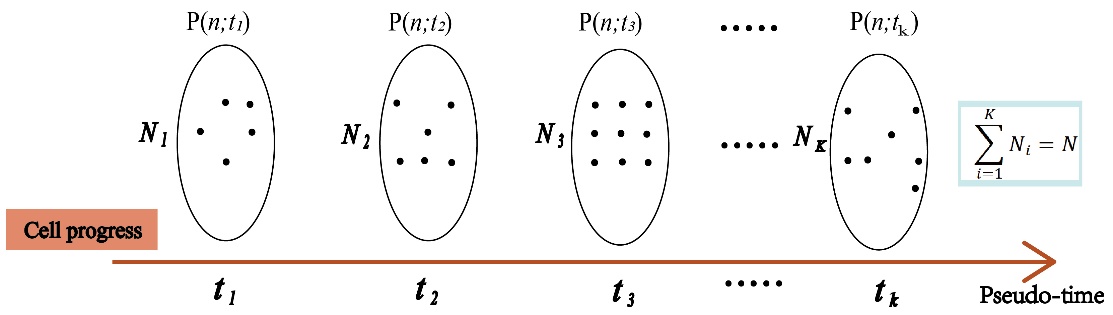


**Fig. S1** Calculation of the mRNA (the product of some key gene) distribution at time point (practically a time window) along the single pseudo-trajectory. Similarly, we can calculate distribution for the entire single pseudo-trajectory, which is actually a weighted mixture of the distributions at all pseudo-time points of this single pseudo-trajectory.

The linear combination of two Gamma distributions can be used to well fit a bimodal distribution (Fig. S2).


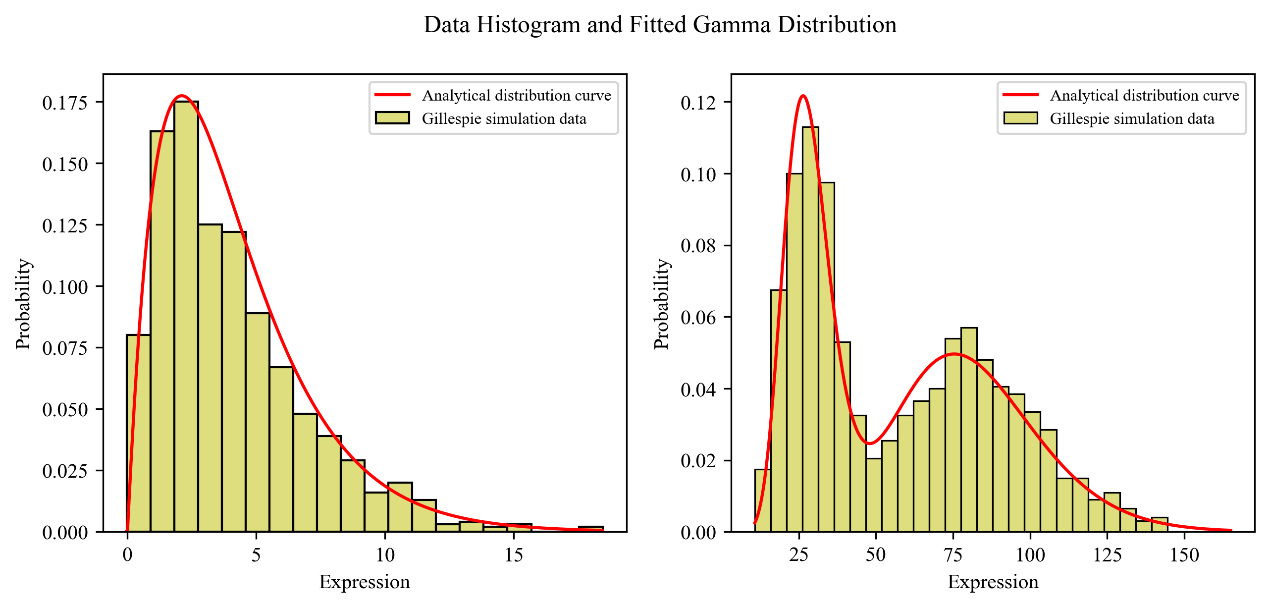


**Fig. S2** Unimodal and bimodal mRNA distributions, where histograms represent the numerical results obtained by the famous Gillespie stochastic algorithm whereas the red curves represent the results predicted by analytical distribution (Gillespie,1976). Parameter values are set as in the case of unimodal mode, and , in the case of bimodal mode.

**Table S1. Marker genes in mouse embryonic cells**

|  | Genes with different distribution characteristics before and after branching | Highly variable gene | Marker gene |
| --- | --- | --- | --- |
| Pdgfa | Y | — | — |
| Cdx2 | Y | — | — |
| Eomes | Y | — | — |
| Ahcy | Y | — | — |
| Lcp1 | Y | — | — |
| Fgfr2 | Y | — | — |
| Klf5 | Y | — | — |
| Actb | Y | Y | Y |
| Dab2 | Y | Y | Y |
| Dppa1 | Y | Y | Y |
| Nanog | Y | Y | Y |
| Gapdh | Y | Y | Y |
| Id2 | Y | Y | Y |
| Pou5f1 | Y | Y | Y |
| Tspan8 | Y | Y | Y |
| Sox2 | Y | Y | Y |
| Gata3 | Y | Y | Y |
| Sall4 | Y | Y | Y |

C2. Mouse embryo cells

The Gata3 plays a key role in the formation and differentiation of the endoderm and ectoderm, guiding cells towards specific fates by regulating the expression of target genes. Usually, the Gate3 expression level is relatively high. The Sox2 protein is especially important for the development of the eyes. This protein regulates the activity of other genes by attaching (binding) to specific regions of DNA. In addition, Cyclin-dependent kinase inhibitor p21 controls adult neural stem cell expansion by regulating Sox2 gene expression.

As can be seen from Fig. S3(a-c) in ME dataset and MEF (see Fig. S4(a-c)) dataset, the branching pattern is repeatable in multiple embedding technologies (pca, umap, t-sne) and the trace branching phenomenon can be obviously observed in the cluster graph. We took 5 different sets of variable gene subsets (80%) to define the trajectories(referring to Fig. S3(d)). When observing the visual locus after dimensionality reduction, it was found that the branch points still existed stably, ensuring the stability and elasticity of the fulcrum when information was lost. Moreover, the 5 randomly selected gene subsets ensured the statistical robustness of the branch points. For ME dataset, the cluster label is used as given in the reference (Wei et al.,2021). For the clustering and pseudo-time graph of this dataset, our result graph is similar to the result of this literature (see Fig. 3 in reference(Wei *et al*.,2021)). For MEF dataset, we find that the pseudo-time calculated by scVelo algorithm is coarse-granulated. By clustering the dataset in three different ways and coloring the labels coarse-granulated according to pseudo-time, obvious branching phenomena can be found to ensure that the identified branches are real in the data and not the product of dimensionality reduction process, and the MEF data itself has differentiation phenomenon, see reference (Luo et al.,2023).


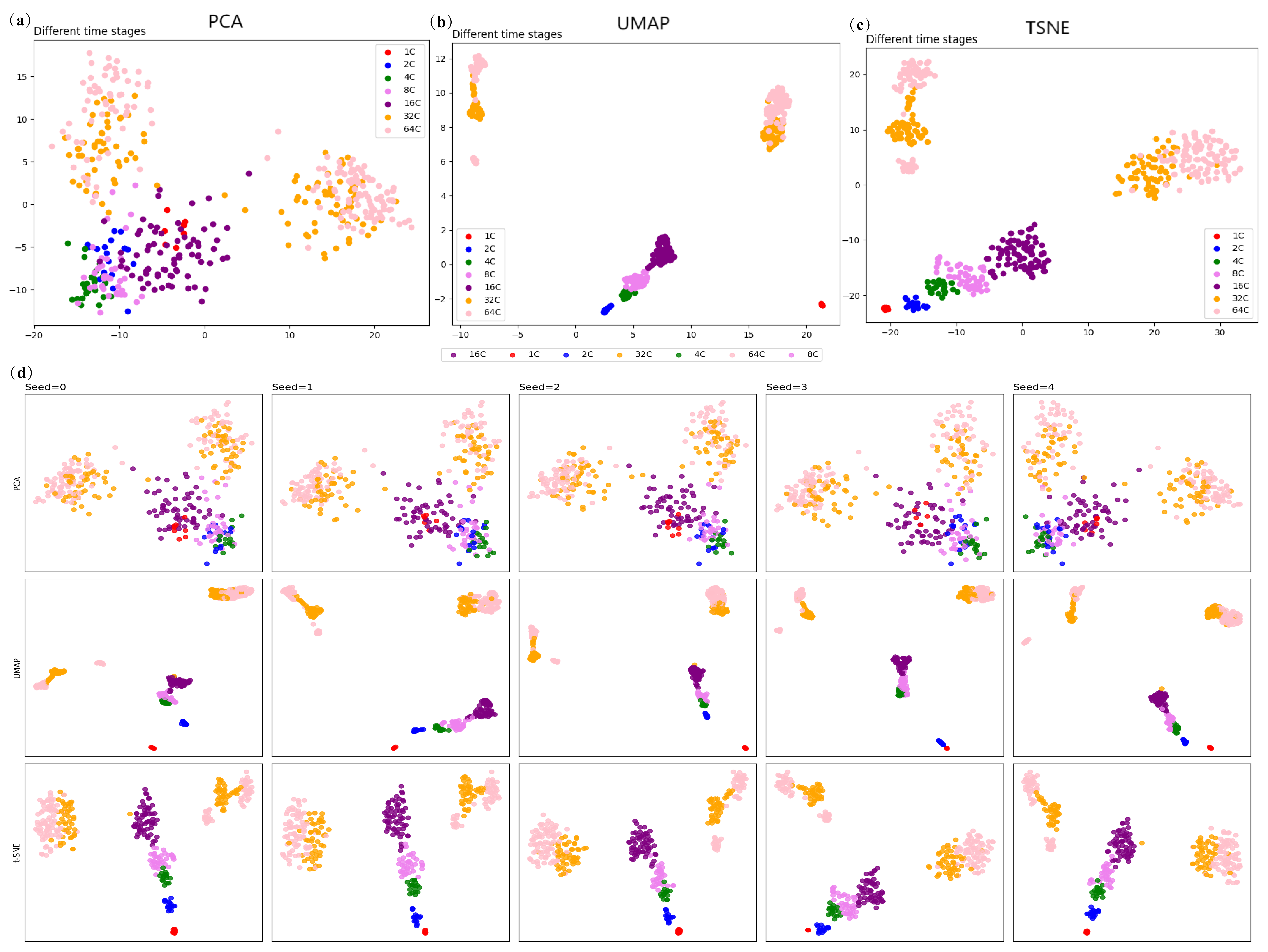


**Fig. S3** Visualization of clustering with full genes and clustering results of 5 random gene sets(80%) in mouse embryonic cells. (a) PCA clustering of mouse embryonic cells. (b) TSNE clustering of mouse embryonic cells. (c) UMAP of mouse embryonic cells. (d) Clustering results of 5 random gene sets(80%).

We observe from Fig. 5 that before the branch, there is a distinct peak (referring to Fig. 5(a)); at the branching point, there are one moderately high peak and one higher peak (Fig. 5(b)); after the branch, there are three peaks for two different branches (Fig. 5(c) and Fig. 5(d)). The numbers of peaks in branch 1 and branch 2 are relatively similar, but there is a significant difference between the peak heights, indicating that these two clusters after differentiation would have considerable differences in functions.

Although we merely analyzed the joint probability distributions of key genes Gata3 and Sox2 before and after bifurcations as well as branching points, the differences between these distributions would imply that different molecular mechanisms govern the evolutionary processes of mouse embryonic cells before and after bifurcations.

The GENIE3 is a tree-based model that yields a separate ranking of the genes as potential regulators of a target gene in the form of weights computed as sums of total variance reductions. From Fig. 6(a) we observe that with cell progression, the regulatory strength of Gata3 over Sox2 first increases slowly (i.e., with a smaller slope) before branch and then increases sharply (i.e., with a larger slope) after branch. Different from the case of Gata3 over Sox2, the regulatory strength of Sox2 over Gata3 is almost a constant before branch but increases significantly after branch, seeing to Fig. 6(b). The results shown in Fig. 6(a,b) indicate that the interaction between marker genes Gata3 and Sox2 before bifurcation is in general weak and the interaction strength is in general small. By contrast, the interaction between Gata3 and Sox2 after bifurcation becomes stronger and the interaction strength becomes more and more with the further evolution of cells. That is, the interaction strength between these two key genes significantly increases in the later stages of differentiation, and their mutual regulation shows a trend of synchronous change. In addition, the interaction strength is stronger in one branch than in the other branch.

Fig. 7(a,b) indicates that gene Gata3 promotes the expression of gene Sox2 but the latter seems not to affect the expression of the former before bifurcation, i.e., the interaction between these genes is unidirectional before bifurcation. After bifurcation, however, the interaction between gene Gata3 and gene Sox2 are bidirectional. Moreover, the former enhances the expression of the latter that also enhances the expression of the former. Thus, according to this kind of regulation relationship, we can analyze the time-evolutional dynamics of the module of these two genes after branch by establishing a chemical master equation for this module. The details are omitted here.

From Fig. 7(c,d), we observe that the Wasserstein distance between Gata3 and Sox2 is smaller before branch, indicating that the activation interval of these two genes during the cell evolving process is relatively close before branch. With cell progression, the Wasserstein distance reaches the minimum during a branching stage, suggesting that the activation interval between these two genes becomes more distant during this process. After bifurcation, however, the Wasserstein distance between the two branches becomes closer again, indicating that Gata3 and Sox2 still exhibit a relatively similar gene-activated process during different cell stags.

In eukaryotic cells, most genes are expressed in a bursty fashion, i.e., gene products (mRNAs or proteins) are produced in episodes of high transcriptional activity followed by long periods of inactivity. This bursting kinetics is usually quantified by burst size and burst frequency. Therefore, we focus on the calculation of mean burst sizes and frequencies of key genes along the reconstructed pseudo-time trajectories. By this calculation, we try to see whether there are any changes before and after branches.

Regarding bursting kinetics, we supplement the following contents for better understanding. When a gene is in an active promoter state, it is assumed to transcribe at a rate, *s* (per unit time), and the number of mRNA molecules of the gene is assumed to decay at a rate, *d* (per unit time). Subsequently, transcriptional bursting can be characterized by two parameters: the average number of synthesized mRNA molecules while the gene remains in the active state (burst size or transcriptional efficiency, ) and the frequency at which bursts occur per unit time (burst frequency, ).


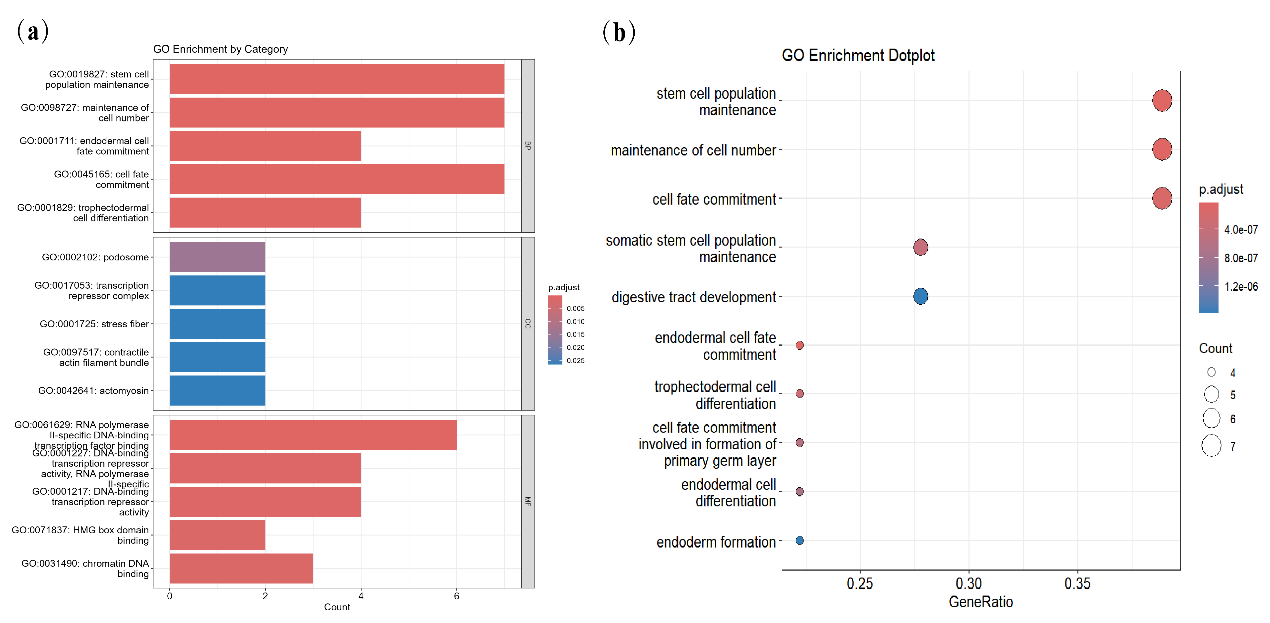


**Fig. S4** GO analysis results of marker gene in mouse embryonic cells. (a) GO enrichment by category, BP represents biological process, CC means cell component, MF stands for molecular function. The value of P.adjust is the correction value of false discovery rate in gene set enrichment analysis. (b) GO enrichment dotplot.

We performed a GO Biological Process analysis on the genes with p-values less than 0.05 in this dataset. Marker genes are mainly associated with stem cell population maintenance (GO:0019827), maintenance of cell number (GO:0098727) and cell fate commitment (GO:0045165) (Fig. S4) for biological process. There is significant enrichment in podosome for cell component (GO: 0002102), it is a dynamic structure for the interaction between cells and the extracellular matrix, mainly present in migratory cells (such as immune cells, tumor cells, etc.). Composed of actin filaments and others, it plays an important role in cell adhesion, migration, and invasion. The molecular function of RNA polymerase II-specific DNA-binding transcription factor activity (GO:0061629) plays a key role in gene expression regulation.

C3. Mouse embryonic fibroblasts

Clusters one to four are pre-branch clusters, cluster five is a cluster at the branching point, and clusters six and seven, along with clusters eight and nine, respectively, represent the two parallel post-branch clusters. Under default parameters, the clustering effects of PCA, TSNE, and UMAP are not optimal, but the cells still exhibit distinct bifurcation characteristics, refer to Fig. S5 for cell pseudo-trajectories constructed using scRNA-seq data, and Fig. S6-S11 and Table. S2-S4 for the mRNA expression patterns of key genes along the pseudo-trajectories.

The mRNA distributions of gene Mbn1 for one branch and before branch are all bimodal, the mRNA distributions of gene Pod1 for two branches and before branch are all unimodal, and the mRNA distributions of genes Ralb for two branches and before branch are all unimodal. Moreover, there is indeed a branch such that the mRNA distribution of each of these key genes for this branch is the same as that before bifurcation. The same mode of the distributions before and after branch indicates the fact that the genetic information can be still retained in the complex evolutionary process of cells. However, we point out that in spite of the same mode, the distributions are actually different since their peakness and skewness are different, referring to Fig. S6, S8 and S10.

The Wasserstein Distance between Mbnl1 and Pod1 shows no significant change before branch and at the branching point, but it increases in cluster 1 after branch, indicating that these two genes are not continuously activated in the cell evolutionary process of this branch. In contrast, Pod1 and Ralb have a large Wasserstein Distance before branch, which gradually decreases along the cell pseudo trajectories. See Fig. S29. The Wasserstein distance between key genes in the remaining two datasets is shown in Tables. S25 and S26.

**
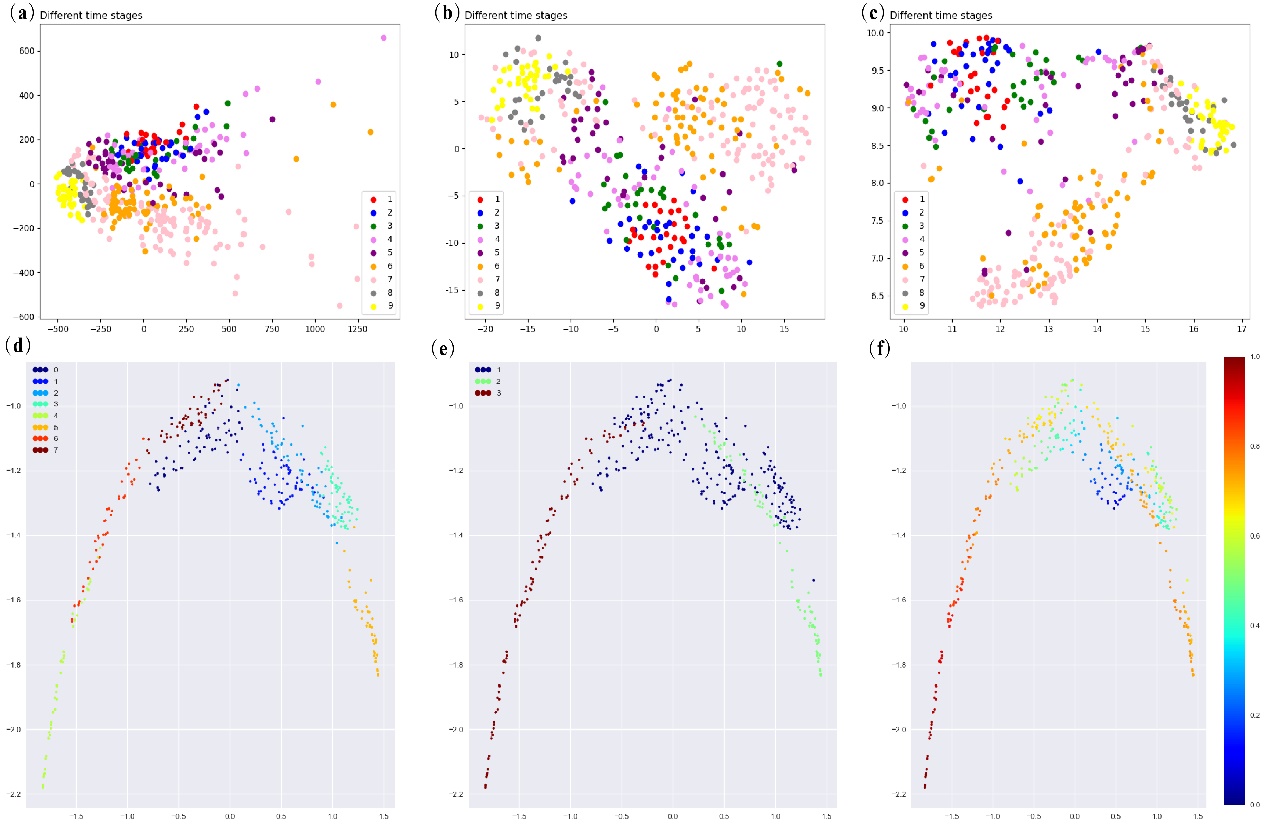
**

**Fig. S5** Visualization of clustering and branching trajectories in mouse embryonic fibroblasts cells. (a) PCA clustering of mouse embryonic fibroblasts. (b) TSNE clustering of mouse embryonic fibroblasts. (c) UMAP of mouse embryonic fibroblasts. (d) Stage clustering. (e) Branch identification. (f) Pseudo-time inference clustering visualization.

**Table S2. The inferred parameter values for the Mbnl1 gene before branch, at branching point, and after branch 1 and branch 2.**

|  | Distribution type | parameter | | | | | |
| --- | --- | --- | --- | --- | --- | --- | --- |
|  |  |  |  |  |  |
| Before branch | Bimodal mRNA distribution | 0.8124 | 1.2614 | 3.4856 | 24.0102 | 1.6297 |  |
| Branch point | Unimodal mRNA distribution |  | 0.178 | 2.232 |  |  | -1.0 |
| Branch 1 | Bimodal mRNA distribution | 0.7552 | 1.316 | 2.446 | 1.0 | 94.4437 |  |
| Branch 2 | Unimodal mRNA distribution |  | 0.781 | 1.980 |  |  | -1.0 |

**
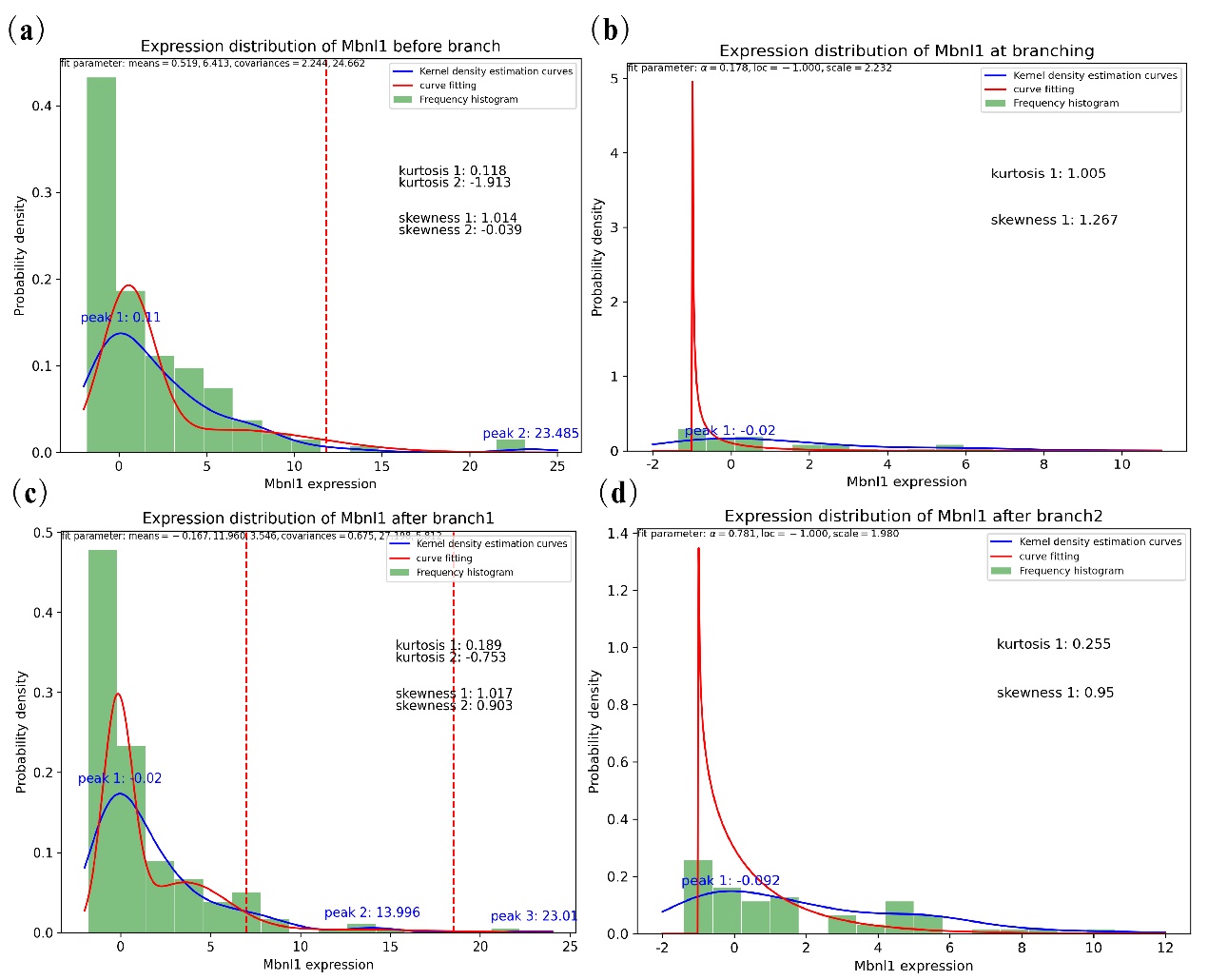
**

**Fig. S6** Statistical quantities for the Mbnl1 gene before branch, at branching point, and after branch 1 and branch 2.

**
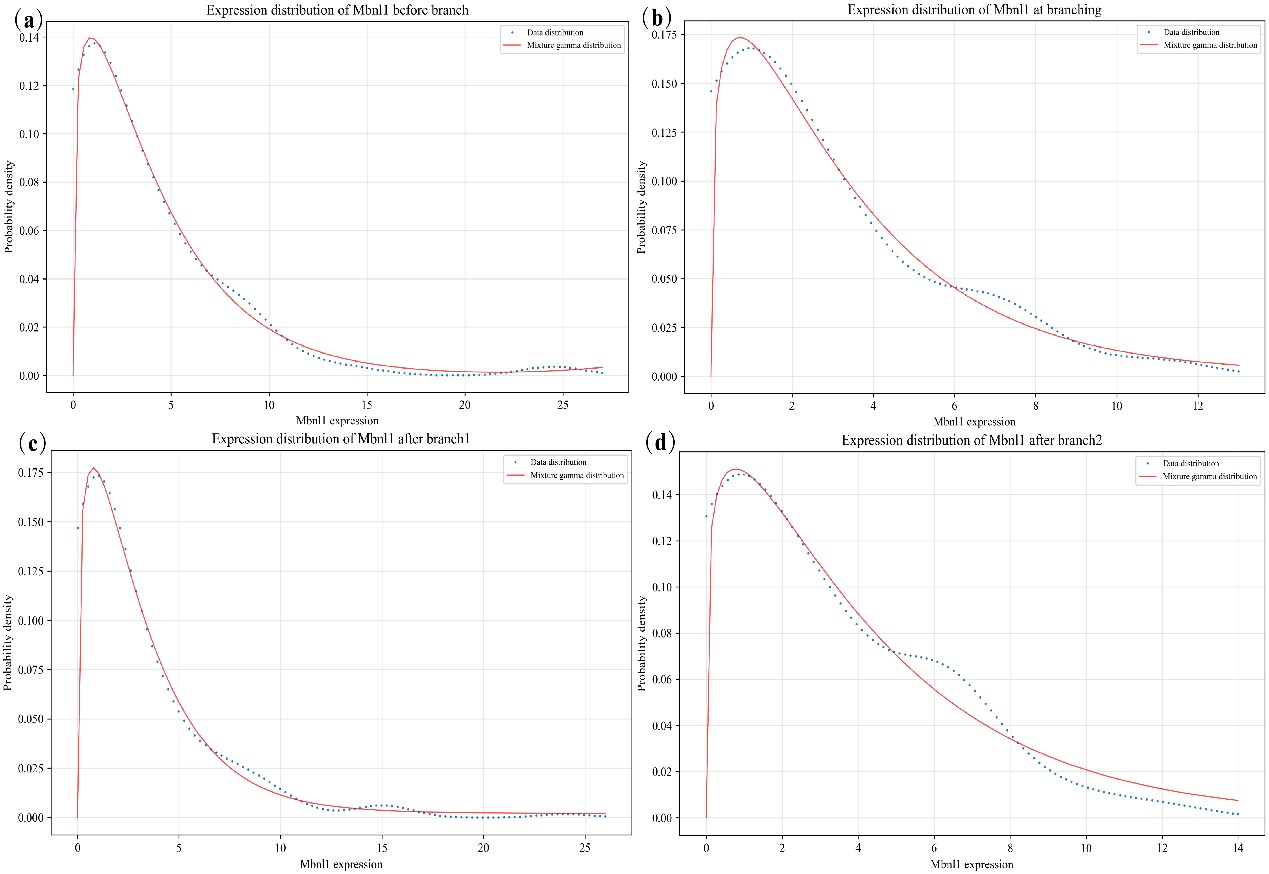
**

**Fig. S7** Different modes of the mRNA distributions for key gene Mbnl1 before and after branch as well as at branching point. The blue dashed curves represent the distributions of data points while the red curves represent the fitting results obtained using mechanic models of gene expression.

**
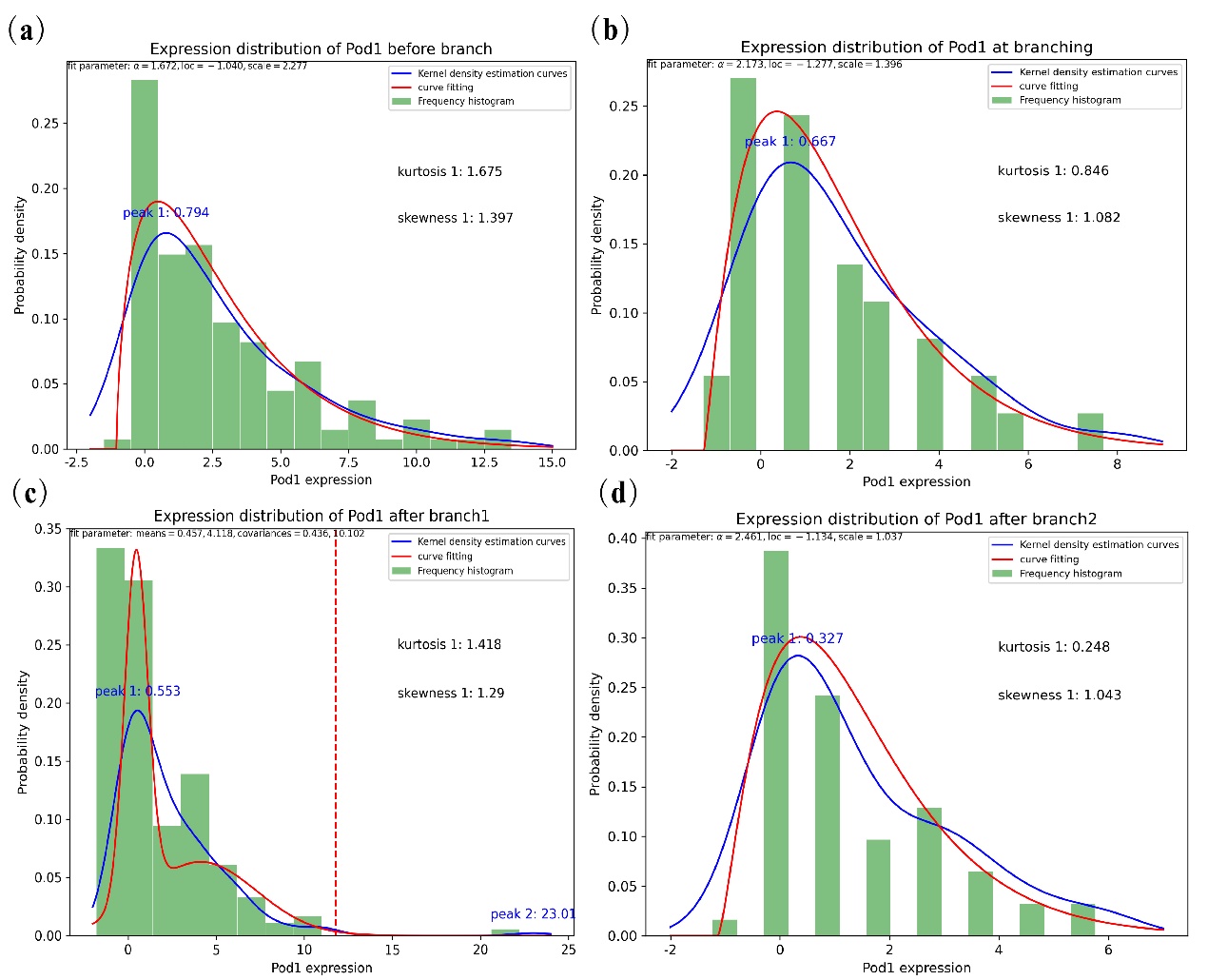
**

**Fig. S8** Statistical quantities for the Pod1 gene before branch, at branching point, and after branch 1 and branch 2.

**Table S3. The inferred parameter values for the Pod1 gene before branch, at branching point, and after branch 1 and branch 2.**

|  | Distribution type | parameter | | | | | |
| --- | --- | --- | --- | --- | --- | --- | --- |
|  |  |  |  |  |  |
| Before branch | Unimodal mRNA distribution |  | 1.672 | 2.277 |  |  | -1.04 |
| Branch point | Unimodal mRNA distribution |  | 2.173 | 1.396 |  |  | -1.277 |
| Branch 1 | Bimodal mRNA distribution | 0.9264 | 1.5926 | 2.2572 | 26.8076 | 1.339 |  |
| Branch 2 | Unimodal mRNA distribution |  | 2.461 | 1.037 |  |  | -1.134 |

**
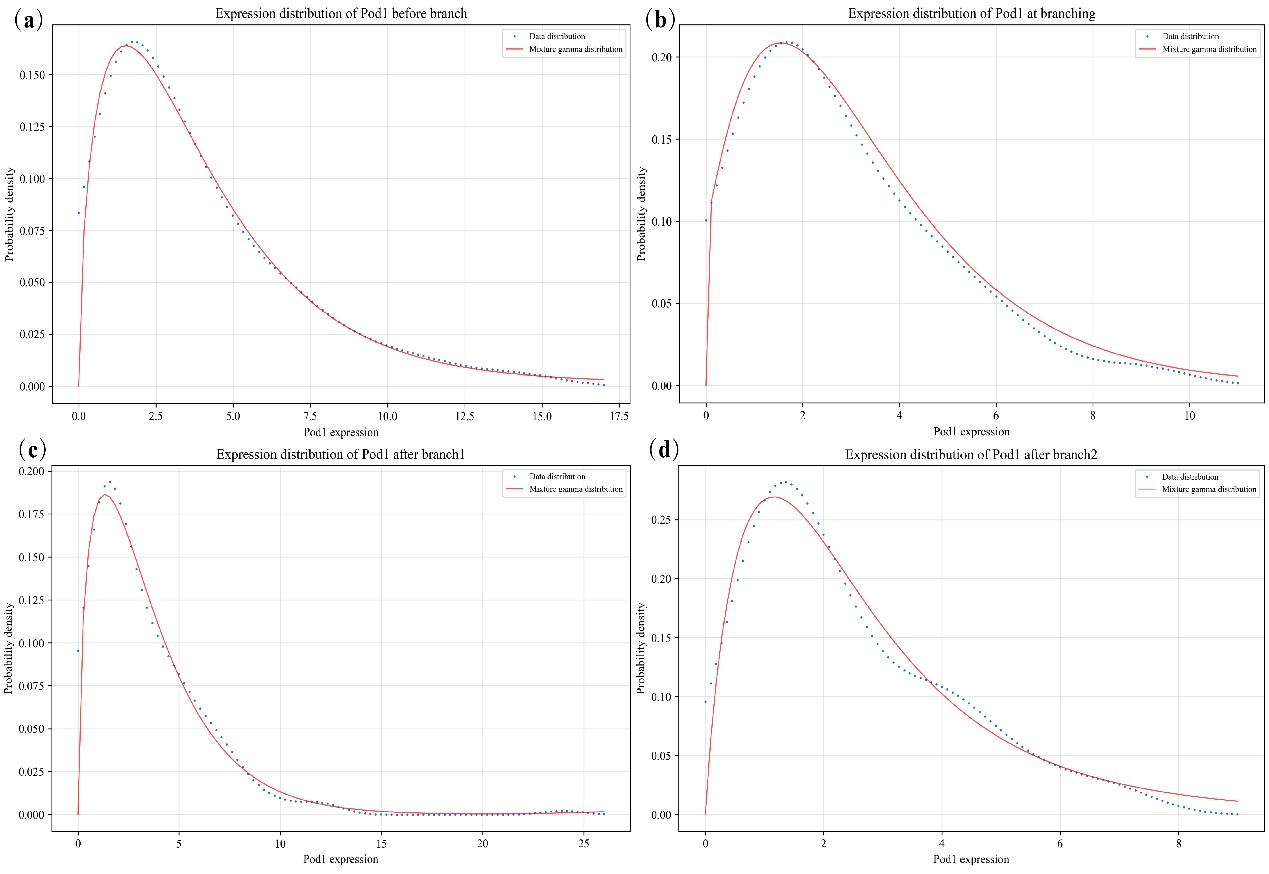
**

**Fig. S9** Different modes of the mRNA distributions for key gene Pod1 before and after branch as well as at branching point. The blue dashed curves represent the distributions of data points while the red curves represent the fitting results obtained using mechanic models of gene expression.

**
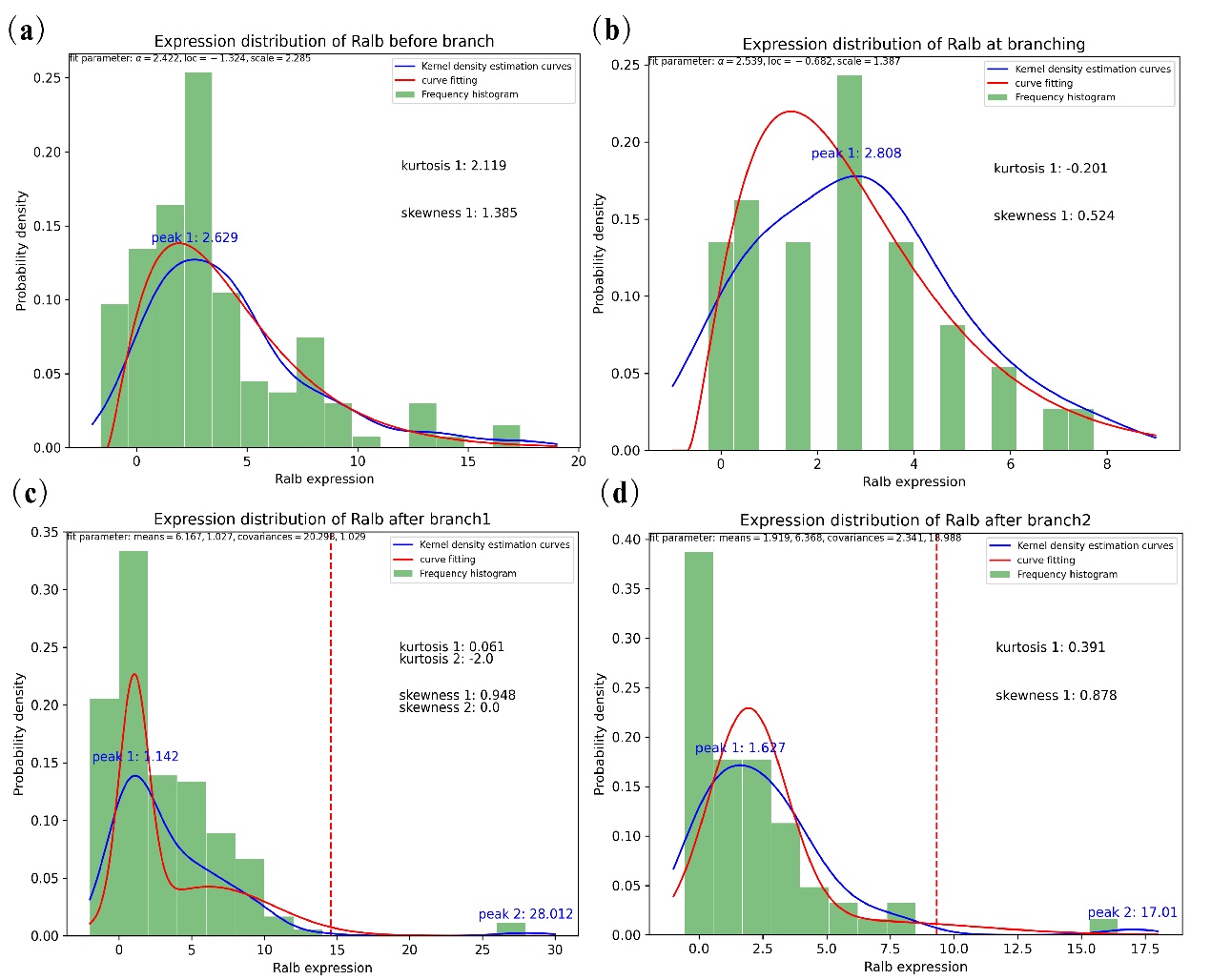
**

**Fig. S10** Statistical quantities for the Ralb gene before branch, at branching point, and after branch 1 and branch 2.

**Table S4. The inferred parameter values for the Ralb gene before branch, at branching point, and after branch 1 and branch 2.**

|  | Distribution type | parameter | | | | | |
| --- | --- | --- | --- | --- | --- | --- | --- |
|  |  |  |  |  |  |
| Before branch | Unimodal mRNA distribution |  | 2.422 | 2.285 |  |  | -1.324 |
| Branch point | Unimodal mRNA distribution |  | 2.539 | 1.387 |  |  | -0.682 |
| Branch 1 | Bimodal mRNA distribution | 0.9259 | 1.548 | 3.2449 | 28.6523 | 1.4348 |  |
| Branch 2 | Bimodal mRNA distribution | 0.3768 | 7.5322 | 0.6736 | 2.1784 | 1.8504 |  |

**
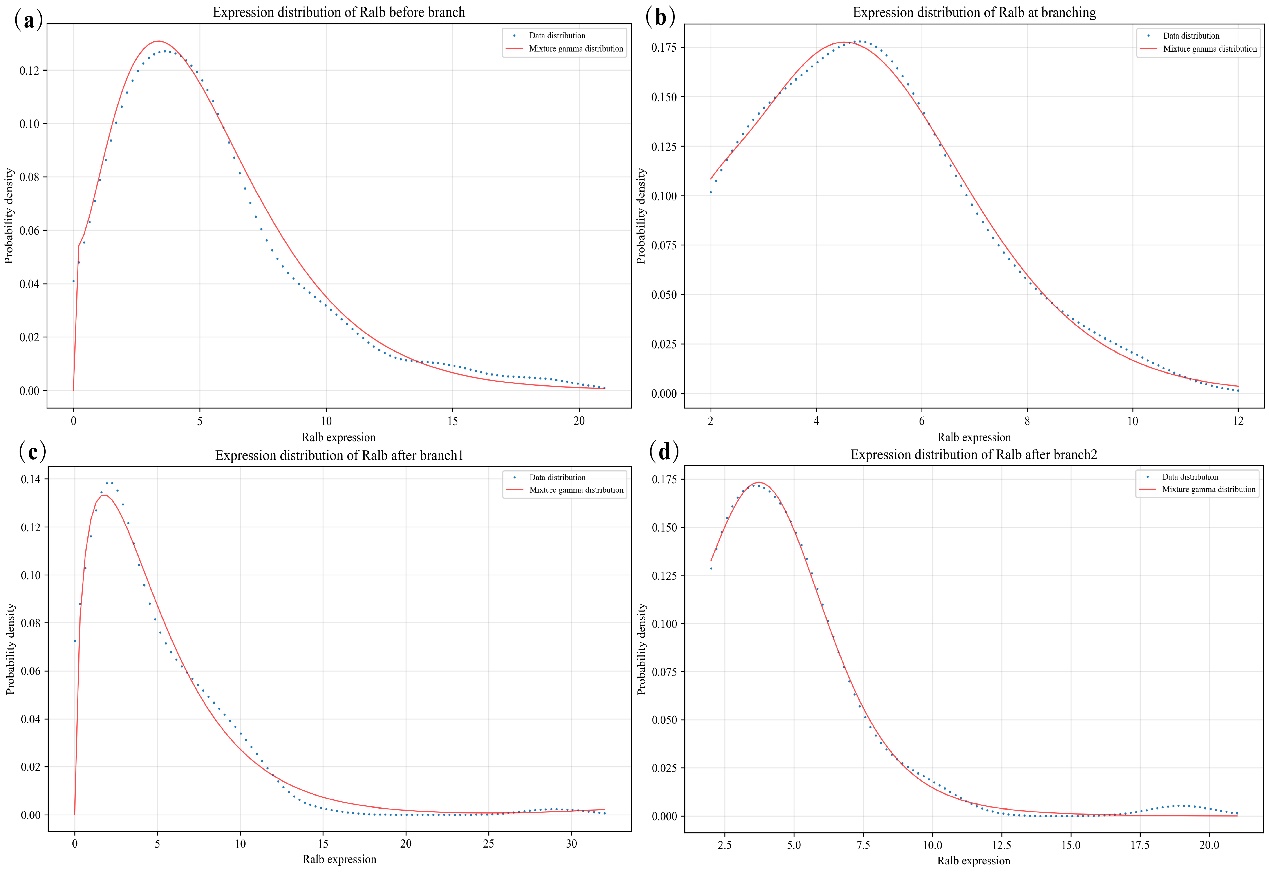
**

**Fig. S11** Different modes of the mRNA distributions for key gene Ralb before and after branch as well as at branching point. The blue dashed curves represent the distributions of data points while the red curves represent the fitting results obtained using mechanic models of gene expression.

C4. Human bone marrow

Based on pseudo-time values and prior knowledge of the data type, it is known that cells differentiate from cluster zero to cluster one, which further differentiates into clusters two, three, four, and five. The second bifurcation occurs from cluster one to cluster four, which then differentiates into clusters six and seven. Clusters five, six, seven, eight, and nine are the terminal clusters, which correspond to large pseudo-time values. Refer to Fig. S12 for cell pseudo-trajectories constructed using scRNA-seq data, and Fig. S13-S20 and Table. S5-S8 for the mRNA expression patterns of key genes along the pseudo-trajectories.


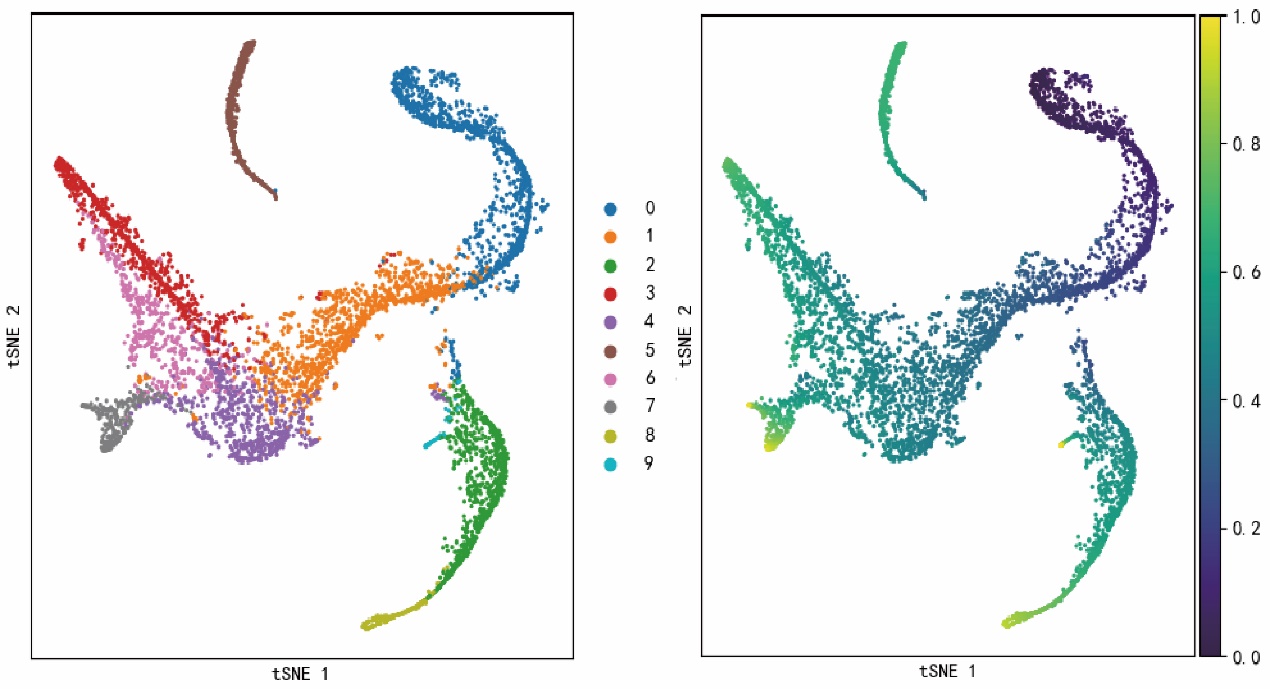


**Fig. S12** Visualization of clustering and Pseudo-time inference clustering visualization in human bone marrow dataset.


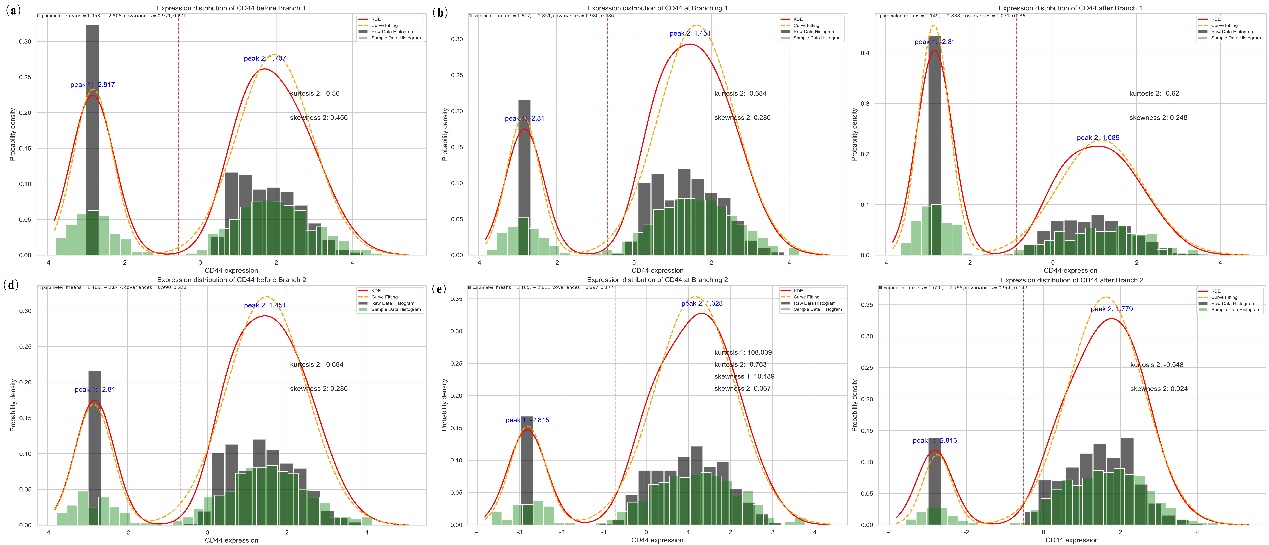


**Fig. S13** Statistical quantities for the CD44 gene before branch 1, at branching point 1, and after branch 1 and branch before branch 2, at branching point 2, after branch 2.

**Table S5. The inferred parameter values for the CD44 gene before branch 1, at branching point 1, and after branch 1 and before branch 2, at branching point 1, and after branch 2.**

|  | Distribution type | parameter | | | | |
| --- | --- | --- | --- | --- | --- | --- |
|  |  |  |  |  |
| Before branch 1 | Bimodal mRNA distribution | 0.3097 | 16.2111 | 7.7760 | 42.7084 | 6.2893 |
| Branch point 1 | Bimodal mRNA distribution | 0.2121 | 18.0700 | 9.0559 | 34.1021 | 5.3060 |
| After branch 1 | Bimodal mRNA distribution | 0.4307 | 22.6373 | 11.4480 | 33.9341 | 5.6642 |
| Before branch 2 | Bimodal mRNA distribution | 0.2235 | 18.5079 | 9.2015 | 26.4583 | 4.1372 |
| Branch point 2 | Bimodal mRNA distribution | 0.4307 | 22.6373 | 11.4480 | 33.9341 | 5.6642 |
| After branch 2 | Bimodal mRNA distribution | 0.1368 | 19.7204 | 9.8576 | 34.1461 | 5.2899 |


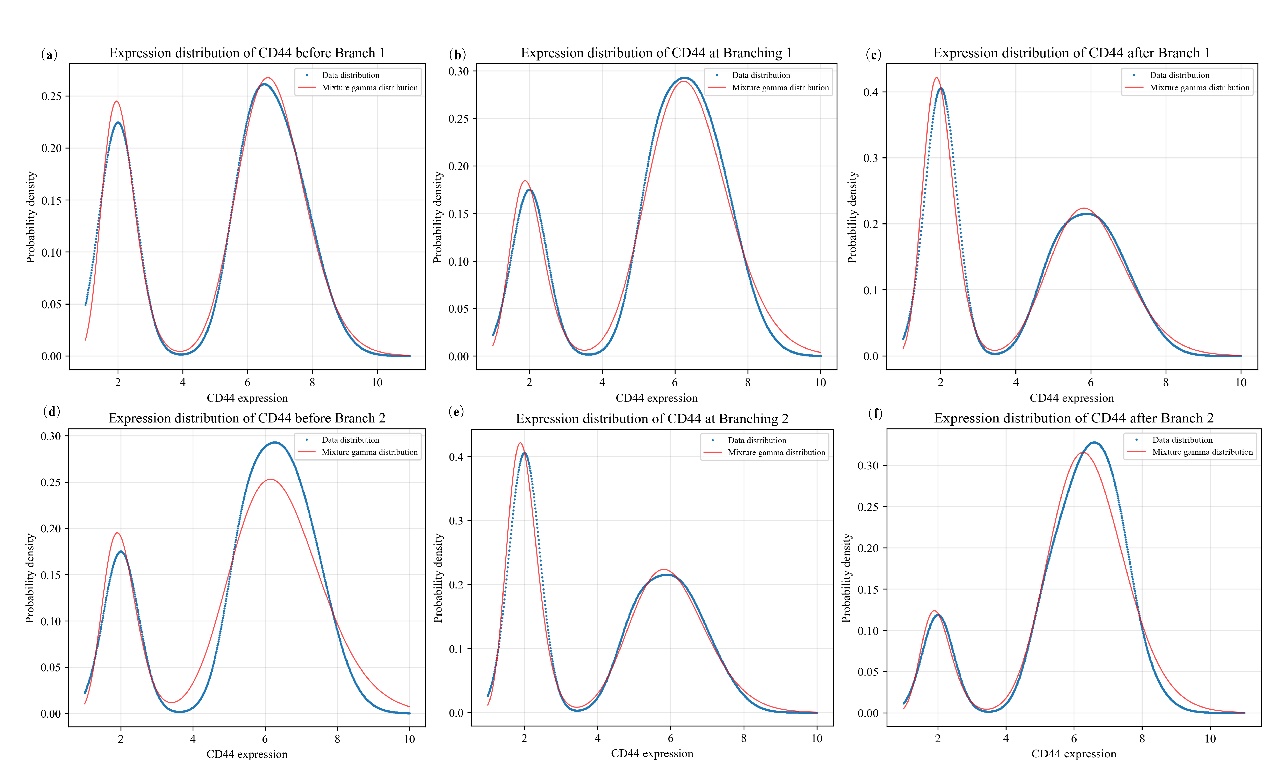


**Fig. S14** Different modes of the mRNA distributions for key gene CD44 before and after branch as well as at branching point. The blue dashed curves represent the distributions of data points while the red curves represent the fitting results obtained using mechanic models of gene expression.


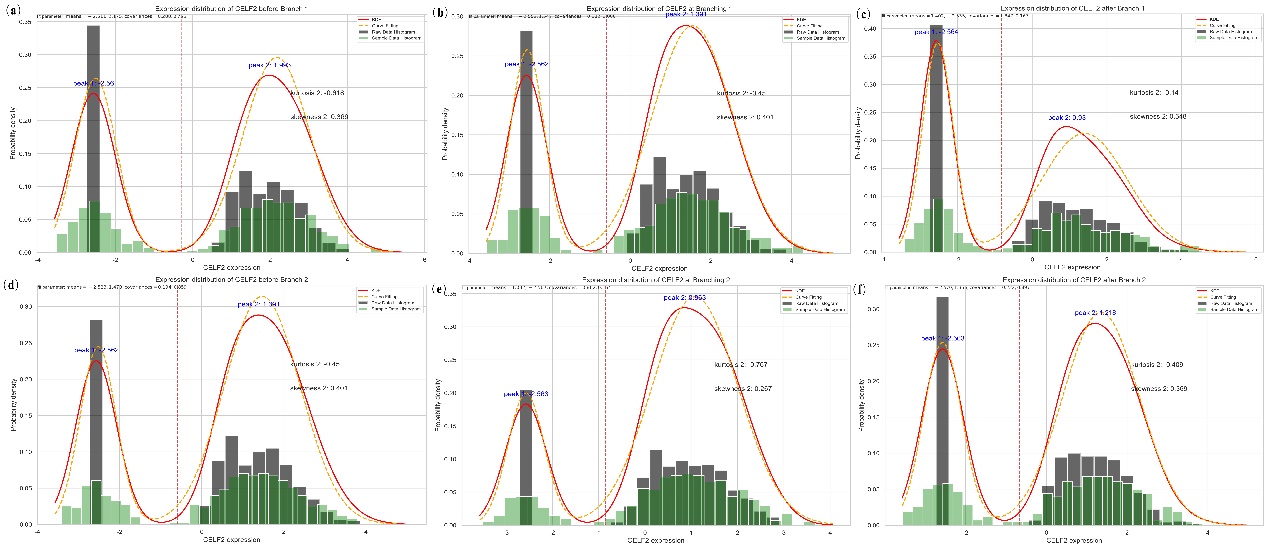


**Fig. S15** Statistical quantities for the CELF2 gene before branch 1, at branching point 1, and after branch 1 and branch before branch 2, at branching point 2, after branch 2.

**Table S6. The inferred parameter values for the CELF2 gene before branch 1, at branching point 1, and after branch 1 and before branch 2, at branching point 1, and after branch 2.**

|  | Distribution type | parameter | | | | |
| --- | --- | --- | --- | --- | --- | --- |
|  |  |  |  |  |
| Before branch 1 | Bimodal mRNA distribution | 0.6622 | 48.7593 | 7.2411 | 13.2580 | 6.5920 |
| Branch point 1 | Bimodal mRNA distribution | 0.7238 | 39.695 | 6.4558 | 16.9998 | 8.2508 |
| After branch 1 | Bimodal mRNA distribution | 0.4044 | 21.2323 | 10.6044 | 24.9344 | 4.1927 |
| Before branch 2 | Bimodal mRNA distribution | 0.2778 | 17.6067 | 8.7570 | 34.1300 | 5.5468 |
| Branch point 2 | Bimodal mRNA distribution | 0.4044 | 21.2323 | 10.6044 | 24.9344 | 4.1927 |
| After branch 2 | Bimodal mRNA distribution | 0.6882 | 38.4897 | 6.4641 | 15.7893 | 7.6394 |


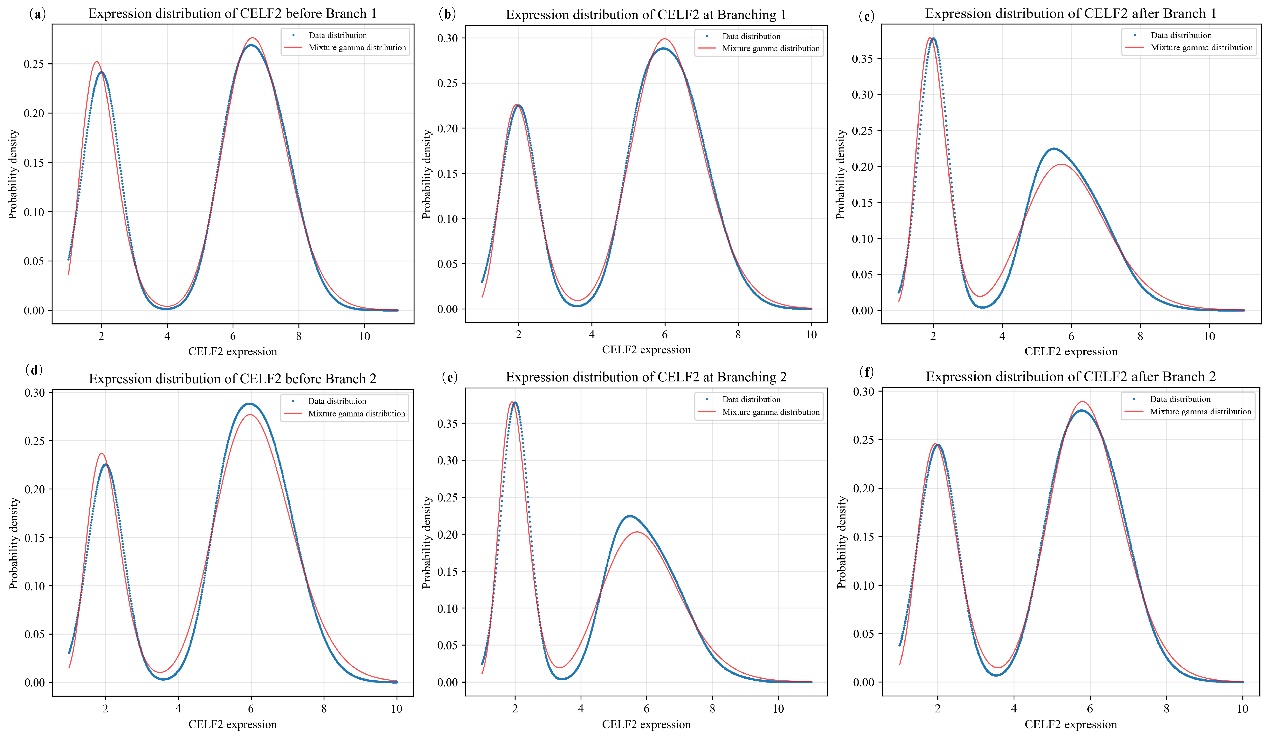


**Fig. S16** Different modes of the mRNA distributions for key gene CELF2 before and after branch as well as at branching point. The blue dashed curves represent the distributions of data points while the red curves represent the fitting results obtained using mechanic models of gene expression.


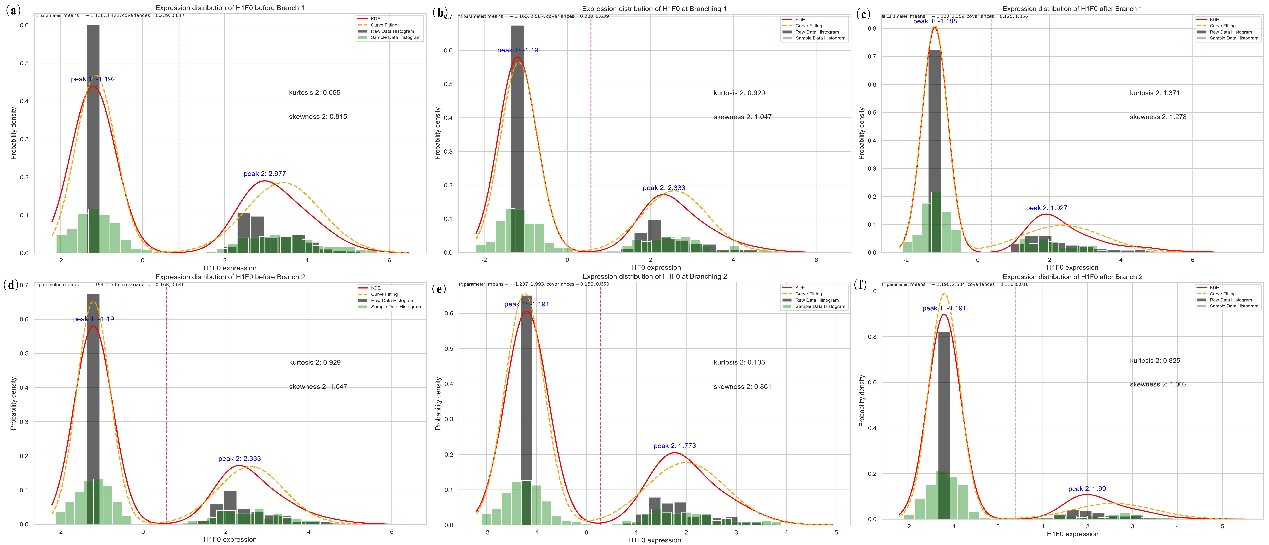


**Fig. S17** Statistical quantities for the H1F0 gene before branch 1, at branching point 1, and after branch 1 and branch before branch 2, at branching point 2, after branch 2.

**Table S7. The inferred parameter values for the H1F0 gene before branch 1, at branching point 1, and after branch 1 and before branch 2, at branching point 1, and after branch 2.**

|  | Distribution type | parameter | | | | |
| --- | --- | --- | --- | --- | --- | --- |
|  |  |  |  |  |
| Before branch 1 | Bimodal mRNA distribution | 0.4053 | 54.4009 | 8.4818 | 15.6535 | 8.1766 |
| Branch point 1 | Bimodal mRNA distribution | 0.6783 | 20.0605 | 9.9825 | 23.9011 | 4.0988 |
| After branch 1 | Bimodal mRNA distribution | 0.7270 | 23.3733 | 11.7181 | 22.5725 | 3.9571 |
| Before branch 2 | Bimodal mRNA distribution | 0.6783 | 20.0605 | 9.9825 | 23.9011 | 4.0988 |
| Branch point 2 | Bimodal mRNA distribution | 0.7270 | 23.3733 | 11.7181 | 22.5725 | 3.9571 |
| After branch 2 | Bimodal mRNA distribution | 0.8294 | 23.2577 | 11.6576 | 22.4327 | 4.0689 |


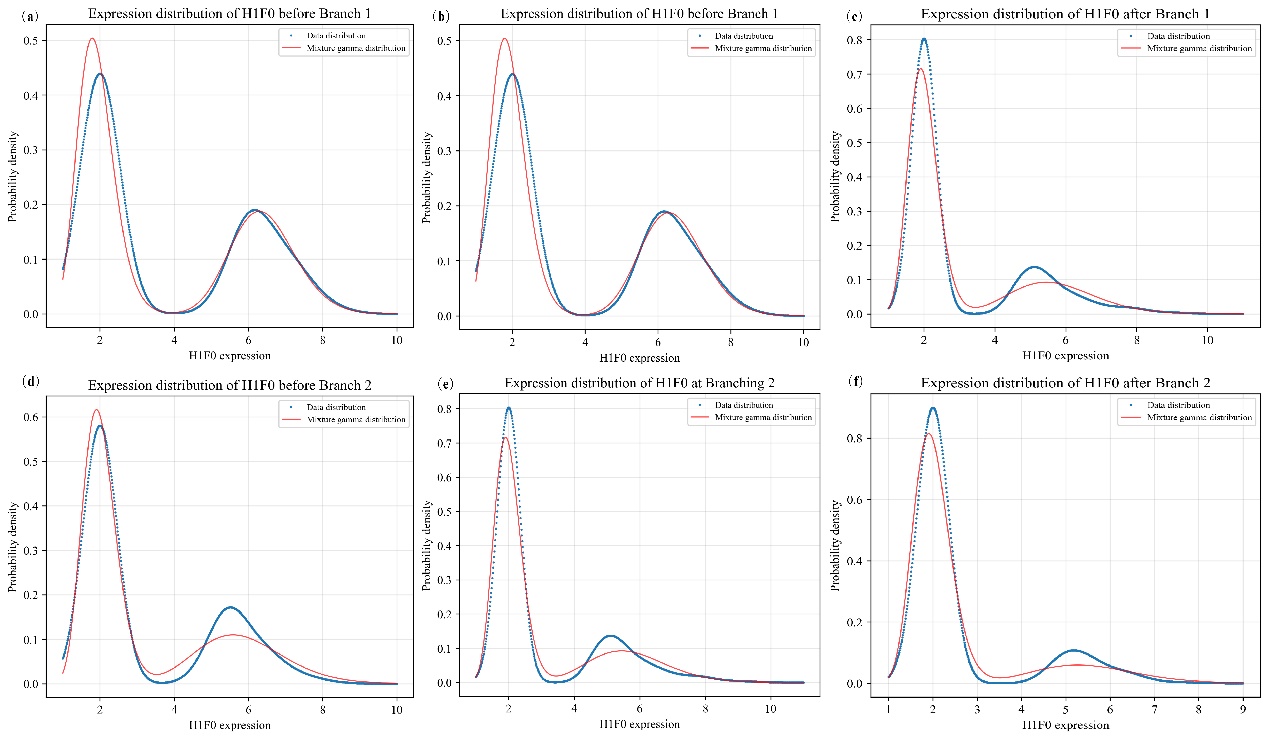


**Fig. S18** Different modes of the mRNA distributions for key gene H1F0 before and after branch as well as at branching point. The blue dashed curves represent the distributions of data points while the red curves represent the fitting results obtained using mechanic models of gene expression.

**
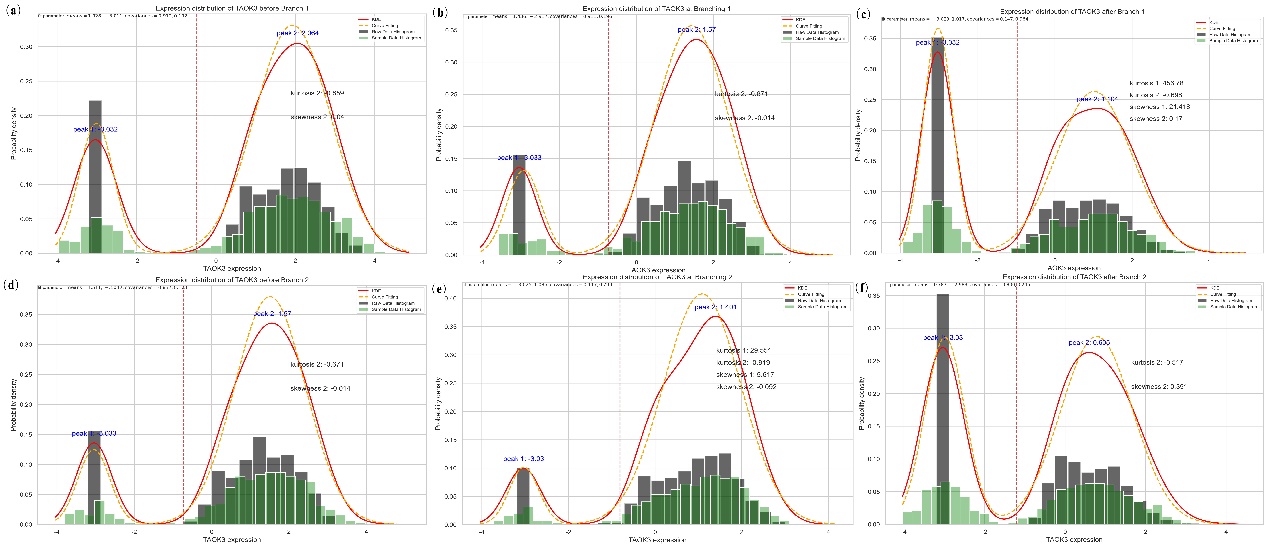
**

**Fig. S19** Statistical quantities for the TAOK3 gene before branch 1, at branching point 1, and after branch 1 and branch before branch 2, at branching point 2, after branch 2.

**Table S8. The inferred parameter values for the TAOK3 gene before branch 1, at branching point 1, and after branch 1 and before branch 2, at branching point 1, and after branch 2.**

|  | Distribution type | parameter | | | | |
| --- | --- | --- | --- | --- | --- | --- |
|  |  |  |  |  |
| Before branch 1 | Bimodal mRNA distribution | 0.2181 | 15.7200 | 7.7539 | 34.2096 | 4.8625 |
| Branch point 1 | Bimodal mRNA distribution | 0.1554 | 20.3670 | 10.1224 | 34.2496 | 5.2514 |
| After branch 1 | Bimodal mRNA distribution | 0.3496 | 22.5168 | 11.3067 | 34.0011 | 5.5855 |
| Before branch 2 | Bimodal mRNA distribution | 0.1554 | 20.3670 | 10.1224 | 34.2496 | 5.2514 |
| Branch point 2 | Bimodal mRNA distribution | 0.3456 | 22.6956 | 11.0988 | 33.4056 | 5.4644 |
| After branch 2 | Bimodal mRNA distribution | 0.3477 | 16.1990 | 7.9937 | 34.1151 | 5.8239 |


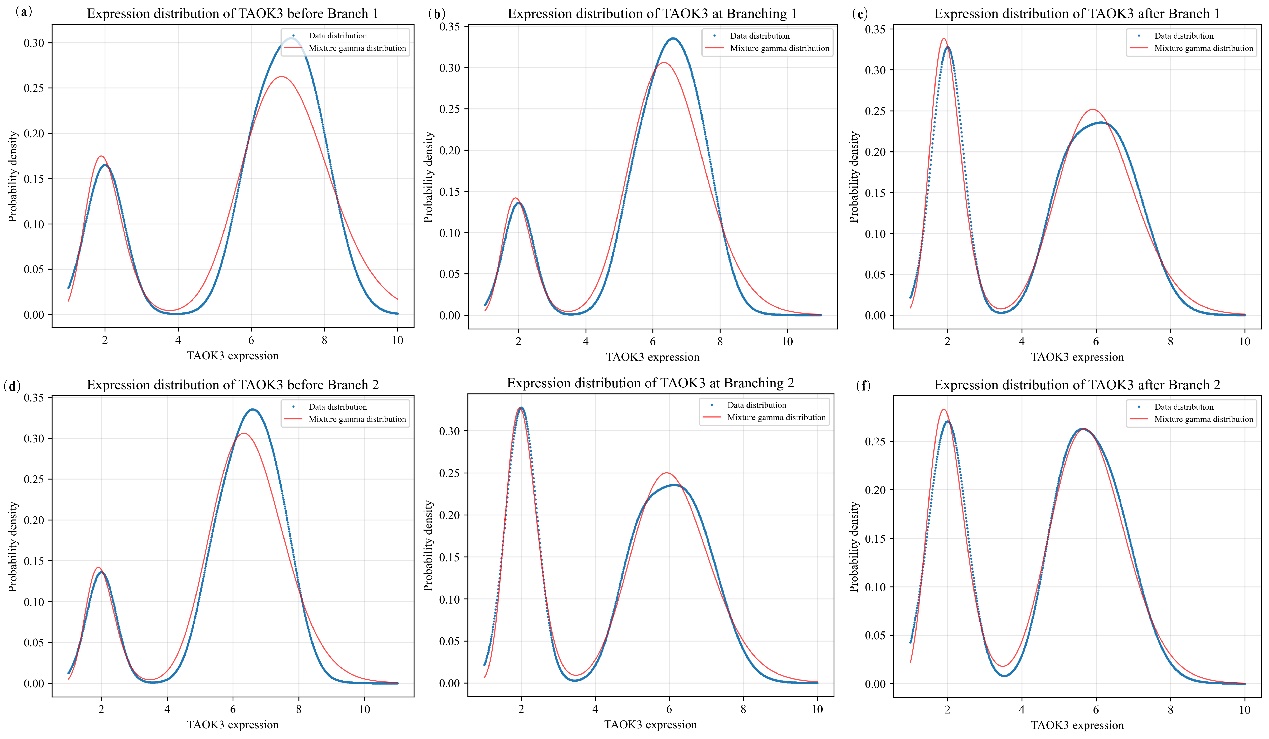


**Fig. S20** Different modes of the mRNA distributions for key gene TAOK3 before and after branch as well as at branching point. The blue dashed curves represent the distributions of data points while the red curves represent the fitting results obtained using mechanic models of gene expression.

C5. Intestine organoid

Note that TA cells are an aggregation cluster; Enteroendocrine, Enteroendocrine progenitor, Goblet cells, Paneth cells and Stem cells are a kind of pre-aggregation cluster, whereas Enteroendocrine progenitor and Tuft cells are another kind of pre-aggregation cluster; and Enterocytes is the unique post-aggregation cluster. Refer to Fig. S21 for cell pseudo-trajectories constructed using scRNA-seq data, and Fig. S22-S28 and Table. S9-S22for the mRNA expression patterns of key genes along the pseudo-trajectories.


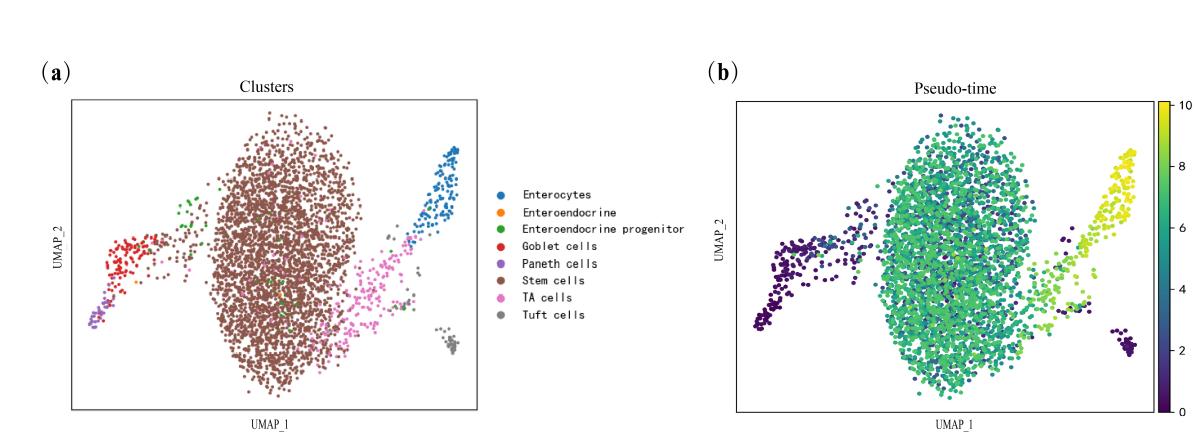


**Fig. S21** Visualization of clustering and Pseudo-time inference clustering visualization in intestinal organoid dataset.

**Table S9. Statistical quantities for the Atp2a3 gene before polymerization 1 and polymerization 2, at polymerization point, and after polymerization.**

|  | Peak position | kurtosis | skewness |
| --- | --- | --- | --- |
| Pre-polymerization cluster 1 | -0.005 |  |  |
| Pre-polymerization cluster 2 | 0.041 | 3.837 | 2.057 |
| Polymeric cluster | -0.001 |  |  |
| Polymerized cluster | -0.001 |  |  |

**Table S10. The inferred parameter values for the Atp2a3 gene before polymerization 1 and polymerization 2, at polymerization point, and after polymerization.**

|  | Distribution type | parameter | | |
| --- | --- | --- | --- | --- |
|  |  |  |
| Pre-polymerization cluster 1 | Unimodal mRNA distribution | 5876.3859 | 2366.0798 | 1.5166 |
| Pre-polymerization cluster 2 | Unimodal mRNA distribution | 2.0007 | 1.0043 | 2.8594 |
| Polymeric cluster | Unimodal mRNA distribution | 12338.4374 | 4953.2721 | 1.5091 |
| Polymerized cluster | Unimodal mRNA distribution | 4800.8117 | 1453.0475 | 0.6964 |

**
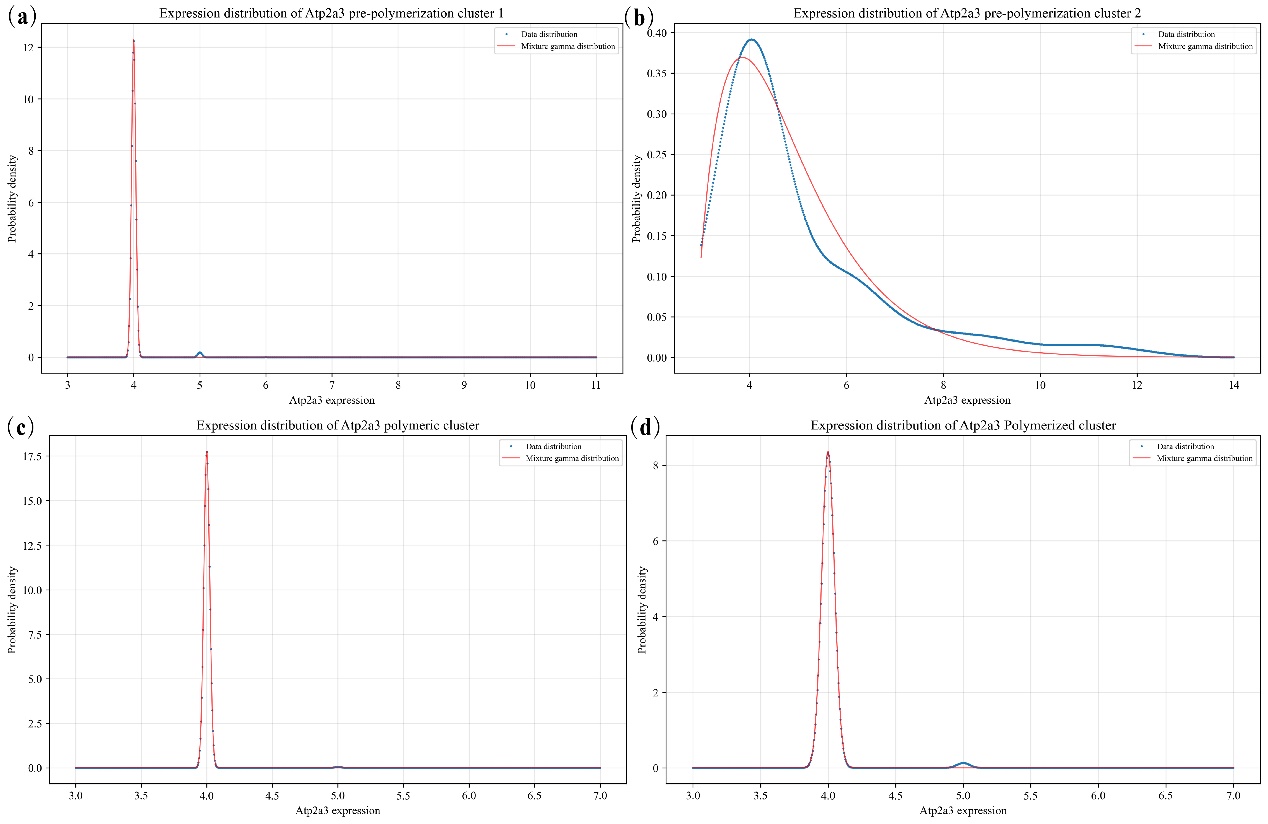
**

**Fig. S22** Different modes of the mRNA distributions for key gene Atp2a3 before and after polymerization as well as at polymerization point. The blue dashed curves represent the distributions of data points while the red curves represent the fitting results obtained using mechanic models of gene expression.

**Table S11. Statistical quantities for the Clu gene before polymerization 1 and polymerization 2, at polymerization point, and after polymerization.**

|  | Peak position | kurtosis | skewness |
| --- | --- | --- | --- |
| Pre-polymerization cluster 1 | 0.019 |  |  |
| Pre-polymerization cluster 2 | 0.083 | 82.643 | 8.947 |
| Polymeric cluster | 0.003 |  |  |
| Polymerized cluster | 0.043 | 20.188 | 4.081 |

**Table S12. The inferred parameter values for the Clu gene before polymerization 1 and polymerization 2, at polymerization point, and after polymerization.**

|  | Distribution type | parameter | | |
| --- | --- | --- | --- | --- |
|  |  |  |
| Pre-polymerization cluster 1 | Unimodal mRNA distribution | 4.5336 | 4.0261 | 3.0744 |
| Pre-polymerization cluster 2 | Unimodal mRNA distribution | 3855.5196 | 22.7441 | -165.4806 |
| Polymeric cluster | Unimodal mRNA distribution | 8.2927 | 6.5895 | 2.8567 |
| Polymerized cluster | Unimodal mRNA distribution | 5.5074 | 3.0307 | 2.5337 |

**
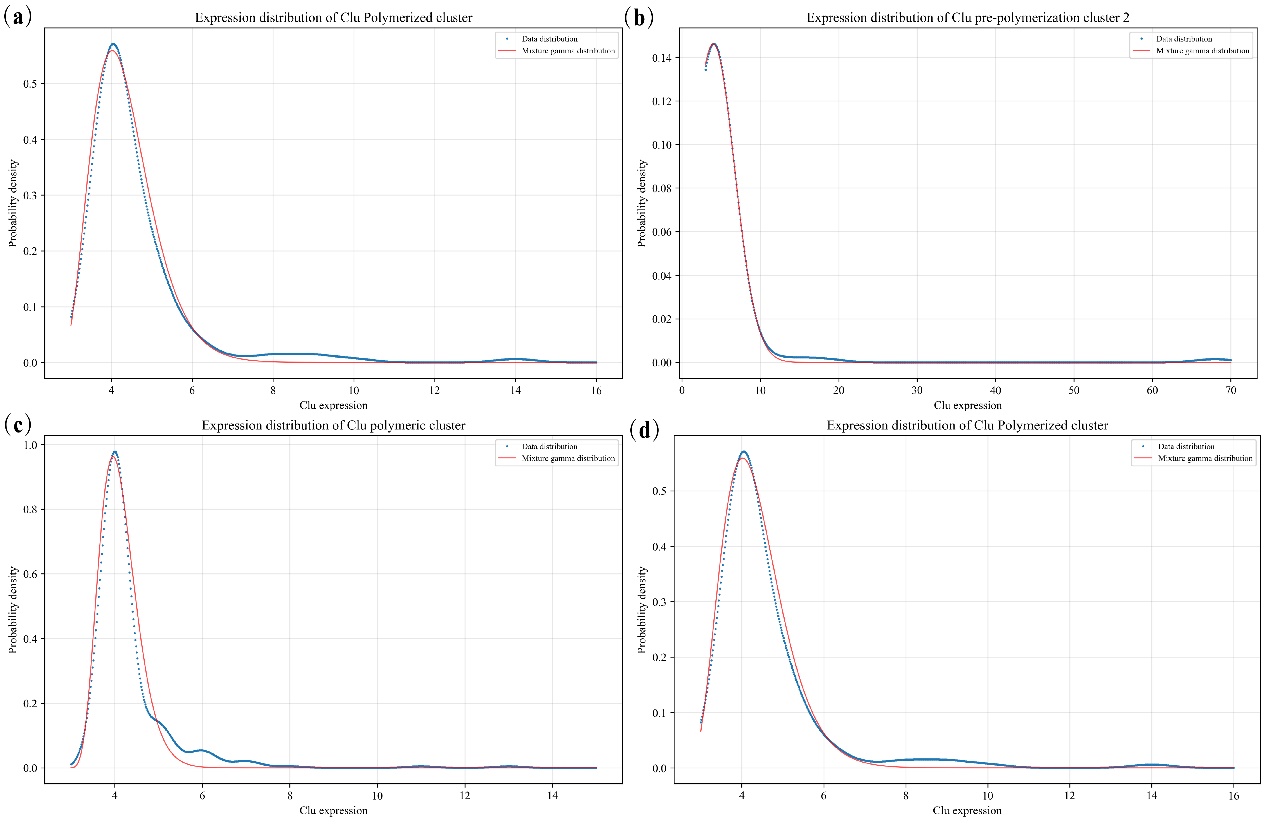
**

**Fig. S23** Different modes of the mRNA distributions for key gene Clu before and after polymerization as well as at polymerization point. The blue dashed curves represent the distributions of data points while the red curves represent the fitting results obtained using mechanic models of gene expression.

**Table S13. Statistical quantities for the Edn1 gene before polymerization 1 and polymerization 2, at polymerization point, and after polymerization.**

|  | Peak position | kurtosis | skewness |
| --- | --- | --- | --- |
| Pre-polymerization cluster 1 | 0.006 |  |  |
| Pre-polymerization cluster 2 | -0.005 | 61.845 | 7.616 |
| Polymeric cluster | 0.009 | 109.022 | 9.705 |
| Polymerized cluster | 0.003 |  |  |

**Table S14. The inferred parameter values for the Edn1 gene before polymerization 1 and polymerization 2, at polymerization point, and after polymerization.**

|  | Distribution type | parameter | | |
| --- | --- | --- | --- | --- |
|  |  |  |
| Pre-polymerization cluster 1 | Unimodal mRNA distribution | 300.2292 | 74.9539 | 0.0021 |
| Pre-polymerization cluster 2 | Unimodal mRNA distribution | 2402.3494 | 108.6915 | -18.095 |
| Polymeric cluster | Unimodal mRNA distribution | 350.8701 | 55.7226 | -2.284 |
| Polymerized cluster | Unimodal mRNA distribution | 2337.4154 | 219.2878 | -6.6565 |


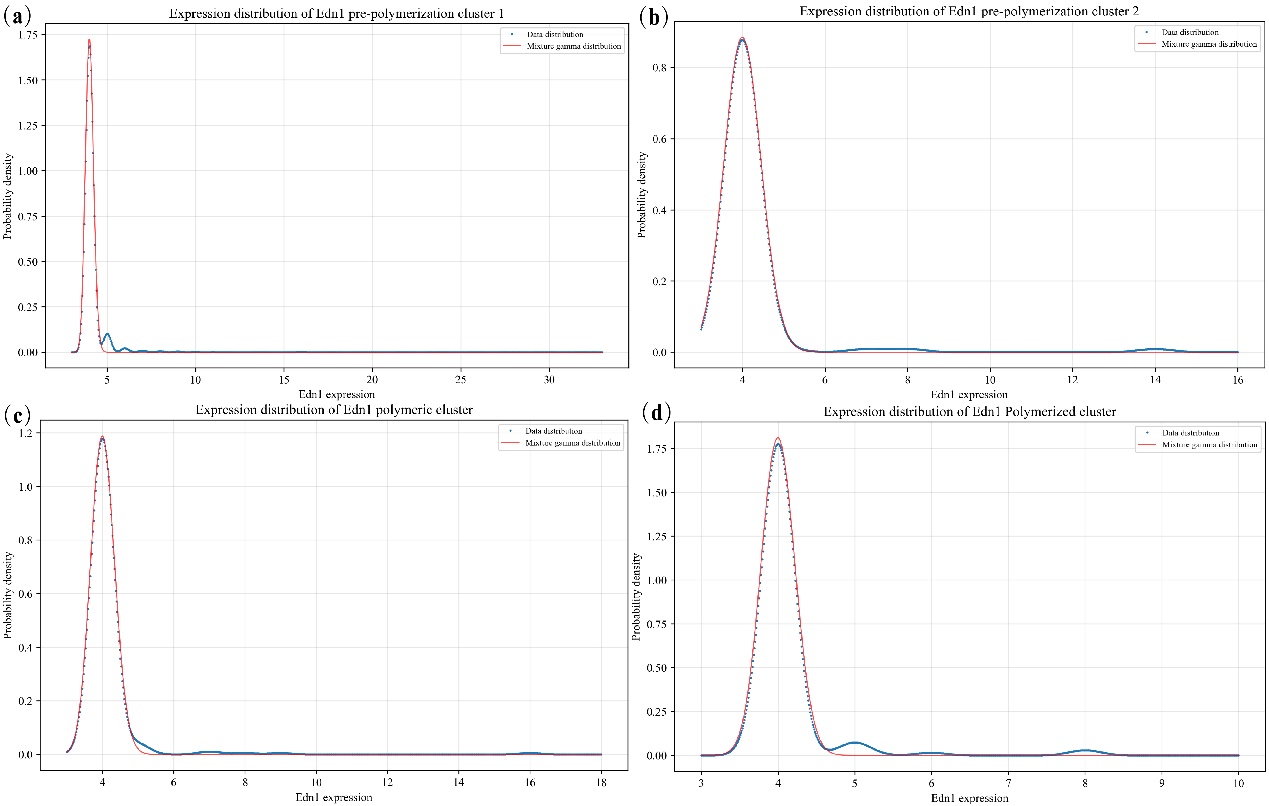


**Fig. S24** Different modes of the mRNA distributions for key gene Edn1 before and after polymerization as well as at polymerization point. The blue dashed curves represent the distributions of data points while the red curves represent the fitting results obtained using mechanic models of gene expression.

**Table S15. Statistical quantities for the Gsta4 gene before polymerization 1 and polymerization 2, at polymerization point, and after polymerization.**

|  | Peak position | kurtosis | skewness |
| --- | --- | --- | --- |
| Pre-polymerization cluster 1 | 0.037 | 130.82 | 10.101 |
| Pre-polymerization cluster 2 | 0.062 | 28.936 | 5.094 |
| Polymeric cluster | 0.63 | 16.686 | 3.275 |
| Polymerized cluster | 5.0 | 3.939 | 1.922 |

**Table S16. The inferred parameter values for the Gsta4 gene before polymerization 1 and polymerization 2, at polymerization point, and after polymerization.**

|  | Distribution type | parameter | | |
| --- | --- | --- | --- | --- |
|  |  |  |
| Pre-polymerization cluster 1 | Unimodal mRNA distribution | 15.5707 | 4.7497 | 0.9701 |
| Pre-polymerization cluster 2 | Unimodal mRNA distribution | 2932.8522 | 51.3486 | -53.0658 |
| Polymeric cluster | Unimodal mRNA distribution | 3.2182 | 0.5362 | -0.1491 |
| Polymerized cluster | Unimodal mRNA distribution | 3.7053 | 0.192 | -5.8868 |


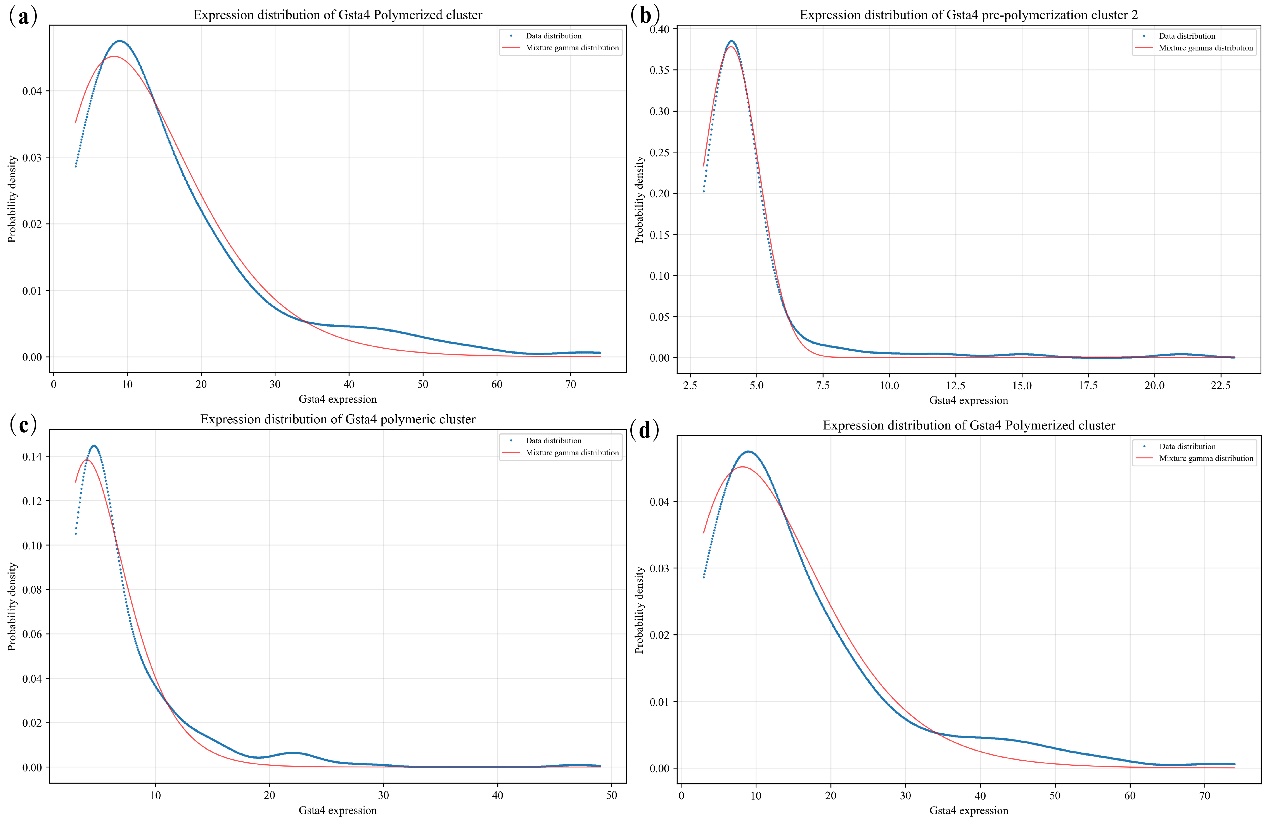


**Fig. S25** Different modes of the mRNA distributions for key gene Gsta4 before and after polymerization as well as at polymerization point. The blue dashed curves represent the distributions of data points while the red curves represent the fitting results obtained using mechanic models of gene expression.

**Table S17. Statistical quantities for the H1f0 gene before polymerization 1 and polymerization 2, at polymerization point, and after polymerization.**

|  | Peak position | kurtosis | skewness |
| --- | --- | --- | --- |
| Pre-polymerization cluster 1 | 0.083 | 9.8 | 2.497 |
| Pre-polymerization cluster 2 | 0.283 | 4.371 | 1.91 |
| Polymeric cluster | 0.508 | 8.118 | 2.553 |
| Polymerized cluster | 0.499 | 7.633 | 2.219 |

**Table S18. The inferred parameter values for the H1f0 gene before polymerization 1 and polymerization 2, at polymerization point, and after polymerization.**

|  | Distribution type | parameter | | |
| --- | --- | --- | --- | --- |
|  |  |  |
| Pre-polymerization cluster 1 | Unimodal mRNA distribution | 1.5985 | 0.6919 | 3.2664 |
| Pre-polymerization cluster 2 | Unimodal mRNA distribution | 2.0416 | 0.8262 | 2.8655 |
| Polymeric cluster | Unimodal mRNA distribution | 2.3019 | 0.6628 | 2.4069 |
| Polymerized cluster | Unimodal mRNA distribution | 3.1401 | 0.8491 | 2.187 |


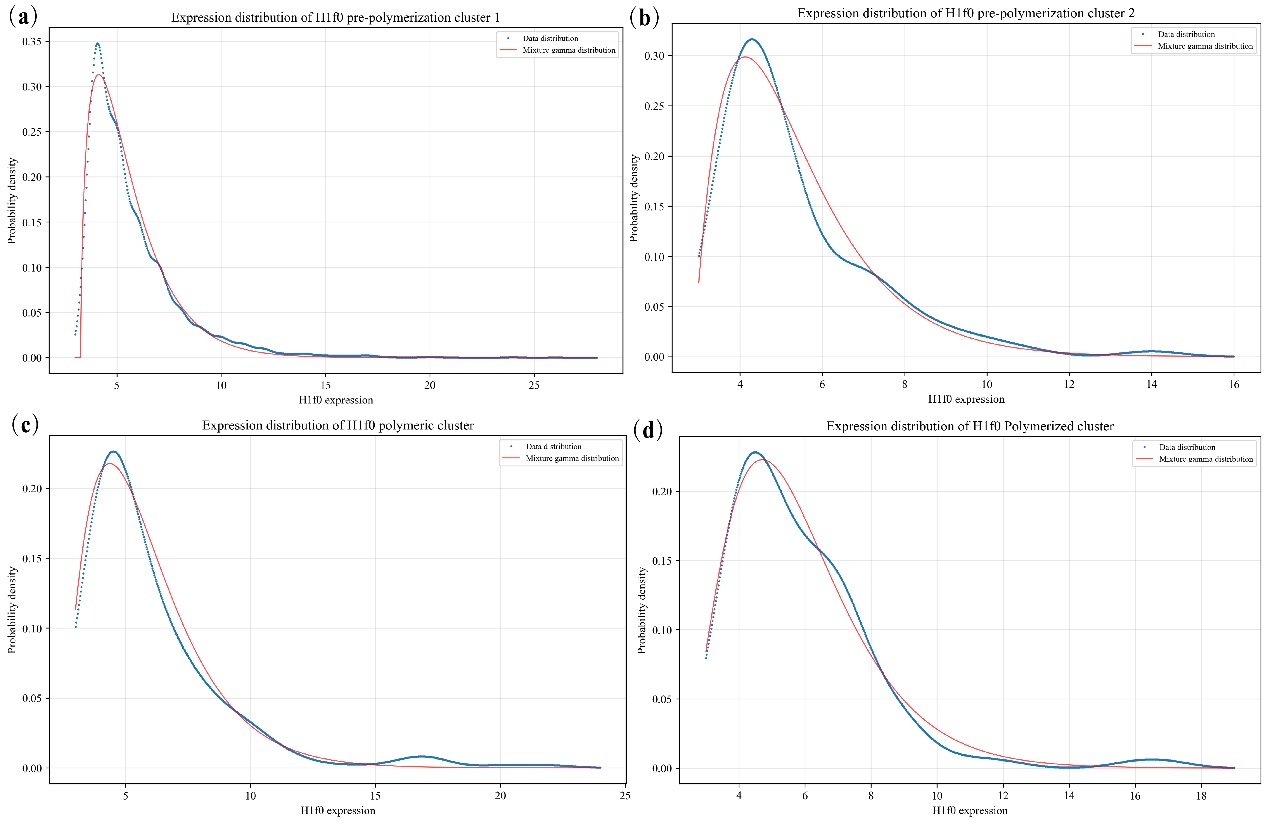


**Fig. S26** Different modes of the mRNA distributions for key geneH1f0 before and after polymerization as well as at polymerization point. The blue dashed curves represent the distributions of data points while the red curves represent the fitting results obtained using mechanic models of gene expression.

**Table S19. Statistical quantities for the Muc13 gene before polymerization 1 and polymerization 2, at polymerization point, and after polymerization.**

|  | Peak position | kurtosis | skewness |
| --- | --- | --- | --- |
| Pre-polymerization cluster 1 | 0.069 | 32.169 | 4.274 |
| Pre-polymerization cluster 2 | 0.589 | 1.328 | 1.252 |
| Polymeric cluster | 0.432 | 4.264 | 1.941 |
| Polymerized cluster | 3.39 | 4.184 | 1.686 |

**Table S20. The inferred parameter values for the Muc13 gene before polymerization 1 and polymerization 2, at polymerization point, and after polymerization.**

|  | Distribution type | parameter | | |
| --- | --- | --- | --- | --- |
|  |  |  |
| Pre-polymerization cluster 1 | Unimodal mRNA distribution | 1.8563 | 0.9878 | 3.2122 |
| Pre-polymerization cluster 2 | Unimodal mRNA distribution | 2.3877 | 0.7052 | 2.661 |
| Polymeric cluster | Unimodal mRNA distribution | 2.2348 | 0.7132 | 2.6808 |
| Polymerized cluster | Unimodal mRNA distribution | 2.4181 | 0.2671 | 1.6263 |


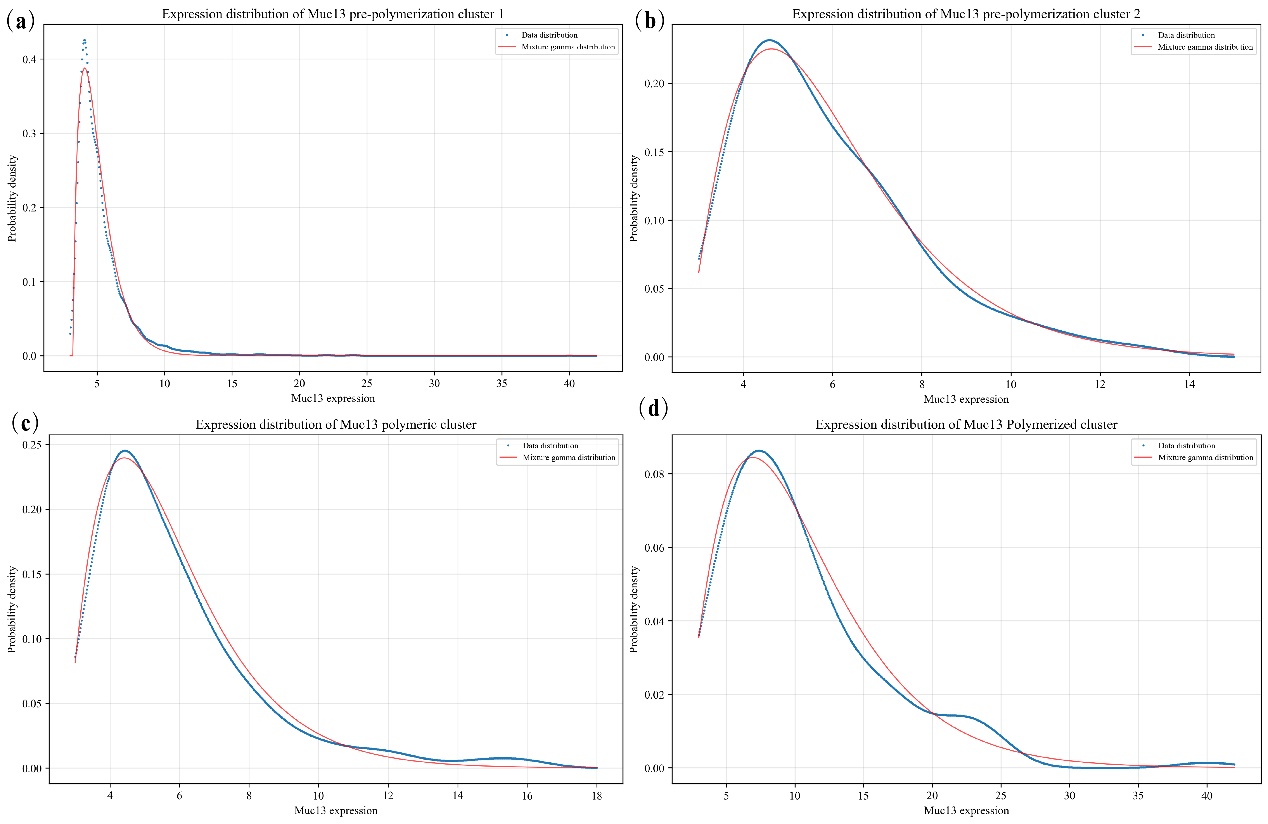


**Fig. S27** Different modes of the mRNA distributions for key gene Muc13 before and after polymerization as well as at polymerization point. The blue dashed curves represent the distributions of data points while the red curves represent the fitting results obtained using mechanic models of gene expression.

**Table S21. Statistical quantities for the Ndrg1 gene before polymerization 1 and polymerization 2, at polymerization point, and after polymerization.**

|  | Peak position | kurtosis | skewness |
| --- | --- | --- | --- |
| Pre-polymerization cluster 1 | 0.003 | 88.0 | 8.477 |
| Pre-polymerization cluster 2 | 0.003 |  |  |
| Polymeric cluster | 0.127 | 117.848 | 9.952 |
| Polymerized cluster | 2.315 | 1.008 | 1.259 |

**Table S22. The inferred parameter values for the Ndrg1 gene before polymerization 1 and polymerization 2, at polymerization point, and after polymerization.**

|  | Distribution type | parameter | | |
| --- | --- | --- | --- | --- |
|  |  |  |
| Pre-polymerization cluster 1 | Unimodal mRNA distribution | 31.5704 | 15.8968 | 2.0584 |
| Pre-polymerization cluster 2 | Unimodal mRNA distribution | 2.1508 | 1.7797 | 3.286 |
| Polymeric cluster | Unimodal mRNA distribution | 54.1244 | 6.1601 | -4.5141 |
| Polymerized cluster | Unimodal mRNA distribution | 2.0912 | 0.2423 | 1.5628 |


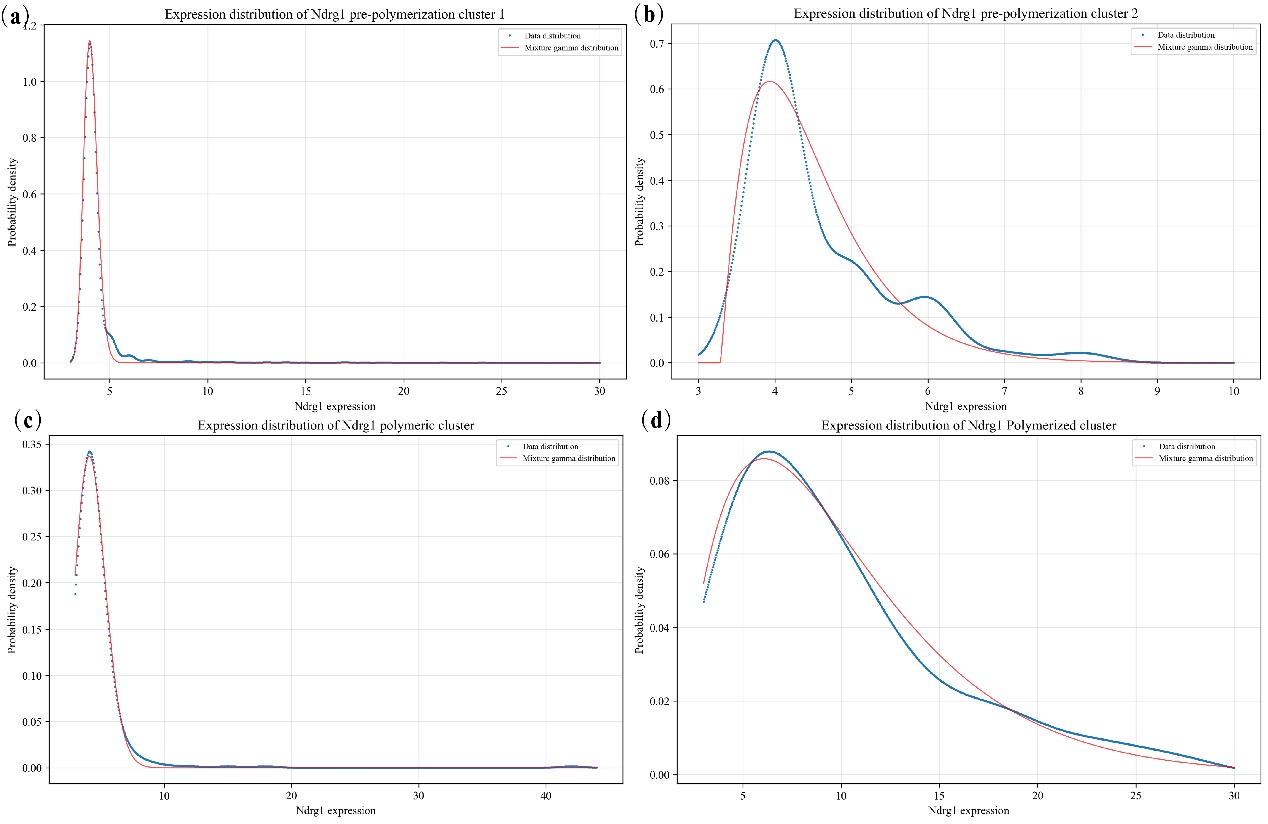


**Fig. S28** Different modes of the mRNA distributions for key gene Ndrg1 before and after polymerization as well as at polymerization point. The blue dashed curves represent the distributions of data points while the red curves represent the fitting results obtained using mechanic models of gene expression.

**
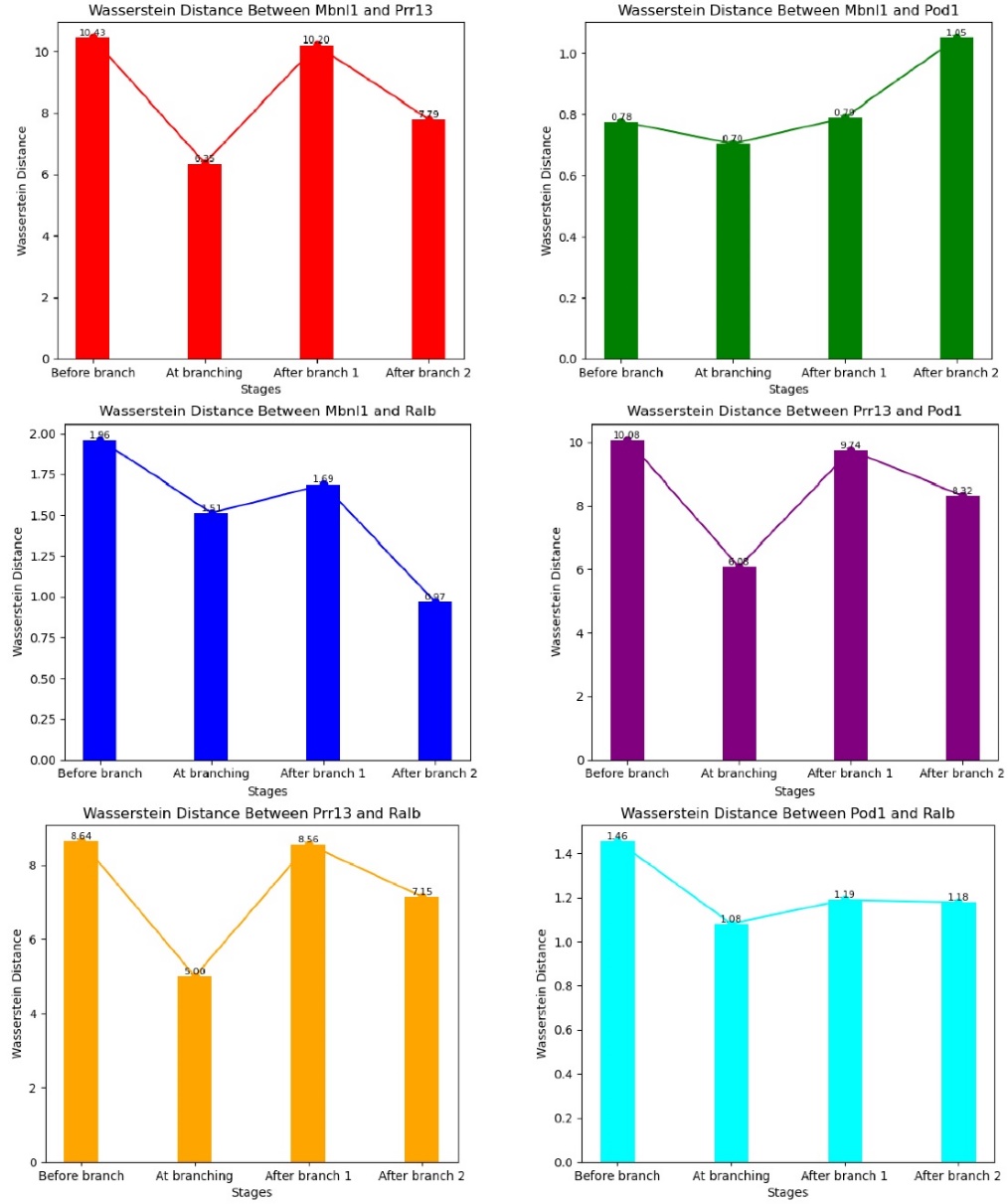
**

**Fig. S29** Wasserstein distances between Mbnl1, Pod1, Ralb and Prr13 in different stages in MEF dataset.

D. Supplemental tables

**Table S23. Wasserstein Distance Between Two Genes in Different Stages in human bone marrow dataset.**

| Stages  Wasserstein Distance | Pre-polymerization cluster 1 | pre-polymerization cluster 2 | Polymeric cluster | Polymerized cluster |
| --- | --- | --- | --- | --- |
| Gsta4 and Muc13 | 0.14 | 0.32 | 0.24 | 0.60 |
| Gsta4 and Ndrg1 | 0.27 | 0.24 | 0.31 | 0.52 |
| Gsta4 and Slc7a9 | 0.28 | 0.11 | 0.12 | 0.30 |
| Gsta4 and Clu | 0.67 | 0.15 | 0.80 | 0.15 |
| Gsta4 and Edn1 | 0.15 | 0.08 | 0.25 | 0.48 |
| Gsta4 and Atp2a3 | 0.14 | 0.07 | 0.07 | 0.38 |
| Gsta4 and H1f0 | 0.17 | 0.21 | 0.26 | 0.63 |
| Muc13 and Ndrg1 | 0.13 | 0.09 | 0.07 | 0.08 |
| Muc13 and Slc7a9 | 0.43 | 0.43 | 0.36 | 0.30 |
| Muc13 and Clu | 0.81 | 0.47 | 1.04 | 1.66 |
| Muc13 and Edn1 | 0.01 | 0.34 | 0.01 | 0.13 |
| Muc13 and Atp2a3 | 0.28 | 0.33 | 0.29 | 0.23 |
| Muc13 and H1f0 | 0.31 | 0.12 | 0.04 | 0.03 |
| Ndrg1 and Slc7a9 | 0.55 | 0.34 | 0.43 | 0.22 |
| Ndrg1 and Clu | 0.94 | 0.38 | 1.11 | 1.58 |
| Ndrg1 and Edn1 | 0.12 | 0.25 | 0.07 | 0.05 |
| Ndrg1 and Atp2a3 | 0.41 | 0.25 | 0.37 | 0.15 |
| Ndrg1 and H1f0 | 0.44 | 0.03 | 0.05 | 0.11 |
| Slc7a9 and Clu | 0.39 | 0.06 | 0.68 | 1.36 |
| Slc7a9 and Edn1 | 0.44 | 0.15 | 0.36 | 0.17 |
| Slc7a9 and Atp2a3 | 0.15 | 0.10 | 0.09 | 0.07 |
| Slc7a9 and H1f0 | 0.11 | 0.31 | 0.38 | 0.33 |
| Clu and Edn1 | 0.82 | 0.15 | 1.04 | 1.53 |
| Clu and Atp2a3 | 0.53 | 0.14 | 0.74 | 1.43 |
| Clu and H1f0 | 0.50 | 0.35 | 1.06 | 1.69 |
| Edn1 and Atp2a3 | 0.29 | 0.07 | 0.30 | 0.10 |
| Edn1 and H1f0 | 0.32 | 0.23 | 0.04 | 0.16 |
| Atp2a3 and H1f0 | 0.03 | 0.22 | 0.32 | 0.26 |

**Table S24. Wasserstein Distance Between Two Genes in Different Stages in intestine organoid dataset.**

| Stages  Wasserstein  Distance | Before branch 1 | At branching 1 | After branch 1 |
| --- | --- | --- | --- |
| ATP2A3 and H1F0 | 1.27 | 1.35 | 1.29 |
| ATP2A3 and CD44 | 1.27 | 1.35 | 1.28 |
| ATP2A3 and CELF2 | 1.24 | 1.32 | 1.25 |
| ATP2A3 and TAOK3 | 0.75 | 0.70 | 0.53 |
| ATP2A3 and CNN3 | 0.97 | 1.07 | 1.01 |
| ATP2A3 and SCUBE2 | 1.18 | 1.18 | 1.18 |
| ATP2A3 and SMIM1 | 0.29 | 0.45 | 0.32 |
| H1F0 and CD44 | 0.01 | 0.004 | 0.01 |
| H1F0 and CELF2 | 0.05 | 0.05 | 0.06 |
| H1F0 and TAOK3 | 0.63 | 0.77 | 0.81 |
| H1F0 and CNN3 | 0.35 | 0.36 | 0.34 |
| H1F0 and SCUBE2 | 0.11 | 0.20 | 0.13 |
| H1F0 and SMIM1 | 1.03 | 1.01 | 1.00 |
| CD44 and CELF2 | 0.05 | 0.05 | 0.05 |
| CD44 and TAOK3 | 0.62 | 0.76 | 0.80 |
| CD44 and CNN3 | 0.34 | 0.35 | 0.33 |
| CD44 and SCUBE2 | 0.11 | 0.19 | 0.12 |
| CD44 and SMIM1 | 1.02 | 1.00 | 0.97 |
| CELF2 and TAOK3 | 0.59 | 0.73 | 0.77 |
| CELF2 and CNN3 | 0.31 | 0.31 | 0.28 |
| CELF2 and SCUBE2 | 0.06 | 0.15 | 0.07 |
| CELF2 and SMIM1 | 0.99 | 0.97 | 0.96 |
| TAOK3 and CNN3 | 0.30 | 0.44 | 0.51 |
| TAOK3 and SCUBE2 | 0.52 | 0.58 | 0.70 |
| TAOK3 and SMIM1 | 0.48 | 0.28 | 0.23 |
| CNN3 and SCUBE2 | 0.24 | 0.16 | 0.21 |
| CNN3 and SMIM1 | 0.72 | 0.70 | 0.71 |
| SCUBE2 and SMIM1 | 0.93 | 0.83 | 0.89 |

References

Daalhuis ABO. Confluent hypergeometric functions. *NIST handbook of mathematical functions.* 2010; **321**:349.

Gillespie DT. A general method for numerically simulating the stochastic time evolution of coupled chemical reactions. *J Comput Phys.* 1976; **22**:403-434.
