# Supplementary material for: Marker genes reveal dynamic features of cell evolving processes
Source: Bioinform Adv. 2025 Aug 5;5(1):vbaf185. doi: 10.1093/bioadv/vbaf185 (PMC12396811; doi:10.1093/bioadv/vbaf185)
Supplement: vbaf185_Supplementary_Data [file vbaf185_supplementary_data.zip › Supplemental Material.pdf]

Supplementary Material

Marker genes reveal dynamic features of cell evolving processes

Wenjie Cao, Bengong Zhang, Tianshou Zhou*

Corresponding authors: Tianshou Zhou, School of Mathematics, Sun Yat-sen University, Guangzhou 510275, China. Tel.:/Fax: +86-13710636599; E-mail: mcszhtsh@mail.sysu.edu.cn

Contents

A. Derivation of mRNA distributions 1
B. Methods........ 3
B1. Model selection 3
B2. Selection criteria for marker genes 3
C. Supplemental texts and figures........ 4
C1. Figures and Tables related to the Method and Material in the main text 4
C2. Mouse embryo cells........ 5
C3. Mouse embryonic fibroblasts........ 6
C4. Human bone marrow 12
C5. Intestine organoid........ 18
D. Supplemental tables 26
References 28

A. Derivation of mRNA distributions

First, we consider the common two-state model of gene expression at the transcription level:

$$\begin{aligned} &\text{OFF} \xrightarrow{\alpha} \text{ON}, \quad \text{ON} \xrightarrow{\beta} \text{OFF} \\ &\text{ON} \xrightarrow{\mu} \text{ON} + \text{mRNA}, \quad \text{mRNA} \xrightarrow{\delta} \varnothing, \end{aligned} \tag{A1}$$

where α and β are switching rates between two states of the promoter, μ and δ are the transcription and degradation rates of the mRNA. Without loss of generality, we assume $\delta = 1$. If the mRNA distribution at steady state is denoted by $P(m)$, then it can be analytically expressed as

$$P(m) = \frac{\Gamma(\alpha+m)\Gamma(\alpha+\beta)}{\Gamma(\alpha+\beta+m)\Gamma(\alpha)} \frac{1}{m!} \mu^m \, {}_1F_1(\alpha+m, \alpha+\beta+m; -\mu), \tag{A2}$$

where ${}_1F_1(a,b;z) = \sum_{n=0}^{\infty} \frac{(a)_n}{(b)_n} \frac{z^n}{n!}$ is a confluent hypergeometric function (Daalhuis,2010), and we

have defined $(c)_n = c(c+1)\cdots(c+n-1)$. Note that the expression of $P(m)$ is very complex in form since it involves a confluent hypergeometric function of the form ${}_1F_1(a,b;z)$. This brings difficulties for the statistical inference of model parameters based on scRNA-seq data.

In the following, we simply derive an approximate distribution of mRNA. If the production rate μ is sufficiently large compared with the other reaction rates, then discrete variable m can be taken as a continuous variable (re-denoted by x). Under this assumption, we can use a continuous equation to approximate x:

$$\frac{\mathrm{d}x}{\mathrm{d}t} = -x + \mu f(t), \tag{A3}$$

where $f(t) \in \{0,1\}$ represents the random telegraph signal of gene activation (corresponding $f(t)=1$) and inactivation (corresponding $f(t)=0$). Note that the switching rates from inactivation and activation and vice versa are α and β respectively. For convenience, we introduce two functions, which are defined below

$$\begin{aligned} P_A(x,t)\mathrm{d}x &= \mathrm{Pr\,ob}\left\{ y \in (x, x+\mathrm{d}x) \middle| \ f(t)=1 \text{ at time } t \right\}, \\ P_I(x,t)\mathrm{d}x &= \mathrm{Pr\,ob}\left\{ y \in (x, x+\mathrm{d}x) \middle| \ f(t)=0 \text{ at time } t \right\}, \end{aligned} \tag{A4}$$

where $\mathrm{d}x$ is an infinitesimal increment, $P_A(x,t)$ and $P_I(x,t)$ represent the probabilities that the value of mRNA is x in active and inactive states respectively. Set $P = P_A + P_I$, which is the total probability. Note that the probability flux can be expressed by synthesis and degradation rates combined with two factorial probabilities $P_A(x,t)$ and $P_I(x,t)$, that is,

$$\begin{aligned} J_A(x,t) &= (\mu - x)P_A(x,t), \\ J_I(x,t) &= -xP_I(x,t). \end{aligned} \tag{A5}$$

Then, according to the conservative condition for probability flux, we have

$$\frac{\partial P_A}{\partial t} + \frac{\partial J_A}{\partial x} = \alpha P_I - \beta P_A, \qquad \frac{\partial P_I}{\partial t} + \frac{\partial J_I}{\partial x} = -\alpha P_I + \beta P_A. \tag{A6}$$

This is a mathematical equation for a gene that has one active and one inactive states (this model can be easily extended to the case that a gene has multiple inactive and active states).

Next, we find the equilibrium solution to Eq. (A6). At steady state, the sum of two equations yields

$$\frac{\partial}{\partial x}(\mu P_A - xP) = 0, \tag{A7}$$

from which we know that $\mu P_A - xP$ must be a constant independent of x. Since both $P_A(x)$ and $P(x)$ tend to zero as x goes to infinity, we have $\mu P_A - xP \equiv 0$ for any $x > 0$. Thus, we obtain the relationship between $P_A(x)$ and $P(x)$: $P_A = \frac{x}{\mu}P$ or $P_I = \left(1 - \frac{x}{\mu}\right)P$. Making use of this relationship, it follows from Eq. (A6) that

$$\frac{\partial}{\partial x}\left(-x\left(1-\frac{x}{\mu}\right)P\right)=\left(-\alpha\left(1-\frac{x}{\mu}\right)+\beta\frac{x}{\mu}\right)P. \tag{A8}$$

By solving this equation, we arrive at

$$P(x)=\mu^{1-\alpha-\beta}\frac{\Gamma(\alpha+\beta)}{\Gamma(\alpha)\Gamma(\beta)}x^{\alpha-1}(\mu-x)^{\beta-1}, \tag{A9}$$

indicating that mRNA follows a Beta distribution. Apparently, $P(x)$ may be unimodal or bimodal, depending on the choice of α and β values.

If the deactivation rate β is much larger than the activation rate α and is larger than the degradation rate $\delta=1$, then distribution $P(x)$ can be further approximated as the following Gamma distribution

$$P(x)=\frac{\tilde{\beta}^{\alpha}}{\Gamma(\alpha)}x^{\alpha-1}e^{-\tilde{\beta}x}, \tag{A10}$$

which is determined by two parameters α and $\tilde{\beta}$ $(=\beta/\mu)$. Moreover, $\tilde{\beta}$ represents the average size of the transcription factor production bursts (i.e., the mean burst size) and α represents the mean number of transcription factor (TF) production bursts per cell (i.e., the inverse of the mean burst frequency).

B. Method

B1. Model selection

We use the Kernel Density Estimation (KDE) method to perform estimations for each of the four datasets in the main text. As is well known, the KDE is a non-parametric statistical method and can be used to estimate an unknown probability density function. This method does not rely on any specific assumptions on probability distribution, so it can be applied to any type of dataset.

Given $x_1,x_2,\cdots,x_N$ as n independent and identically distributed samples, if we let their probability density function be f, then the KDE takes the form

$$\hat{f}_h(x)=\frac{1}{n}\sum_{i=1}^{n}K_h(x-x_i)=\frac{1}{nh}\sum_{i=1}^{n}K\left(\frac{x-x_i}{h}\right), \tag{A11}$$

where $K(\cdot)$ is the kernel function (non-negative, with the integral 1, satisfying the properties of a probability density and having the zero mean); h is referred to as the bandwidth and is sometimes called a window in calculation.

B2. Selection criteria for marker genes

1. The gene should rank high in differential expression within the corresponding cell cluster.

2. The gene should exhibit strong expression specificity, meaning that it has a high expression rate in the corresponding cell cluster and a low expression rate in other clusters.

C. Supplemental texts and figures

We assume that for each dataset, the information on the entire development process is adequate, or a snapshot of primary tissue represents a complete developmental process.

C1. Figures and Tables related to the Method and Material in the main text

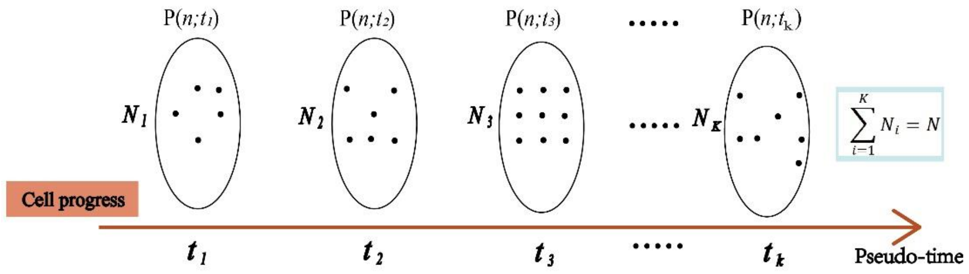

Fig. S1 Calculation of the mRNA (the product of some key gene) distribution $P_i(m;t_k)$ at time point t_k (practically a time window) along the ith single pseudo-trajectory. Similarly, we can calculate distribution $P_i(m)$ for the entire single pseudo-trajectory, which is actually a weighted mixture of the distributions at all pseudo-time points of this single pseudo-trajectory.

The linear combination of two Gamma distributions can be used to well fit a bimodal distribution (Fig. S2).

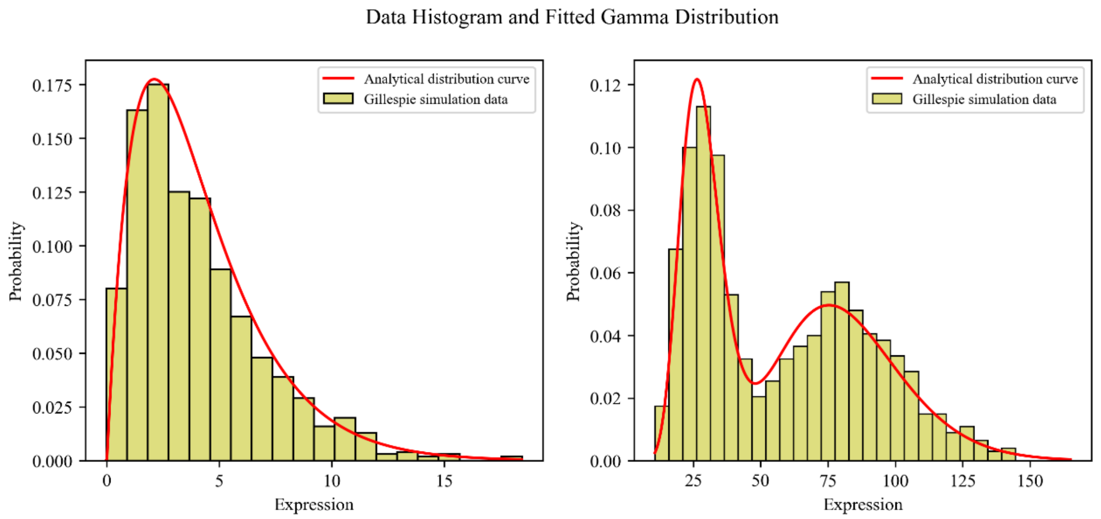

Fig. S2 Unimodal and bimodal mRNA distributions, where histograms represent the numerical results obtained by the famous Gillespie stochastic algorithm whereas the red curves represent the results predicted by analytical distribution (Gillespie,1976). Parameter values are set as $\alpha = 2.2373, \beta = 0.5426$ in the case of unimodal mode, and $\omega_1 = 0.4520$, α_1=13.74, β_1=0.4835, $\omega_2 = 0.548$, α_2=12.87996775, β_2=0.1575 in the case of bimodal mode.

Table S1. Marker genes in mouse embryonic cells

	Genes with different distribution characteristics before and after branching	Highly variable gene	Marker gene
Pdgfa	Y	—	—
Cdx2	Y	—	—
Eomes	Y	—	—
Ahcy	Y	—	—
Lcp1	Y	—	—

Fgfr2	Y	—	—
Klf5	Y	—	—
Actb	Y	Y	Y
Dab2	Y	Y	Y
Dppa1	Y	Y	Y
Nanog	Y	Y	Y
Gapdh	Y	Y	Y
Id2	Y	Y	Y
Pou5f1	Y	Y	Y
Tspan8	Y	Y	Y
Sox2	Y	Y	Y
Gata3	Y	Y	Y
Sall4	Y	Y	Y

C2. Mouse embryo cells

The Gata3 plays a key role in the formation and differentiation of the endoderm and ectoderm, guiding cells towards specific fates by regulating the expression of target genes. Usually, the Gate3 expression level is relatively high. The Sox2 protein is especially important for the development of the eyes. This protein regulates the activity of other genes by attaching (binding) to specific regions of DNA. In addition, Cyclin-dependent kinase inhibitor p21 controls adult neural stem cell expansion by regulating Sox2 gene expression.

As can be seen from Fig. S3(a-c) in ME dataset and MEF (see Fig. S4(a-c)) dataset, the branching pattern is repeatable in multiple embedding technologies (pca, umap, t-sne) and the trace branching phenomenon can be obviously observed in the cluster graph. We took 5 different sets of variable gene subsets (80%) to define the trajectories(referring to Fig. S3(d)). When observing the visual locus after dimensionality reduction, it was found that the branch points still existed stably, ensuring the stability and elasticity of the fulcrum when information was lost. Moreover, the 5 randomly selected gene subsets ensured the statistical robustness of the branch points. For ME dataset, the cluster label is used as given in the reference (Wei et al.,2021). For the clustering and pseudo-time graph of this dataset, our result graph is similar to the result of this literature (see Fig. 3 in reference(Wei *et al*.,2021)). For MEF dataset, we find that the pseudo-time calculated by scVelo algorithm is coarse-granulated. By clustering the dataset in three different ways and coloring the labels coarse-granulated according to pseudo-time, obvious branching phenomena can be found to ensure that the identified branches are real in the data and not the product of dimensionality reduction process, and the MEF data itself has differentiation phenomenon, see reference (Luo et al.,2023).

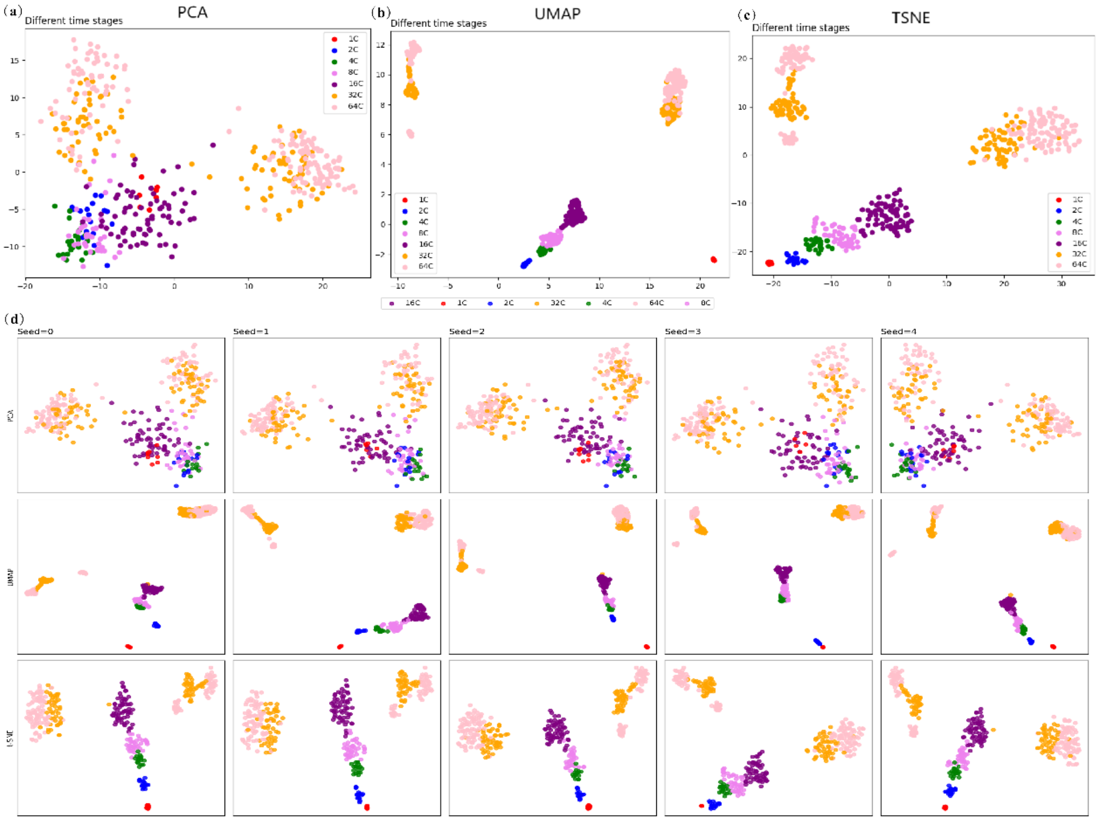

Fig. S3 Visualization of clustering with full genes and clustering results of 5 random gene sets(80%) in mouse embryonic cells. (a) PCA clustering of mouse embryonic cells. (b) TSNE clustering of mouse embryonic cells. (c) UMAP of mouse embryonic cells. (d) Clustering results of 5 random gene sets(80%).

We observe from Fig. 5 that before the branch, there is a distinct peak (referring to Fig. 5(a)); at the branching point, there are one moderately high peak and one higher peak (Fig. 5(b)); after the branch, there are three peaks for two different branches (Fig. 5(c) and Fig. 5(d)). The numbers of peaks in branch 1 and branch 2 are relatively similar, but there is a significant difference between the peak heights, indicating that these two clusters after differentiation would have considerable differences in functions.

Although we merely analyzed the joint probability distributions of key genes Gata3 and Sox2 before and after bifurcations as well as branching points, the differences between these distributions would imply that different molecular mechanisms govern the evolutionary processes of mouse embryonic cells before and after bifurcations.

The GENIE3 is a tree-based model that yields a separate ranking of the genes as potential regulators of a target gene in the form of weights ω_{ij} computed as sums of total variance reductions. From Fig. 6(a) we observe that with cell progression, the regulatory strength of Gata3 over Sox2 first increases slowly (i.e., with a smaller slope) before branch and then increases sharply (i.e., with a larger slope) after branch. Different from the case of Gata3 over Sox2, the regulatory strength of Sox2 over Gata3 is almost a constant before branch but increases significantly after branch, seeing to Fig. 6(b). The results shown in Fig. 6(a,b) indicate that the interaction between marker genes

Gata3 and Sox2 before bifurcation is in general weak and the interaction strength is in general small. By contrast, the interaction between Gata3 and Sox2 after bifurcation becomes stronger and the interaction strength becomes more and more with the further evolution of cells. That is, the interaction strength between these two key genes significantly increases in the later stages of differentiation, and their mutual regulation shows a trend of synchronous change. In addition, the interaction strength is stronger in one branch than in the other branch.

Fig. 7(a,b) indicates that gene Gata3 promotes the expression of gene Sox2 but the latter seems not to affect the expression of the former before bifurcation, i.e., the interaction between these genes is unidirectional before bifurcation. After bifurcation, however, the interaction between gene Gata3 and gene Sox2 are bidirectional. Moreover, the former enhances the expression of the latter that also enhances the expression of the former. Thus, according to this kind of regulation relationship, we can analyze the time-evolutional dynamics of the module of these two genes after branch by establishing a chemical master equation for this module. The details are omitted here.

From Fig. 7(c,d), we observe that the Wasserstein distance between Gata3 and Sox2 is smaller before branch, indicating that the activation interval of these two genes during the cell evolving process is relatively close before branch. With cell progression, the Wasserstein distance reaches the minimum during a branching stage, suggesting that the activation interval between these two genes becomes more distant during this process. After bifurcation, however, the Wasserstein distance between the two branches becomes closer again, indicating that Gata3 and Sox2 still exhibit a relatively similar gene-activated process during different cell stags.

In eukaryotic cells, most genes are expressed in a bursty fashion, i.e., gene products (mRNAs or proteins) are produced in episodes of high transcriptional activity followed by long periods of inactivity. This bursting kinetics is usually quantified by burst size and burst frequency. Therefore, we focus on the calculation of mean burst sizes and frequencies of key genes along the reconstructed pseudo-time trajectories. By this calculation, we try to see whether there are any changes before and after branches.

Regarding bursting kinetics, we supplement the following contents for better understanding. When a gene is in an active promoter state, it is assumed to transcribe at a rate, s (per unit time), and the number of mRNA molecules of the gene is assumed to decay at a rate, d (per unit time). Subsequently, transcriptional bursting can be characterized by two parameters: the average number of synthesized mRNA molecules while the gene remains in the active state (burst size or transcriptional efficiency, $\mathrm{s/k_{off}}$) and the frequency at which bursts occur per unit time (burst frequency, $\mathrm{k_{on}}$).

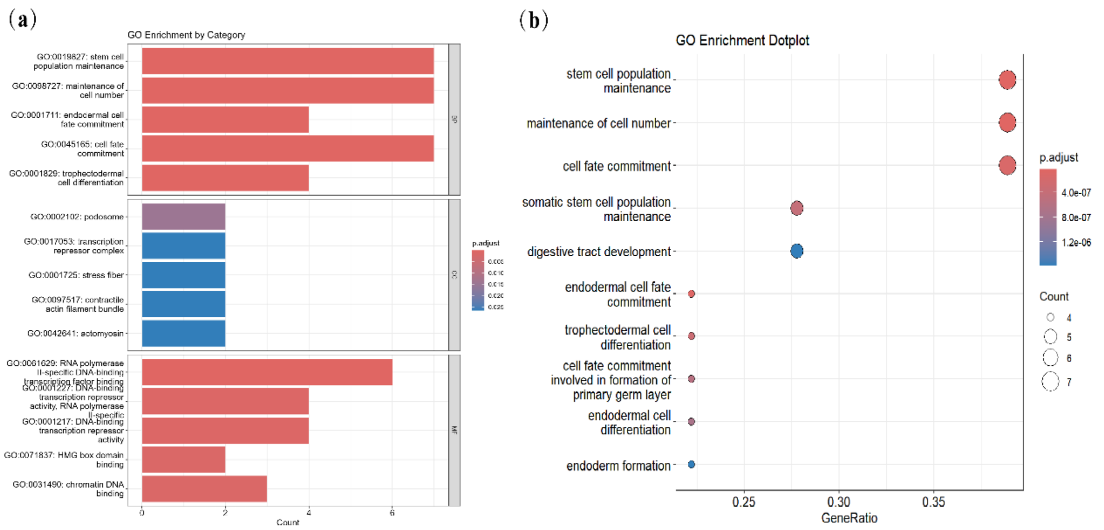

Fig. S4 GO analysis results of marker gene in mouse embryonic cells. (a) GO enrichment by category, BP represents biological process, CC means cell component, MF stands for molecular function. The value of P.adjust is the correction value of false discovery rate in gene set enrichment analysis. (b) GO enrichment dotplot.

We performed a GO Biological Process analysis on the genes with p-values less than 0.05 in this dataset. Marker genes are mainly associated with stem cell population maintenance (GO:0019827), maintenance of cell number (GO:0098727) and cell fate commitment (GO:0045165) (Fig. S4) for biological process. There is significant enrichment in podosome for cell component (GO: 0002102), it is a dynamic structure for the interaction between cells and the extracellular matrix, mainly present in migratory cells (such as immune cells, tumor cells, etc.). Composed of actin filaments and others, it plays an important role in cell adhesion, migration, and invasion. The molecular function of RNA polymerase II-specific DNA-binding transcription factor activity (GO:0061629) plays a key role in gene expression regulation.

C3. Mouse embryonic fibroblasts

Clusters one to four are pre-branch clusters, cluster five is a cluster at the branching point, and clusters six and seven, along with clusters eight and nine, respectively, represent the two parallel post-branch clusters. Under default parameters, the clustering effects of PCA, TSNE, and UMAP are not optimal, but the cells still exhibit distinct bifurcation characteristics, refer to Fig. S5 for cell pseudo-trajectories constructed using scRNA-seq data, and Fig. S6-S11 and Table. S2-S4 for the mRNA expression patterns of key genes along the pseudo-trajectories.

The mRNA distributions of gene Mbn1 for one branch and before branch are all bimodal, the mRNA distributions of gene Pod1 for two branches and before branch are all unimodal, and the mRNA distributions of genes Ralb for two branches and before branch are all unimodal. Moreover, there is indeed a branch such that the mRNA distribution of each of these key genes for this branch is the same as that before bifurcation. The same mode of the distributions before and after branch

indicates the fact that the genetic information can be still retained in the complex evolutionary process of cells. However, we point out that in spite of the same mode, the distributions are actually different since their peakness and skewness are different, referring to Fig. S6, S8 and S10.

The Wasserstein Distance between Mbnl1 and Pod1 shows no significant change before branch and at the branching point, but it increases in cluster 1 after branch, indicating that these two genes are not continuously activated in the cell evolutionary process of this branch. In contrast, Pod1 and Ralb have a large Wasserstein Distance before branch, which gradually decreases along the cell pseudo trajectories. See Fig. S29. The Wasserstein distance between key genes in the remaining two datasets is shown in Tables. S25 and S26.

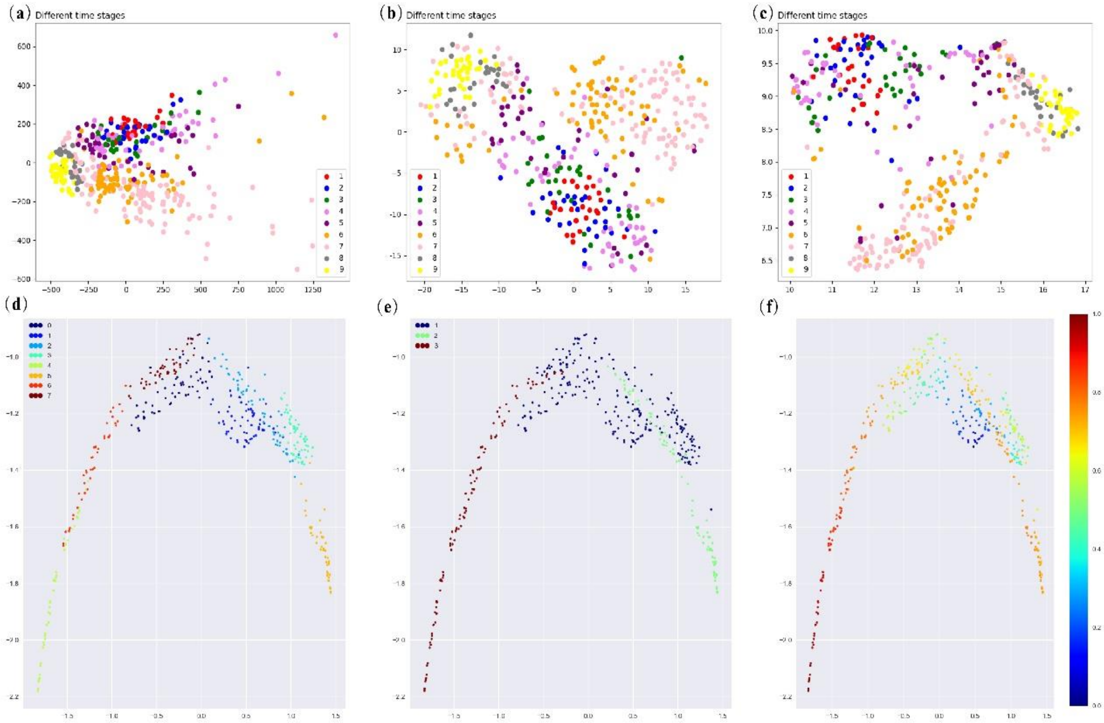

Fig. S5 Visualization of clustering and branching trajectories in mouse embryonic fibroblasts cells. (a) PCA clustering of mouse embryonic fibroblasts. (b) TSNE clustering of mouse embryonic fibroblasts. (c) UMAP of mouse embryonic fibroblasts. (d) Stage clustering. (e) Branch identification. (f) Pseudo-time inference clustering visualization.

Table S2. The inferred parameter values for the Mbnl1 gene before branch, at branching point, and after branch 1 and branch 2.

	Distribution type	parameter					
		$\omega 1$	$\alpha 1$	$\beta 1$	$\alpha 2$	$\beta 2$	loc
Before branch	Bimodal mRNA distribution	0.8124	1.2614	3.4856	24.0102	1.6297	
Branch point	Unimodal mRNA distribution		0.178	2.232			-1.0
Branch 1	Bimodal mRNA distribution	0.7552	1.316	2.446	1.0	94.4437	
Branch 2	Unimodal mRNA distribution		0.781	1.980			-1.0

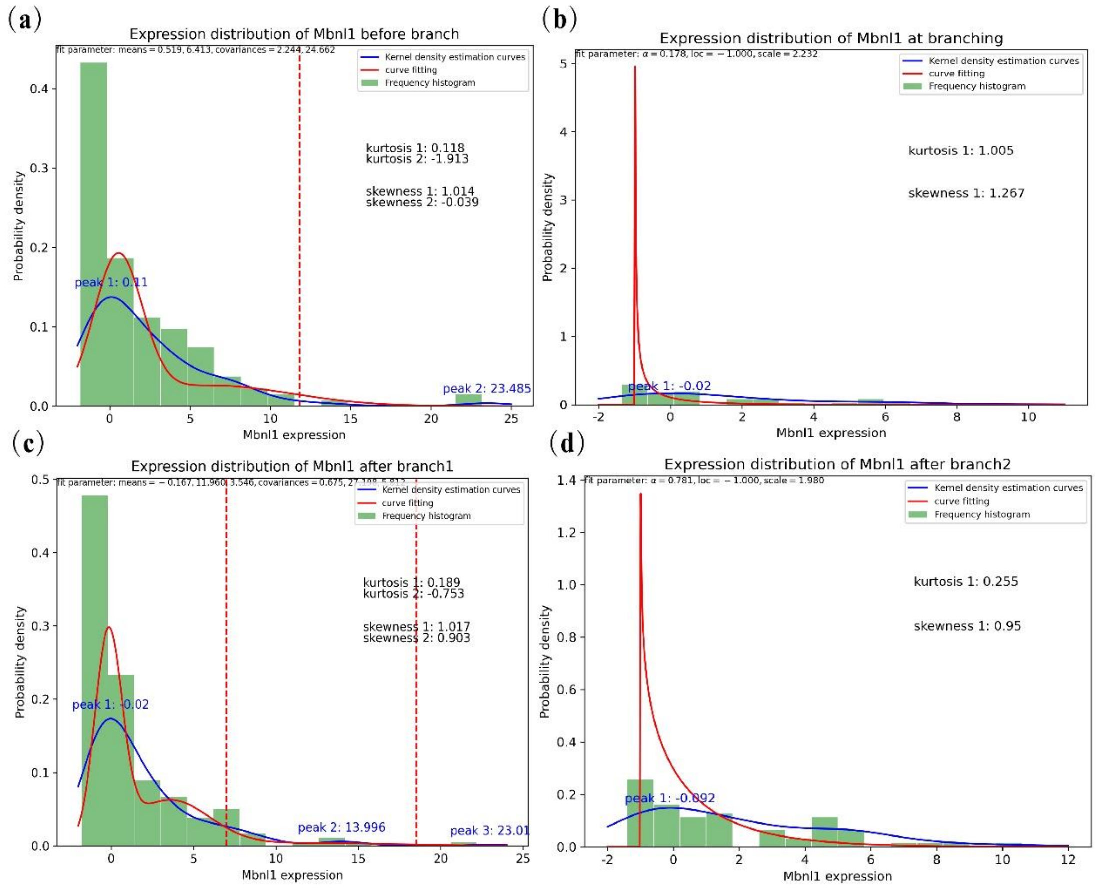

Fig. S6 Statistical quantities for the Mbnl1 gene before branch, at branching point, and after branch 1 and branch 2.

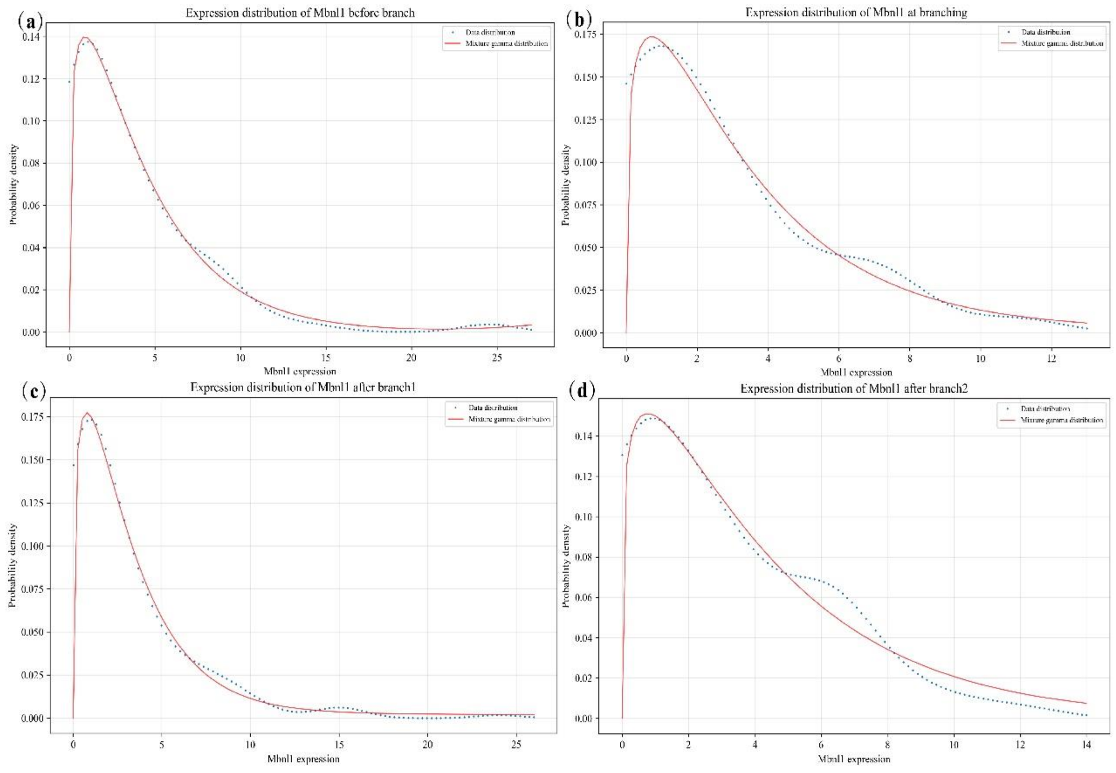

Fig. S7 Different modes of the mRNA distributions for key gene Mbnl1 before and after branch as well as at branching point. The blue dashed curves represent the distributions of data points while

the red curves represent the fitting results obtained using mechanic models of gene expression.

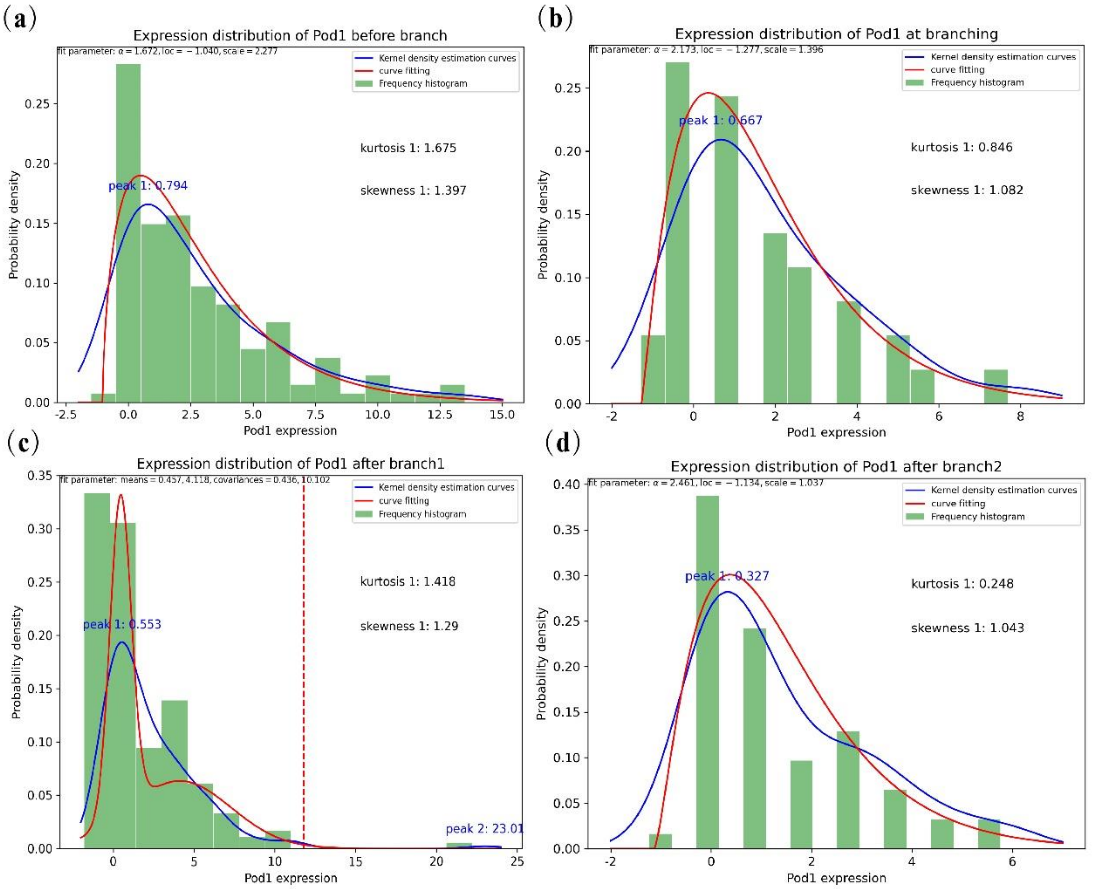

Fig. S8 Statistical quantities for the Pod1 gene before branch, at branching point, and after branch 1 and branch 2.

Table S3. The inferred parameter values for the Pod1 gene before branch, at branching point, and after branch 1 and branch 2.

	Distribution type	parameter					
		$\omega 1$	$\alpha 1$	$\beta 1$	$\alpha 2$	$\beta 2$	*loc*
Before branch	Unimodal mRNA distribution		1.672	2.277			-1.04
Branch point	Unimodal mRNA distribution		2.173	1.396			-1.277
Branch 1	Bimodal mRNA distribution	0.9264	1.5926	2.2572	26.8076	1.339	
Branch 2	Unimodal mRNA distribution		2.461	1.037			-1.134

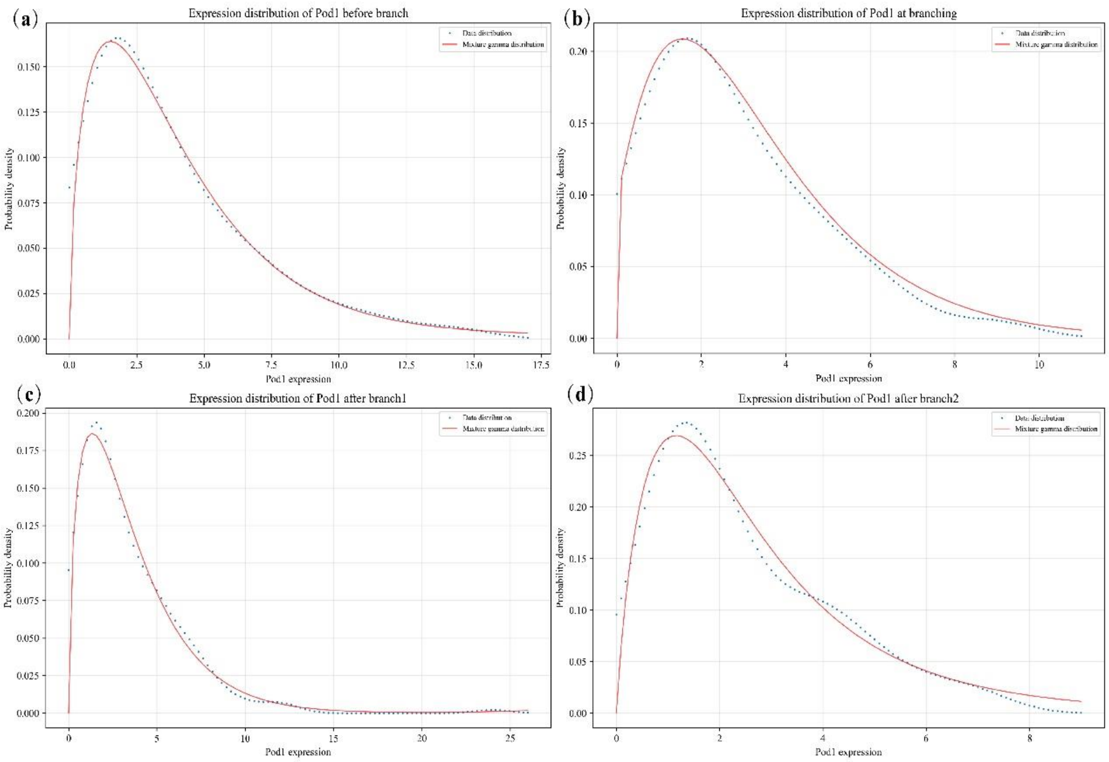

Fig. S9 Different modes of the mRNA distributions for key gene Pod1 before and after branch as well as at branching point. The blue dashed curves represent the distributions of data points while the red curves represent the fitting results obtained using mechanic models of gene expression.

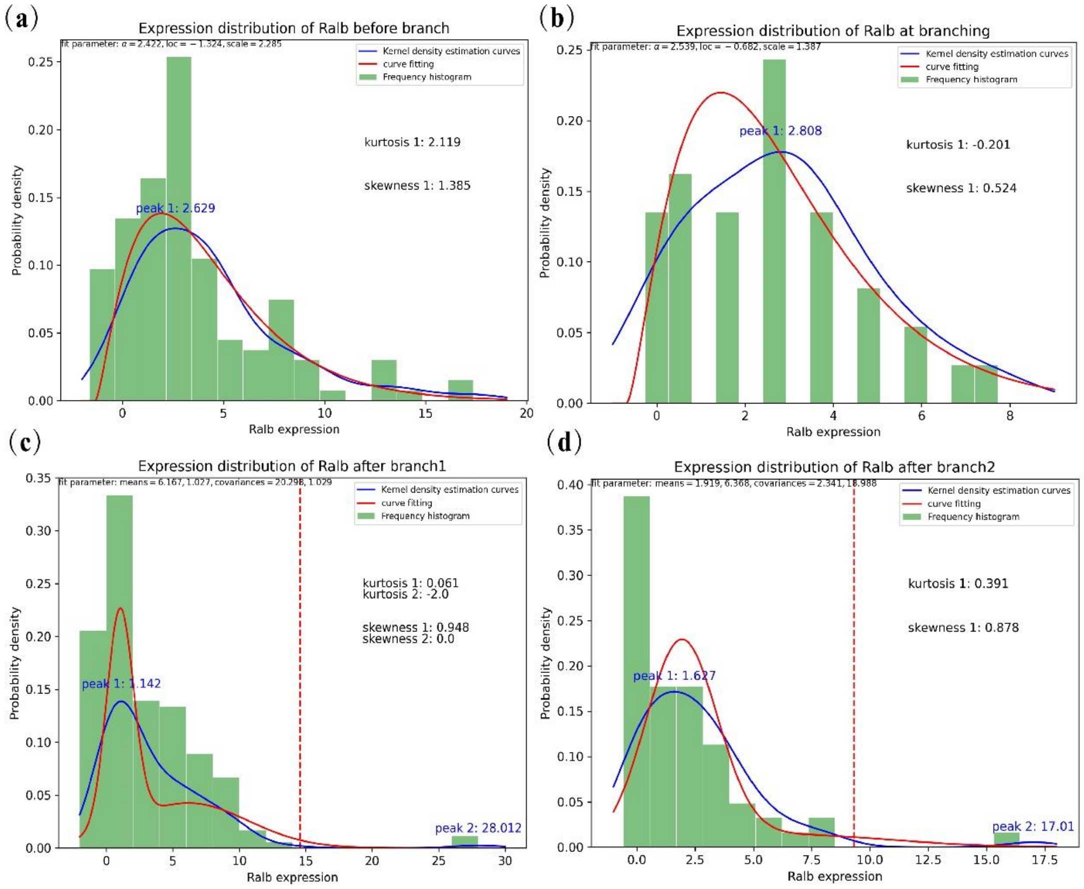

Fig. S10 Statistical quantities for the Ralb gene before branch, at branching point, and after branch 1 and branch 2.

Table S4. The inferred parameter values for the Ralb gene before branch, at branching point, and after branch 1 and branch 2.

	Distribution type	parameter					
		$\omega1$	$\alpha1$	$\beta1$	$\alpha2$	$\beta2$	loc
Before branch	Unimodal mRNA distribution		2.422	2.285			-1.324
Branch point	Unimodal mRNA distribution		2.539	1.387			-0.682
Branch 1	Bimodal mRNA distribution	0.9259	1.548	3.2449	28.6523	1.4348	
Branch 2	Bimodal mRNA distribution	0.3768	7.5322	0.6736	2.1784	1.8504	

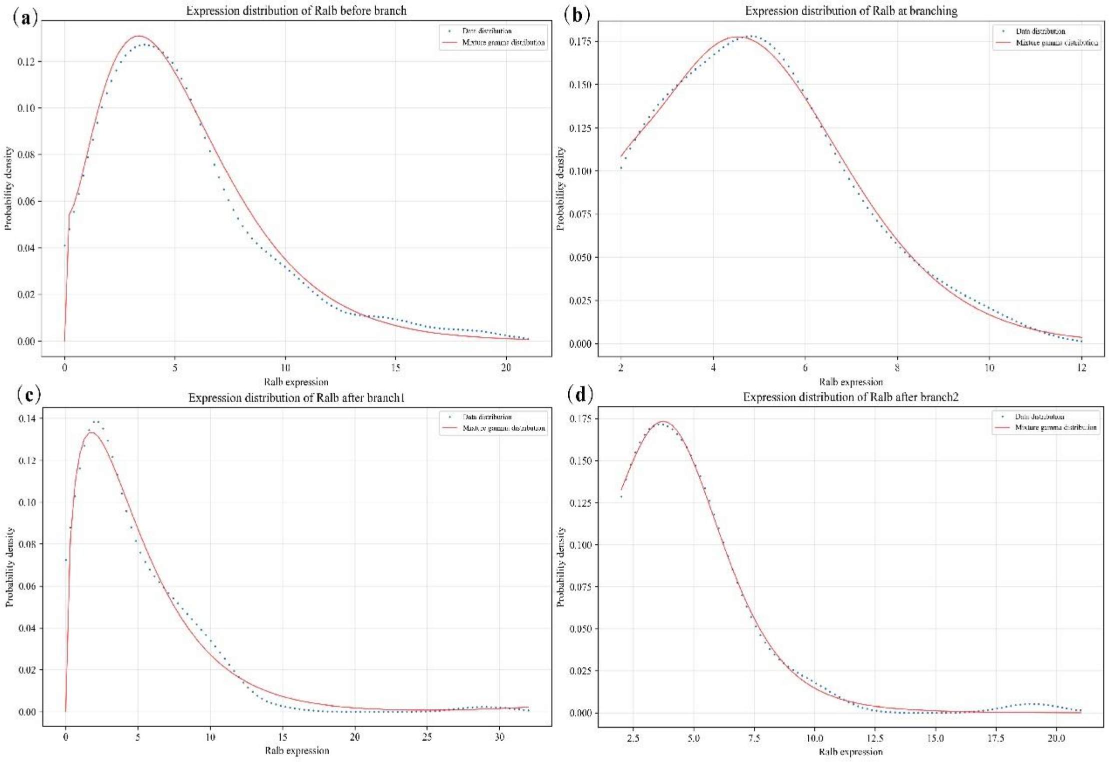

Fig. S11 Different modes of the mRNA distributions for key gene Ralb before and after branch as well as at branching point. The blue dashed curves represent the distributions of data points while the red curves represent the fitting results obtained using mechanic models of gene expression.

C4. Human bone marrow

Based on pseudo-time values and prior knowledge of the data type, it is known that cells differentiate from cluster zero to cluster one, which further differentiates into clusters two, three, four, and five. The second bifurcation occurs from cluster one to cluster four, which then differentiates into clusters six and seven. Clusters five, six, seven, eight, and nine are the terminal clusters, which correspond to large pseudo-time values. Refer to Fig. S12 for cell pseudo-trajectories constructed using scRNA-seq data, and Fig. S13-S20 and Table. S5-S8 for the mRNA expression patterns of key genes along the pseudo-trajectories.

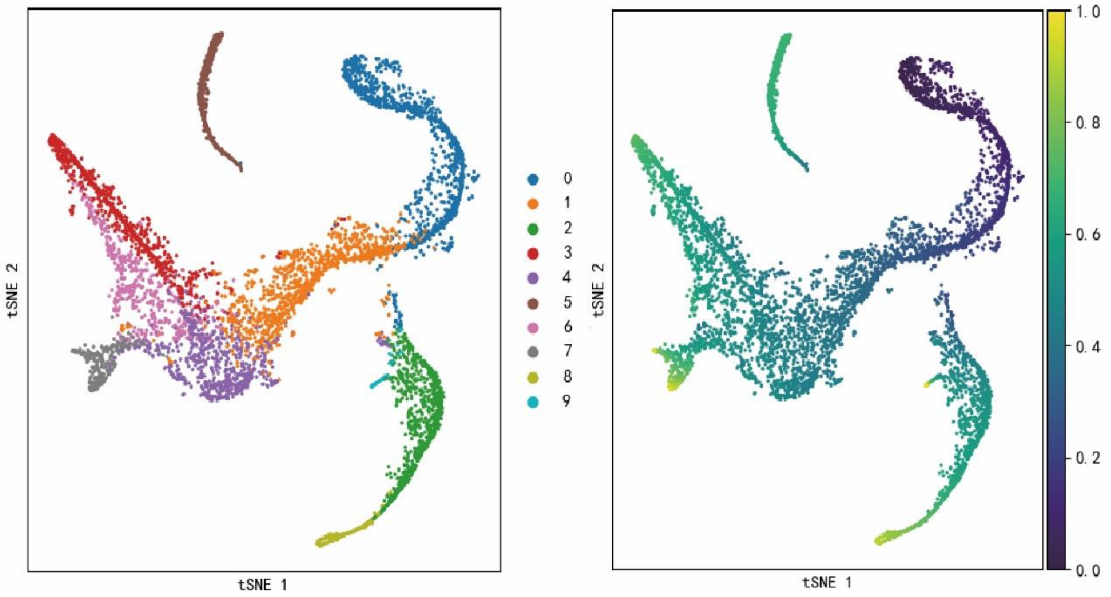

Fig. S12 Visualization of clustering and Pseudo-time inference clustering visualization in human bone marrow dataset.

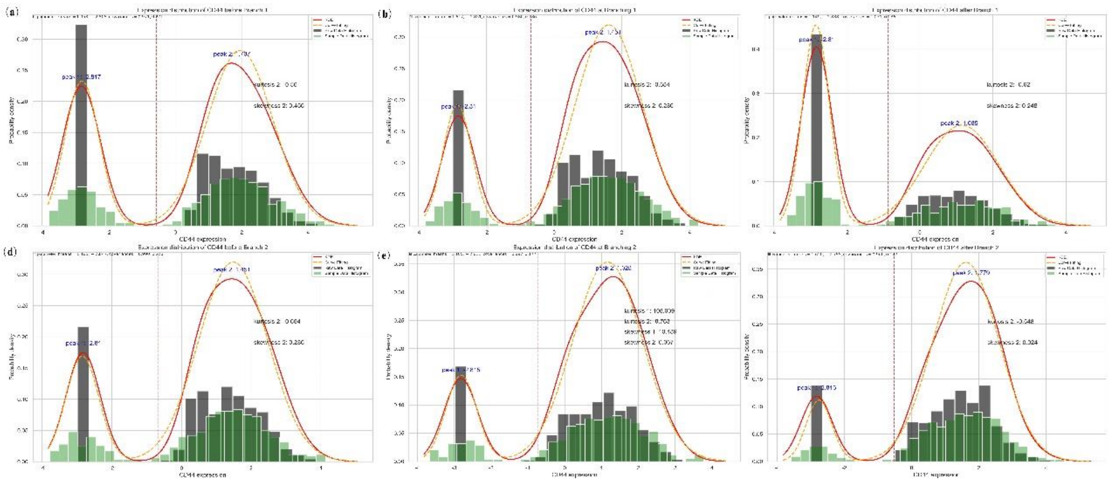

Fig. S13 Statistical quantities for the CD44 gene before branch 1, at branching point 1, and after branch 1 and branch before branch 2, at branching point 2, after branch 2.

Table S5. The inferred parameter values for the CD44 gene before branch 1, at branching point 1, and after branch 1 and before branch 2, at branching point 1, and after branch 2.

	Distribution type	parameter				
		$\omega 1$	$\alpha 1$	$\beta 1$	$\alpha 2$	$\beta 2$
Before branch 1	Bimodal mRNA distribution	0.3097	16.2111	7.7760	42.7084	6.2893
Branch point 1	Bimodal mRNA distribution	0.2121	18.0700	9.0559	34.1021	5.3060
After branch 1	Bimodal mRNA distribution	0.4307	22.6373	11.4480	33.9341	5.6642
Before branch 2	Bimodal mRNA distribution	0.2235	18.5079	9.2015	26.4583	4.1372
Branch	Bimodal mRNA	0.4307	22.6373	11.4480	33.9341	5.6642

point 2	distribution					
After branch 2	Bimodal mRNA distribution	0.1368	19.7204	9.8576	34.1461	5.2899

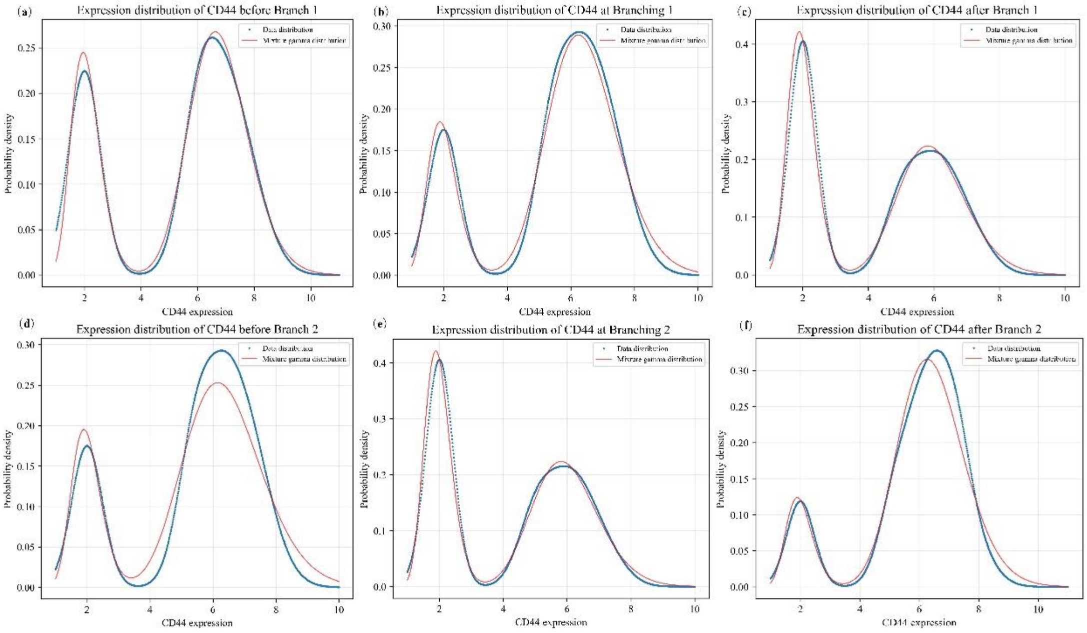

Fig. S14 Different modes of the mRNA distributions for key gene CD44 before and after branch as well as at branching point. The blue dashed curves represent the distributions of data points while the red curves represent the fitting results obtained using mechanic models of gene expression.

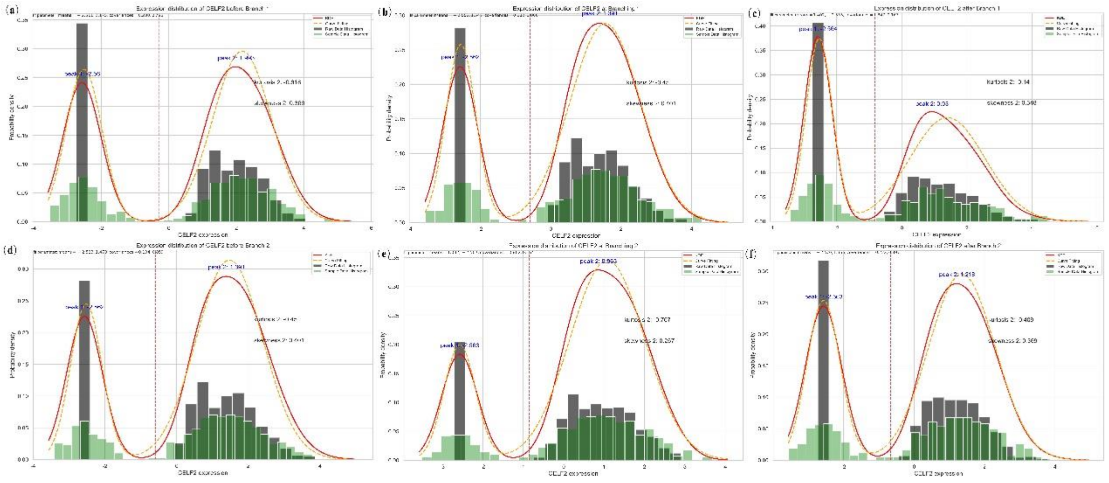

Fig. S15 Statistical quantities for the CELF2 gene before branch 1, at branching point 1, and after branch 1 and branch before branch 2, at branching point 2, after branch 2.

Table S6. The inferred parameter values for the CELF2 gene before branch 1, at branching point 1, and after branch 1 and before branch 2, at branching point 1, and after branch 2.

	Distribution type	parameter				
		$\omega 1$	$\alpha 1$	$\beta 1$	$\alpha 2$	$\beta 2$
Before branch 1	Bimodal mRNA distribution	0.6622	48.7593	7.2411	13.2580	6.5920
Branch point 1	Bimodal mRNA distribution	0.7238	39.695	6.4558	16.9998	8.2508
After branch 1	Bimodal mRNA distribution	0.4044	21.2323	10.6044	24.9344	4.1927
Before branch 2	Bimodal mRNA distribution	0.2778	17.6067	8.7570	34.1300	5.5468
Branch point 2	Bimodal mRNA distribution	0.4044	21.2323	10.6044	24.9344	4.1927
After branch 2	Bimodal mRNA distribution	0.6882	38.4897	6.4641	15.7893	7.6394

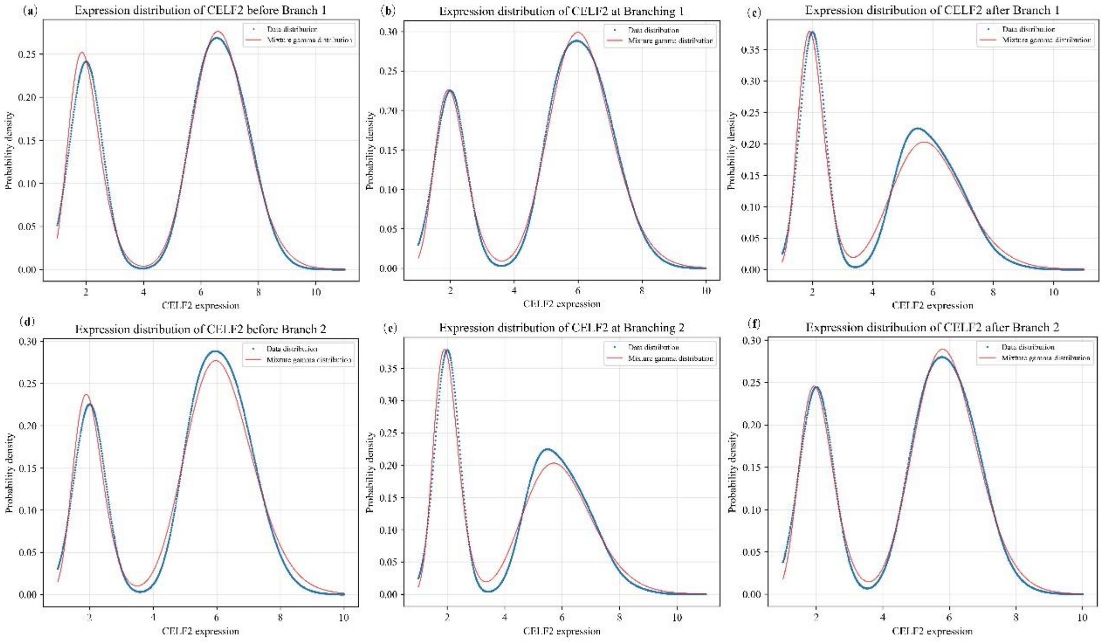

Fig. S16 Different modes of the mRNA distributions for key gene CELF2 before and after branch as well as at branching point. The blue dashed curves represent the distributions of data points while the red curves represent the fitting results obtained using mechanic models of gene expression.

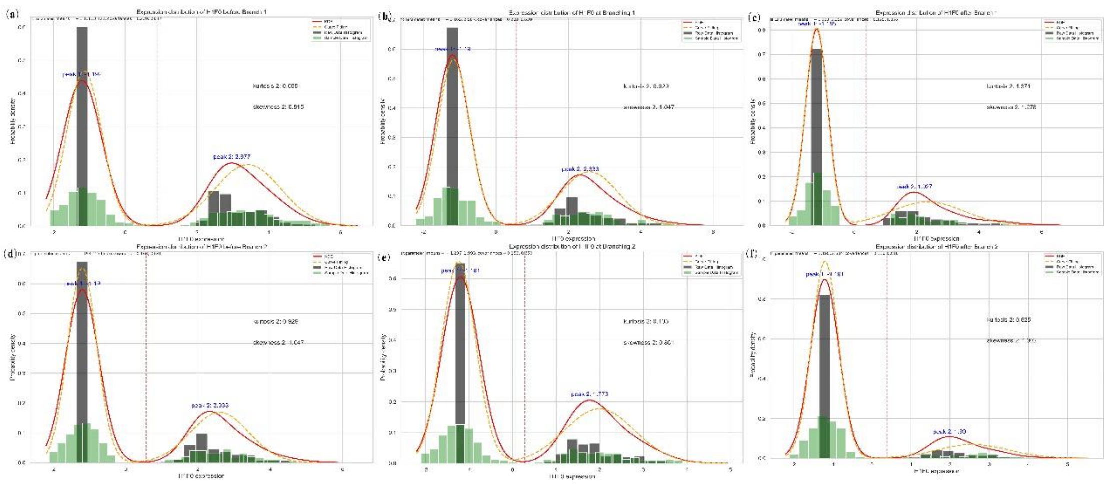

Fig. S17 Statistical quantities for the H1F0 gene before branch 1, at branching point 1, and after branch 1 and branch before branch 2, at branching point 2, after branch 2.

Table S7. The inferred parameter values for the H1F0 gene before branch 1, at branching point 1, and after branch 1 and before branch 2, at branching point 1, and after branch 2.

	Distribution type	parameter				
		$\omega 1$	$\alpha 1$	$\beta 1$	$\alpha 2$	$\beta 2$
Before branch 1	Bimodal mRNA distribution	0.4053	54.4009	8.4818	15.6535	8.1766
Branch point 1	Bimodal mRNA distribution	0.6783	20.0605	9.9825	23.9011	4.0988
After branch 1	Bimodal mRNA distribution	0.7270	23.3733	11.7181	22.5725	3.9571
Before branch 2	Bimodal mRNA distribution	0.6783	20.0605	9.9825	23.9011	4.0988
Branch point 2	Bimodal mRNA distribution	0.7270	23.3733	11.7181	22.5725	3.9571
After branch 2	Bimodal mRNA distribution	0.8294	23.2577	11.6576	22.4327	4.0689

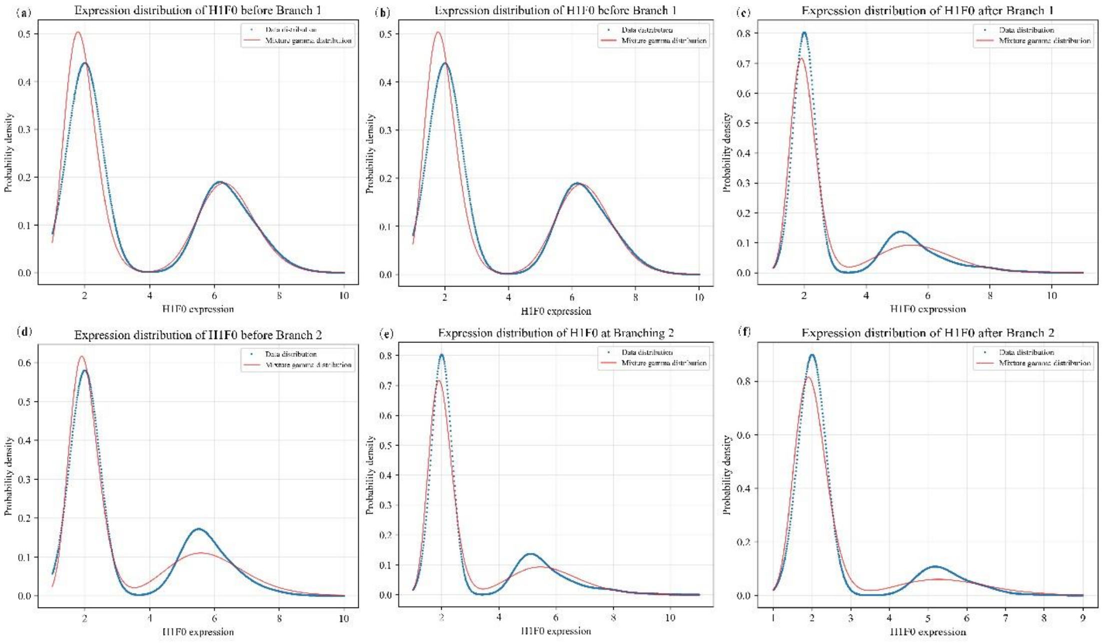

Fig. S18 Different modes of the mRNA distributions for key gene H1F0 before and after branch as well as at branching point. The blue dashed curves represent the distributions of data points while the red curves represent the fitting results obtained using mechanic models of gene expression.

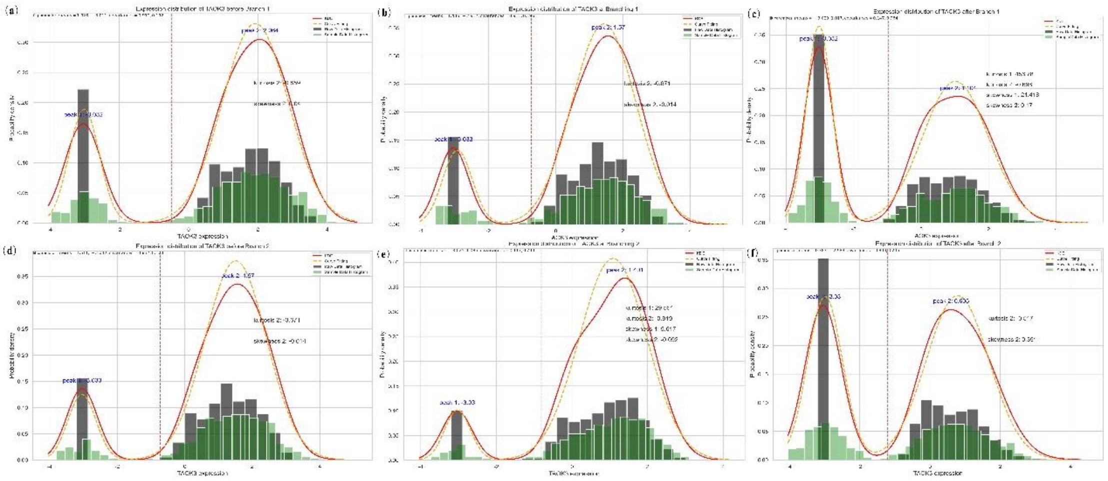

Fig. S19 Statistical quantities for the TAOK3 gene before branch 1, at branching point 1, and after branch 1 and branch before branch 2, at branching point 2, after branch 2.

Table S8. The inferred parameter values for the TAOK3 gene before branch 1, at branching point 1, and after branch 1 and before branch 2, at branching point 1, and after branch 2.

	Distribution type	parameter				
		$\omega 1$	$\alpha 1$	$\beta 1$	$\alpha 2$	$\beta 2$
Before branch 1	Bimodal mRNA distribution	0.2181	15.7200	7.7539	34.2096	4.8625
Branch point 1	Bimodal mRNA distribution	0.1554	20.3670	10.1224	34.2496	5.2514
After branch 1	Bimodal mRNA distribution	0.3496	22.5168	11.3067	34.0011	5.5855
Before branch 2	Bimodal mRNA distribution	0.1554	20.3670	10.1224	34.2496	5.2514
Branch point 2	Bimodal mRNA distribution	0.3456	22.6956	11.0988	33.4056	5.4644
After branch 2	Bimodal mRNA distribution	0.3477	16.1990	7.9937	34.1151	5.8239

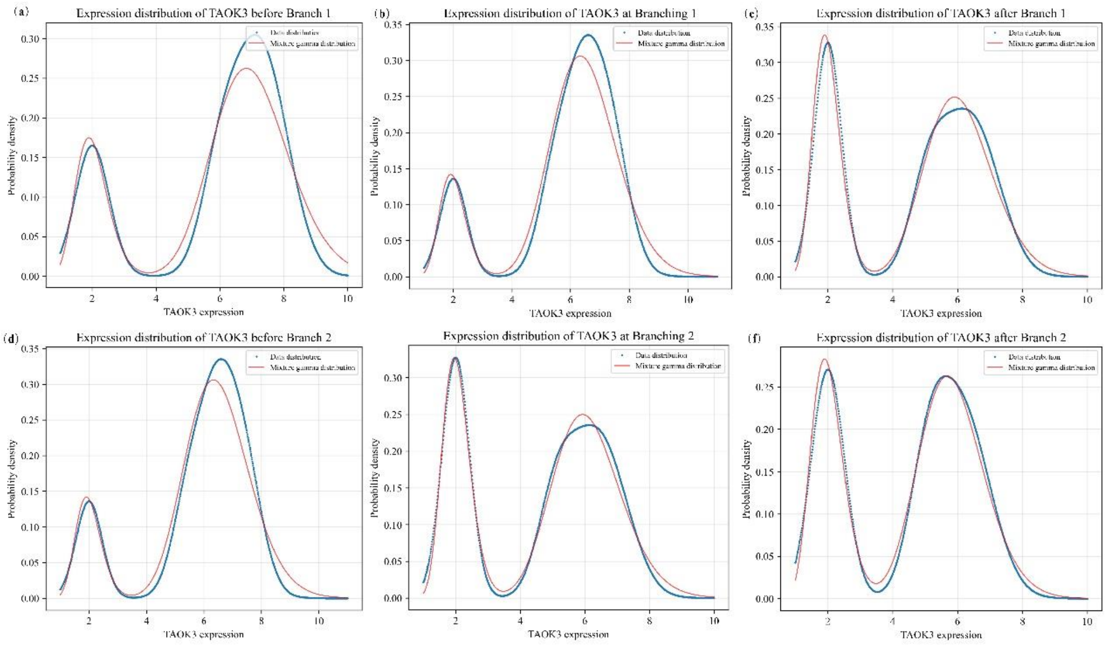

Fig. S20 Different modes of the mRNA distributions for key gene TAOK3 before and after branch as well as at branching point. The blue dashed curves represent the distributions of data points while the red curves represent the fitting results obtained using mechanic models of gene expression.

C5. Intestine organoid

Note that TA cells are an aggregation cluster; Enteroendocrine, Enteroendocrine progenitor, Goblet cells, Paneth cells and Stem cells are a kind of pre-aggregation cluster, whereas Enteroendocrine progenitor and Tuft cells are another kind of pre-aggregation cluster; and Enterocytes is the unique post-aggregation cluster. Refer to Fig. S21 for cell pseudo-trajectories constructed using scRNA-seq data, and Fig. S22-S28 and Table. S9-S22for the mRNA expression patterns of key genes along the pseudo-trajectories.

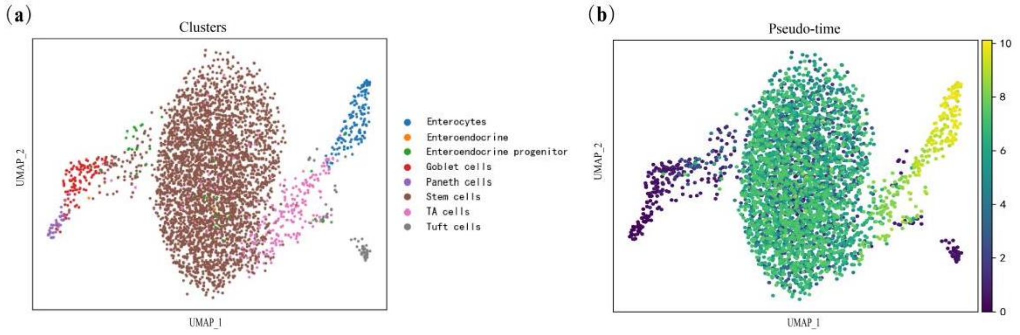

Fig. S21 Visualization of clustering and Pseudo-time inference clustering visualization in intestinal organoid dataset.

Table S9. Statistical quantities for the Atp2a3 gene before polymerization 1 and polymerization 2, at polymerization point, and after polymerization.

	Peak position	kurtosis	skewness
Pre-polymerization cluster 1	-0.005		

Pre-polymerization cluster 2	0.041	3.837	2.057
Polymeric cluster	-0.001		
Polymerized cluster	-0.001		

Table S10. The inferred parameter values for the Atp2a3 gene before polymerization 1 and polymerization 2, at polymerization point, and after polymerization.

	Distribution type	parameter		
		α	β	loc
Pre-polymerization cluster 1	Unimodal mRNA distribution	5876.3859	2366.0798	1.5166
Pre-polymerization cluster 2	Unimodal mRNA distribution	2.0007	1.0043	2.8594
Polymeric cluster	Unimodal mRNA distribution	12338.4374	4953.2721	1.5091
Polymerized cluster	Unimodal mRNA distribution	4800.8117	1453.0475	0.6964

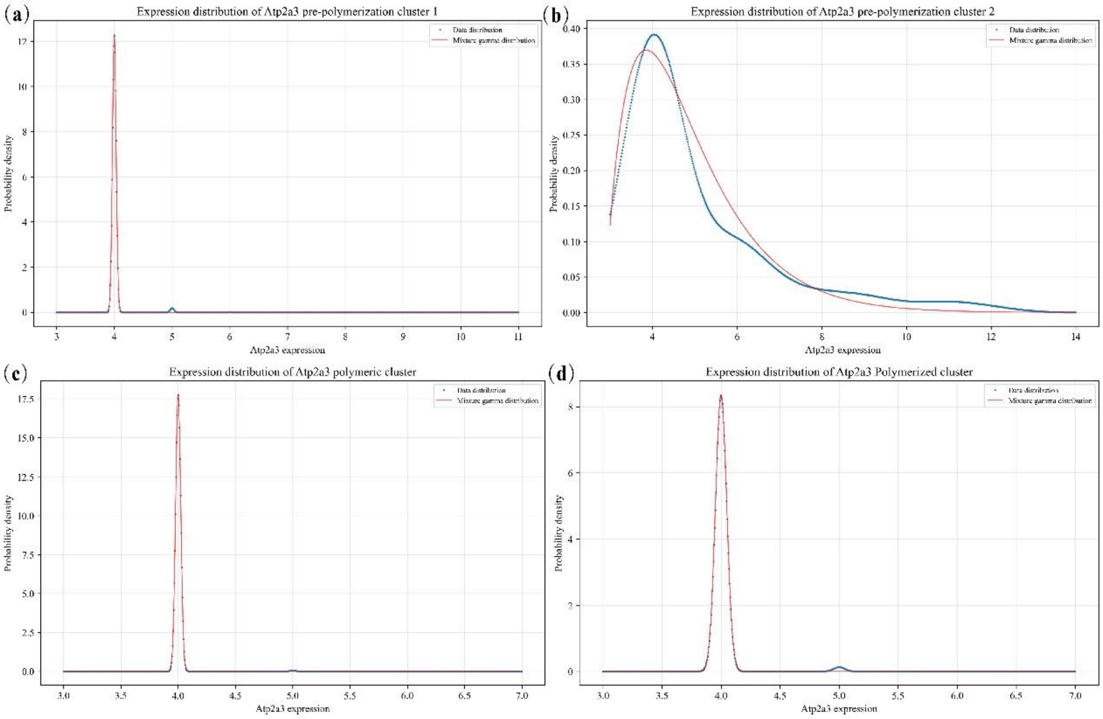

Fig. S22 Different modes of the mRNA distributions for key gene Atp2a3 before and after polymerization as well as at polymerization point. The blue dashed curves represent the distributions of data points while the red curves represent the fitting results obtained using mechanic models of gene expression.

Table S11. Statistical quantities for the Clu gene before polymerization 1 and polymerization 2, at polymerization point, and after polymerization.

	Peak position	kurtosis	skewness
Pre-polymerization cluster 1	0.019		
Pre-polymerization cluster 2	0.083	82.643	8.947

Polymeric cluster	0.003		
Polymerized cluster	0.043	20.188	4.081

Table S12. The inferred parameter values for the Clu gene before polymerization 1 and polymerization 2, at polymerization point, and after polymerization.

	Distribution type	parameter		
		α	β	loc
Pre-polymerization cluster 1	Unimodal mRNA distribution	4.5336	4.0261	3.0744
Pre-polymerization cluster 2	Unimodal mRNA distribution	3855.5196	22.7441	-165.4806
Polymeric cluster	Unimodal mRNA distribution	8.2927	6.5895	2.8567
Polymerized cluster	Unimodal mRNA distribution	5.5074	3.0307	2.5337

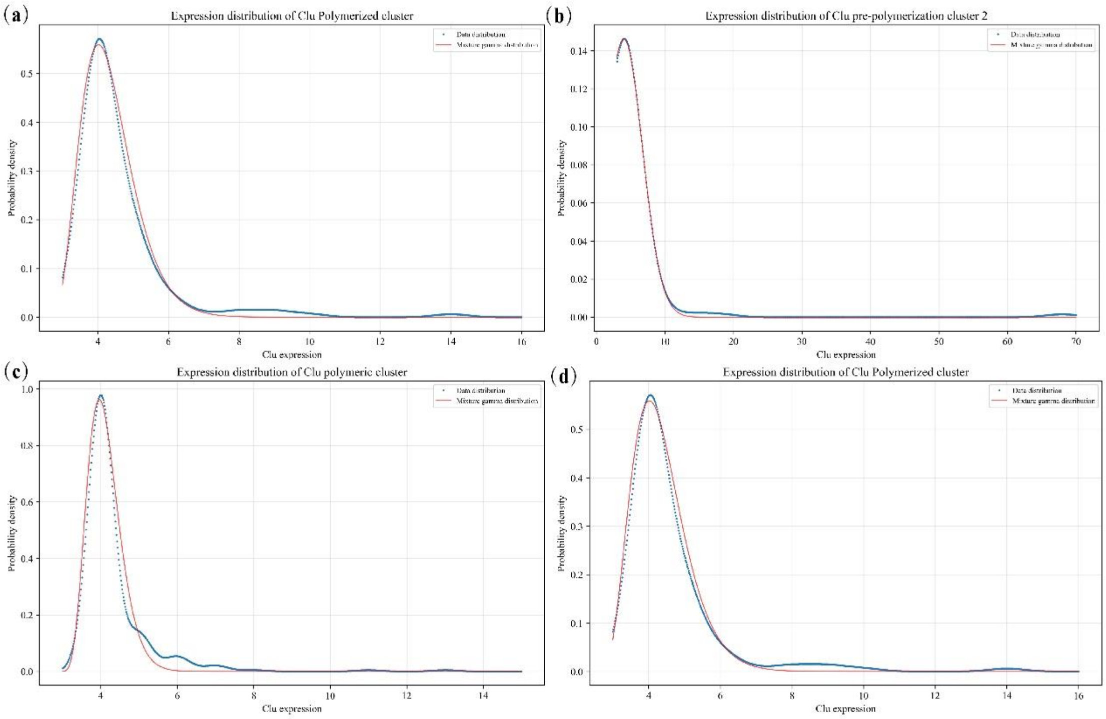

Fig. S23 Different modes of the mRNA distributions for key gene Clu before and after polymerization as well as at polymerization point. The blue dashed curves represent the distributions of data points while the red curves represent the fitting results obtained using mechanic models of gene expression.

Table S13. Statistical quantities for the Edn1 gene before polymerization 1 and polymerization 2, at polymerization point, and after polymerization.

	Peak position	kurtosis	skewness
Pre-polymerization cluster 1	0.006		
Pre-polymerization cluster 2	-0.005	61.845	7.616
Polymeric cluster	0.009	109.022	9.705
Polymerized cluster	0.003		

Table S14. The inferred parameter values for the Edn1 gene before polymerization 1 and polymerization 2, at polymerization point, and after polymerization.

	Distribution type	parameter		
		α	β	*loc*
Pre-polymerization cluster 1	Unimodal mRNA distribution	300.2292	74.9539	0.0021
Pre-polymerization cluster 2	Unimodal mRNA distribution	2402.3494	108.6915	-18.095
Polymeric cluster	Unimodal mRNA distribution	350.8701	55.7226	-2.284
Polymerized cluster	Unimodal mRNA distribution	2337.4154	219.2878	-6.6565

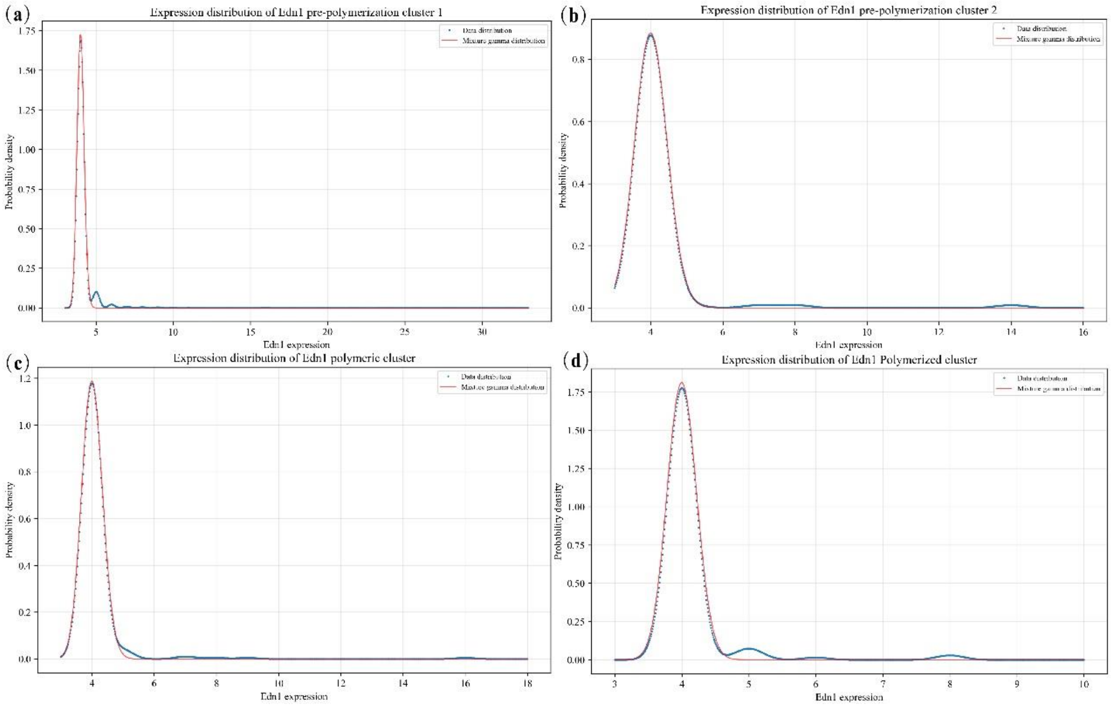

Fig. S24 Different modes of the mRNA distributions for key gene Edn1 before and after polymerization as well as at polymerization point. The blue dashed curves represent the distributions of data points while the red curves represent the fitting results obtained using mechanic models of gene expression.

Table S15. Statistical quantities for the Gsta4 gene before polymerization 1 and polymerization 2, at polymerization point, and after polymerization.

	Peak position	kurtosis	skewness
Pre-polymerization cluster 1	0.037	130.82	10.101
Pre-polymerization cluster 2	0.062	28.936	5.094
Polymeric cluster	0.63	16.686	3.275
Polymerized cluster	5.0	3.939	1.922

Table S16. The inferred parameter values for the Gsta4 gene before polymerization 1

and polymerization 2, at polymerization point, and after polymerization.

	Distribution type	parameter		
		α	β	*loc*
Pre-polymerization cluster 1	Unimodal mRNA distribution	15.5707	4.7497	0.9701
Pre-polymerization cluster 2	Unimodal mRNA distribution	2932.8522	51.3486	-53.0658
Polymeric cluster	Unimodal mRNA distribution	3.2182	0.5362	-0.1491
Polymerized cluster	Unimodal mRNA distribution	3.7053	0.192	-5.8868

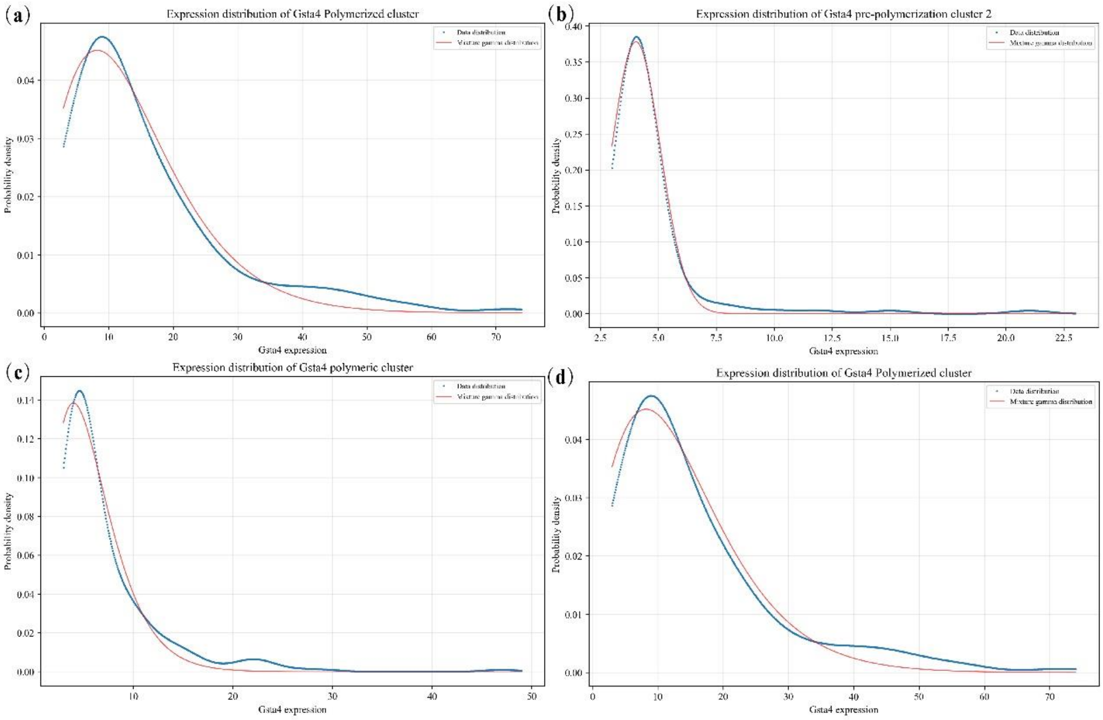

Fig. S25 Different modes of the mRNA distributions for key gene Gsta4 before and after polymerization as well as at polymerization point. The blue dashed curves represent the distributions of data points while the red curves represent the fitting results obtained using mechanic models of gene expression.

Table S17. Statistical quantities for the H1f0 gene before polymerization 1 and polymerization 2, at polymerization point, and after polymerization.

	Peak position	kurtosis	skewness
Pre-polymerization cluster 1	0.083	9.8	2.497
Pre-polymerization cluster 2	0.283	4.371	1.91
Polymeric cluster	0.508	8.118	2.553
Polymerized cluster	0.499	7.633	2.219

Table S18. The inferred parameter values for the H1f0 gene before polymerization 1 and polymerization 2, at polymerization point, and after polymerization.

	Distribution type	parameter		
		α	β	*loc*
Pre-polymerization cluster 1	Unimodal mRNA distribution	1.5985	0.6919	3.2664
Pre-polymerization cluster 2	Unimodal mRNA distribution	2.0416	0.8262	2.8655
Polymeric cluster	Unimodal mRNA distribution	2.3019	0.6628	2.4069
Polymerized cluster	Unimodal mRNA distribution	3.1401	0.8491	2.187

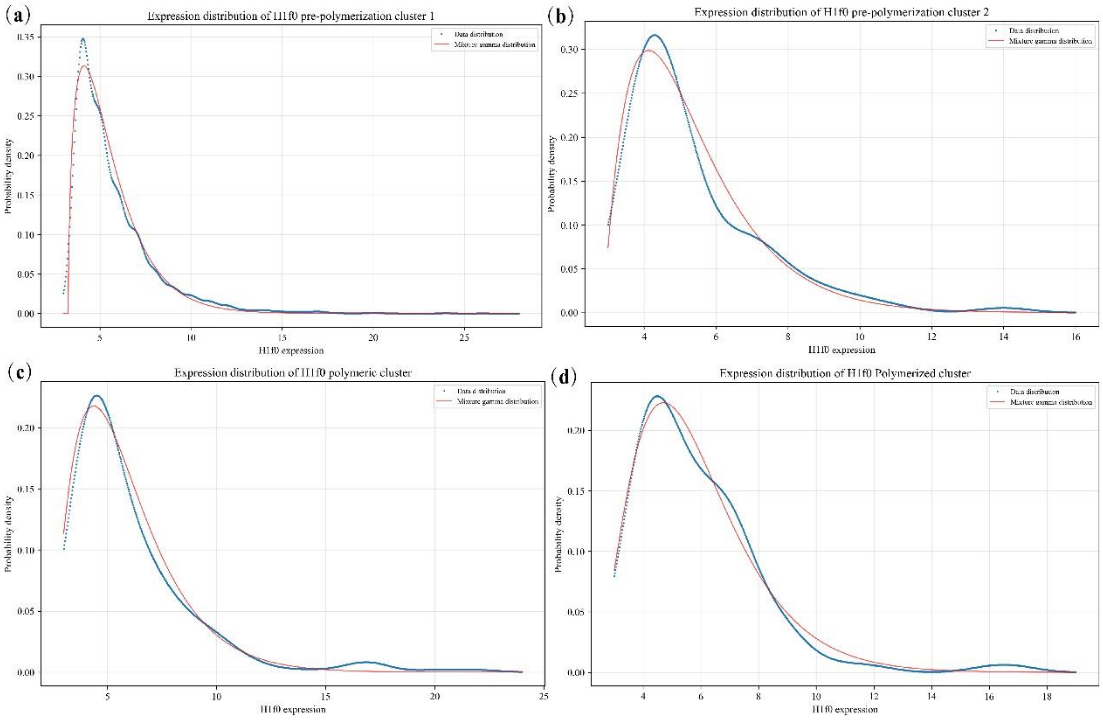

Fig. S26 Different modes of the mRNA distributions for key gene H1f0 before and after polymerization as well as at polymerization point. The blue dashed curves represent the distributions of data points while the red curves represent the fitting results obtained using mechanic models of gene expression.

Table S19. Statistical quantities for the Muc13 gene before polymerization 1 and polymerization 2, at polymerization point, and after polymerization.

	Peak position	kurtosis	skewness
Pre-polymerization cluster 1	0.069	32.169	4.274
Pre-polymerization cluster 2	0.589	1.328	1.252
Polymeric cluster	0.432	4.264	1.941
Polymerized cluster	3.39	4.184	1.686

Table S20. The inferred parameter values for the Muc13 gene before polymerization 1 and polymerization 2, at polymerization point, and after polymerization.

	Distribution type	parameter		
		α	β	*loc*

Pre-polymerization cluster 1	Unimodal mRNA distribution	1.8563	0.9878	3.2122
Pre-polymerization cluster 2	Unimodal mRNA distribution	2.3877	0.7052	2.661
Polymeric cluster	Unimodal mRNA distribution	2.2348	0.7132	2.6808
Polymerized cluster	Unimodal mRNA distribution	2.4181	0.2671	1.6263

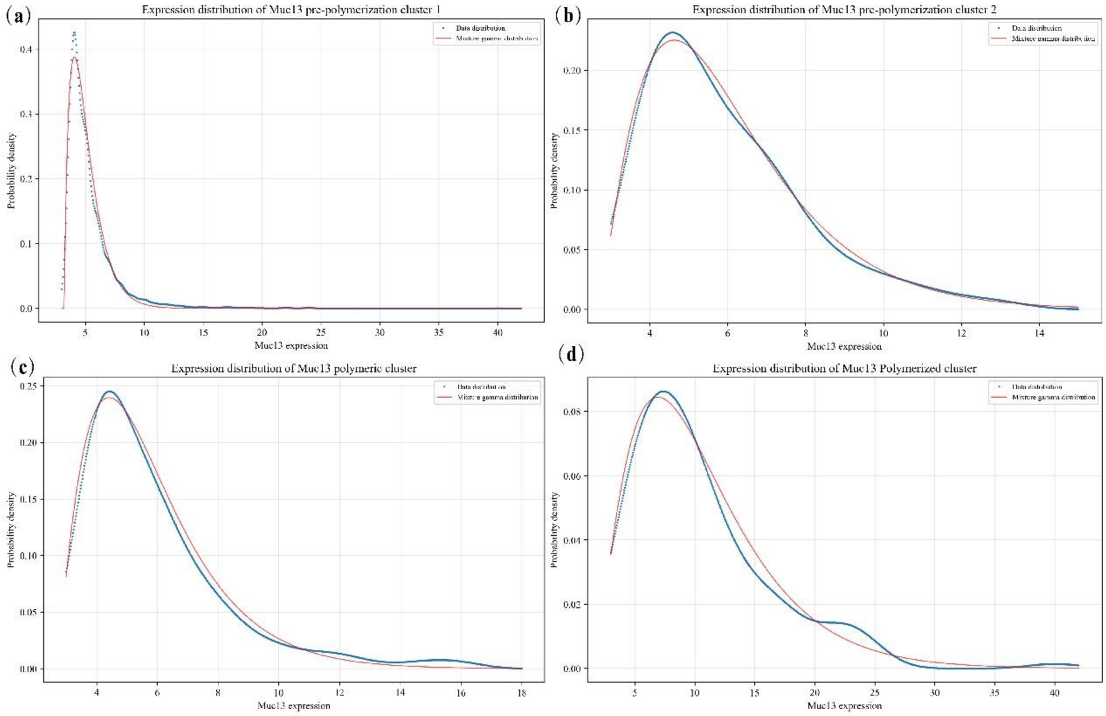

Fig. S27 Different modes of the mRNA distributions for key gene Muc13 before and after polymerization as well as at polymerization point. The blue dashed curves represent the distributions of data points while the red curves represent the fitting results obtained using mechanic models of gene expression.

Table S21. Statistical quantities for the Ndrg1 gene before polymerization 1 and polymerization 2, at polymerization point, and after polymerization.

	Peak position	kurtosis	skewness
Pre-polymerization cluster 1	0.003	88.0	8.477
Pre-polymerization cluster 2	0.003		
Polymeric cluster	0.127	117.848	9.952
Polymerized cluster	2.315	1.008	1.259

Table S22. The inferred parameter values for the Ndrg1 gene before polymerization 1 and polymerization 2, at polymerization point, and after polymerization.

	Distribution type	parameter		
		α	β	loc
Pre-polymerization cluster 1	Unimodal mRNA distribution	31.5704	15.8968	2.0584

Pre-polymerization cluster 2	Unimodal mRNA distribution	2.1508	1.7797	3.286
Polymeric cluster	Unimodal mRNA distribution	54.1244	6.1601	-4.5141
Polymerized cluster	Unimodal mRNA distribution	2.0912	0.2423	1.5628

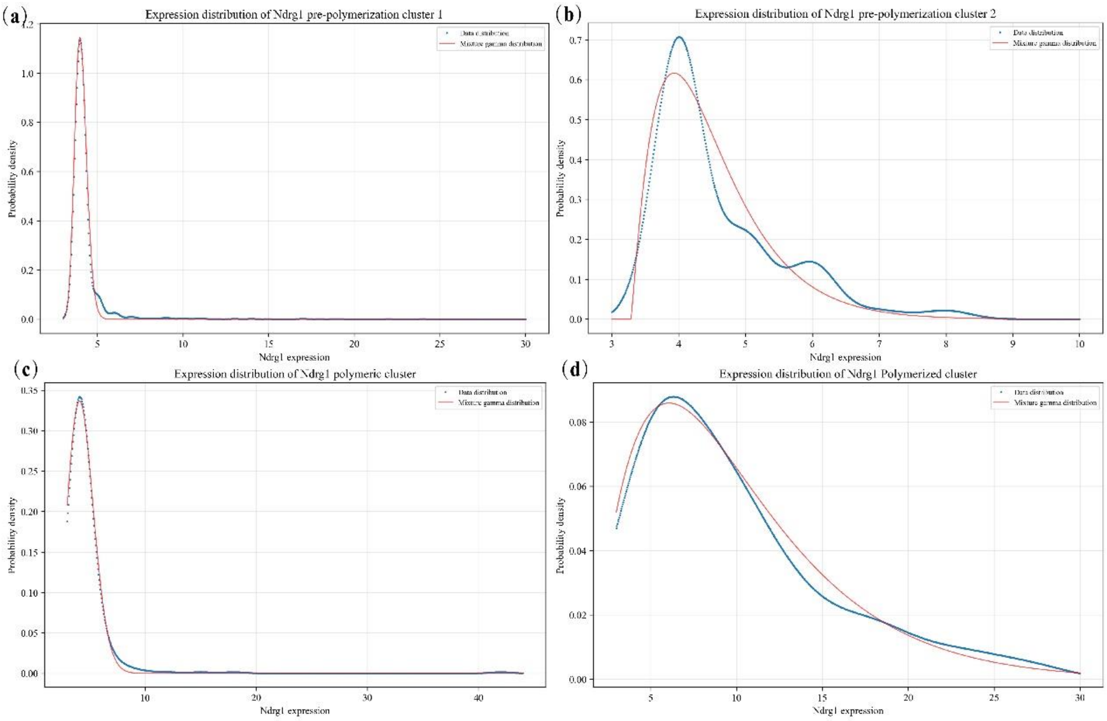

Fig. S28 Different modes of the mRNA distributions for key gene Ndrg1 before and after polymerization as well as at polymerization point. The blue dashed curves represent the distributions of data points while the red curves represent the fitting results obtained using mechanic models of gene expression.

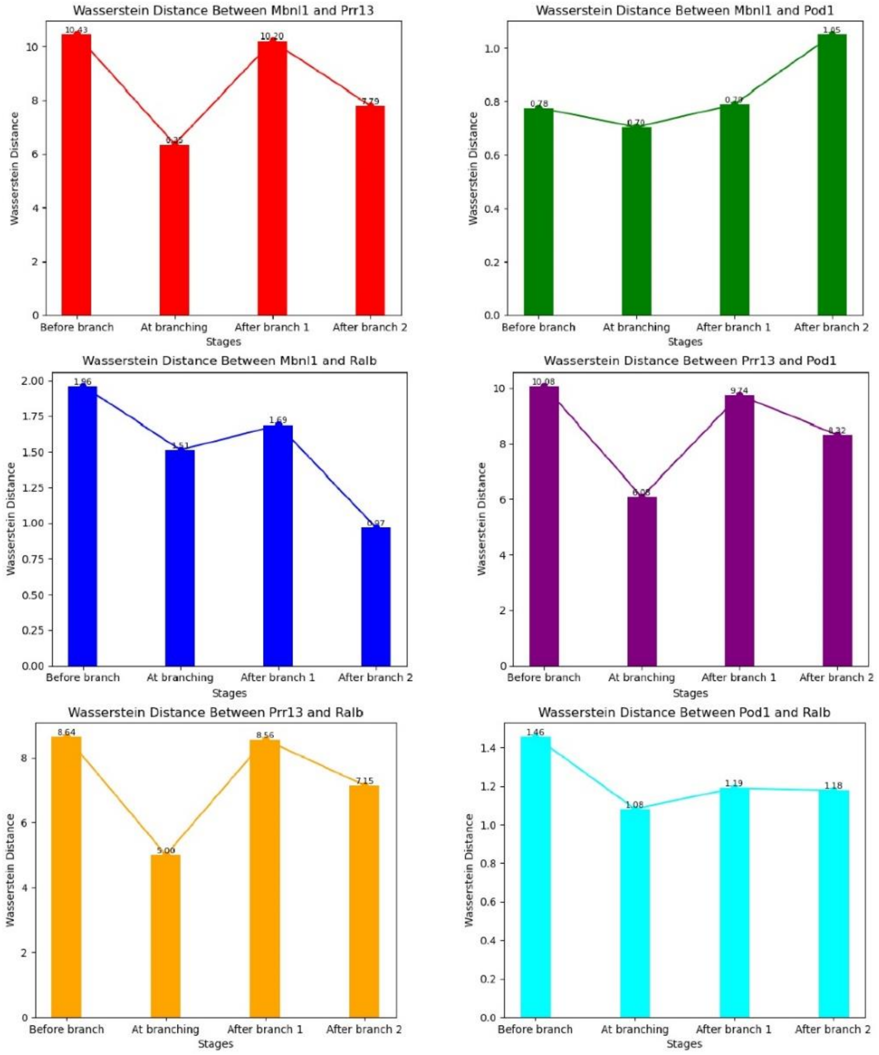

Fig. S29 Wasserstein distances between Mbnl1, Pod1, Ralb and Prr13 in different stages in MEF dataset.

D. Supplemental tables

Table S23. Wasserstein Distance Between Two Genes in Different Stages in human bone marrow dataset.

Stages / Wasserstein Distance	Pre-polymerization cluster 1	pre-polymerization cluster 2	Polymeric cluster	Polymerized cluster
Gsta4 and Muc13	0.14	0.32	0.24	0.60
Gsta4 and Ndrg1	0.27	0.24	0.31	0.52
Gsta4 and Slc7a9	0.28	0.11	0.12	0.30
Gsta4 and Clu	0.67	0.15	0.80	0.15
Gsta4 and Edn1	0.15	0.08	0.25	0.48
Gsta4 and Atp2a3	0.14	0.07	0.07	0.38
Gsta4 and H1f0	0.17	0.21	0.26	0.63
Muc13 and Ndrg1	0.13	0.09	0.07	0.08
Muc13 and Slc7a9	0.43	0.43	0.36	0.30
Muc13 and Clu	0.81	0.47	1.04	1.66

Muc13 and Edn1	0.01	0.34	0.01	0.13
Muc13 and Atp2a3	0.28	0.33	0.29	0.23
Muc13 and H1f0	0.31	0.12	0.04	0.03
Ndrg1 and Slc7a9	0.55	0.34	0.43	0.22
Ndrg1 and Clu	0.94	0.38	1.11	1.58
Ndrg1 and Edn1	0.12	0.25	0.07	0.05
Ndrg1 and Atp2a3	0.41	0.25	0.37	0.15
Ndrg1 and H1f0	0.44	0.03	0.05	0.11
Slc7a9 and Clu	0.39	0.06	0.68	1.36
Slc7a9 and Edn1	0.44	0.15	0.36	0.17
Slc7a9 and Atp2a3	0.15	0.10	0.09	0.07
Slc7a9 and H1f0	0.11	0.31	0.38	0.33
Clu and Edn1	0.82	0.15	1.04	1.53
Clu and Atp2a3	0.53	0.14	0.74	1.43
Clu and H1f0	0.50	0.35	1.06	1.69
Edn1 and Atp2a3	0.29	0.07	0.30	0.10
Edn1 and H1f0	0.32	0.23	0.04	0.16
Atp2a3 and H1f0	0.03	0.22	0.32	0.26

Table S24. Wasserstein Distance Between Two Genes in Different Stages in intestine organoid dataset.

Stages / Wasserstein Distance	Before branch 1	At branching 1	After branch 1
ATP2A3 and H1F0	1.27	1.35	1.29
ATP2A3 and CD44	1.27	1.35	1.28
ATP2A3 and CELF2	1.24	1.32	1.25
ATP2A3 and TAOK3	0.75	0.70	0.53
ATP2A3 and CNN3	0.97	1.07	1.01
ATP2A3 and SCUBE2	1.18	1.18	1.18
ATP2A3 and SMIM1	0.29	0.45	0.32
H1F0 and CD44	0.01	0.004	0.01
H1F0 and CELF2	0.05	0.05	0.06
H1F0 and TAOK3	0.63	0.77	0.81
H1F0 and CNN3	0.35	0.36	0.34
H1F0 and SCUBE2	0.11	0.20	0.13
H1F0 and SMIM1	1.03	1.01	1.00
CD44 and CELF2	0.05	0.05	0.05
CD44 and TAOK3	0.62	0.76	0.80
CD44 and CNN3	0.34	0.35	0.33
CD44 and SCUBE2	0.11	0.19	0.12
CD44 and SMIM1	1.02	1.00	0.97
CELF2 and TAOK3	0.59	0.73	0.77
CELF2 and CNN3	0.31	0.31	0.28
CELF2 and SCUBE2	0.06	0.15	0.07
CELF2 and SMIM1	0.99	0.97	0.96
TAOK3 and CNN3	0.30	0.44	0.51
TAOK3 and SCUBE2	0.52	0.58	0.70
TAOK3 and SMIM1	0.48	0.28	0.23
CNN3 and SCUBE2	0.24	0.16	0.21
CNN3 and SMIM1	0.72	0.70	0.71
SCUBE2 and SMIM1	0.93	0.83	0.89

References

Daalhuis ABO. Confluent hypergeometric functions. *NIST handbook of mathematical functions.* 2010; **321**:349.

Gillespie DT. A general method for numerically simulating the stochastic time evolution of coupled chemical reactions. *J Comput Phys.* 1976; **22**:403-434.
